# A novel class of somatic mutations in blood detected preferentially in CD8 + cells

**DOI:** 10.1016/j.clim.2016.11.018

**Published:** 2017-02

**Authors:** Miko Valori, Lilja Jansson, Anna Kiviharju, Pekka Ellonen, Hanna Rajala, Shady Adnan Awad, Satu Mustjoki, Pentti J. Tienari

**Affiliations:** a Molecular Neurology, Research Programs Unit, University of Helsinki, Department of Neurology, Helsinki University Hospital, Haartmaninkatu 8, FIN-00290 Helsinki, Finland; b Finnish Institute for Molecular Medicine (FIMM), University of Helsinki, Helsinki, Finland; c Hematology Research Unit Helsinki, University of Helsinki and Helsinki University Hospital Comprehensive Cancer Center, Haartmaninkatu 8, FIN-00029 Helsinki, Finland; d Department of Clinical Chemistry, University of Helsinki, Helsinki, Finland

**Keywords:** Somatic mutation, Autoimmune disease, Multiple sclerosis, CD8, STAT3

## Abstract

Somatic mutations have a central role in cancer but their role in other diseases such as autoimmune disorders is poorly understood. Earlier work has provided indirect evidence of rare somatic mutations in autoreactive T-lymphocytes in multiple sclerosis (MS) patients but such mutations have not been identified thus far. We analysed somatic mutations in blood in 16 patients with relapsing MS and 4 with other neurological autoimmune disease. To facilitate the detection of somatic mutations CD4 +, CD8 +, CD19 + and CD4 −/CD8 −/CD19 − cell subpopulations were separated. We performed next-generation DNA sequencing targeting 986 immune-related genes. Somatic mutations were called by comparing the sequence data of each cell subpopulation to other subpopulations of the same patient and validated by amplicon sequencing. We found non-synonymous somatic mutations in 12 (60%) patients (10 MS, 1 myasthenia gravis, 1 narcolepsy). There were 27 mutations, all different and mostly novel (67%). They were discovered at subpopulation-wise allelic fractions of 0.2%–4.6% (median 0.95%). Multiple mutations were found in 8 patients. The mutations were enriched in CD8 + cells (85% of mutations). In follow-up after a median time of 2.3 years, 96% of the mutations were still detectable. These results unravel a novel class of persistent somatic mutations, many of which were in genes that may play a role in autoimmunity (*ATM, BTK, CD46, CD180, CLIP2, HMMR, IKFZF3, ITGB3, KIR3DL2, MAPK10, CD56/NCAM1, RBM6, RORA*, *RPA1* and *STAT3*). Whether some of this class of mutations plays a role in disease is currently unclear, but these results define an interesting hitherto unknown research target for future studies.

## Highlights

• Nonsynonymous somatic mutations were detectable in the blood in more than half of the patients in this study.
• The mutant clones were rare, allelic fractions mostly < 0.5% of total peripheral blood mononuclear cells.
• 85% of mutations were found in CD8+ cells.
• A previously described activating STAT3*D661V mutation was found in CD8+ cells of a multiple sclerosis patient.
• The role of the mutations in autoimmunity is currently unclear but warrants further research.

## 1 Introduction

Somatic mutations are non-inherited alterations in DNA that occur in a progenitor cell and become transmitted to its descendant population (clone). They arise at a low frequency by chance, typically during DNA replication, and can be caused by environmental factors such as chemicals or ionizing radiation. Virtually all cancer cells have such acquired mutations and several types of somatic mutations in blood leukocytes have been characterized in e.g. leukemias [1].

Besides patients with hematological cancers, relatively little is known on the frequency and nature of somatic mutations in blood leukocytes. Age-related somatic mutations that confer growth advantage have been recently detected in blood in association with clonal hematopoiesis [2], [3], [4], [5]. Two large-scale studies have shed light to the landscape of blood cell somatic mutations occurring in population cohorts not selected for cancer or hematologic phenotypes [3], [4]. Both studies tested whole blood DNA and focused on mutations occurring with a large allelic fraction of ca. 10–20% in whole exome sequence data [3] or in a set of 160 genes implicated in hematologic cancers [4]. Somatic mutations were found in approximately 1% of subjects under age 50, and in 10% of subjects older than 70 years. Both studies identified mutational hotspots, *DNMT3A, TET2*, and *ASXL* mutations constituted > 2/3 of the mutations (2, 3).

There are only few studies on leukocyte somatic mutations in autoimmune disease (other than somatic hypermutation of immunoglobulin genes). Holzelova et al. [6] reported somatic mutations in *FAS* in a fraction of CD4 + and CD8 + T-lymphocytes in children with an autoimmune lymphoproliferative syndrome. Similar rare autoimmune diseases have been described in conjunction with somatic mutations in the *NRAS* and *KRAS* genes [7]. Whether somatic mutations have a role in more common autoimmune disorders has not been established. A recent study by us identified somatic mutations in the *STAT3* gene in CD8 + T-cells in 40% of patients with large granular lymphocytic leukemia [8]. Interestingly, *STAT3* mutation positive patients presented with rheumatoid arthritis significantly more often than mutation negative patients, suggesting a possible role of *STAT3* mutations in these autoimmune symptoms.

Multiple sclerosis (MS) is a chronic inflammatory disease of the central nervous system and among the most common causes of neurological disability in young adults. The cause of MS is not known, but it is assumed to be an autoimmune disorder. Multiple lines of evidence suggest that at least relapsing forms of MS would be driven by blood leukocyte dysfunction. > 100 genetic loci are known to be linked with MS predisposition, the overwhelming majority of which are in genes or regions active in leukocytes [9], [10]. The most potent drugs for relapsing MS (natalizumab, alemtuzumab, fingolimod, daclizumab) are mainly targeted against lymphocytes circulating in the blood [11]. Moreover, Epstein-Barr virus (EBV) is a well-documented environmental risk factor of both MS as well as lymphoid and epithelial malignancies, the risk of MS being especially high in association with EBV infectious mononucleosis [12].

Against this background, it is of interest to analyze blood leukocyte somatic mutations in MS. Indirect evidence of somatic mutations has been previously reported in MS by using the hypoxanthine guanine phosphoribosyltransferase (HPRT) assay [13], [14], [15]. This assay measures 6-thioguanine resistance of cultured cells, caused by inactivating somatic mutations of the *HPRT* gene or other mechanisms. The estimated mutation frequency in T-lymphocytes is approximately 5 × 10^− 6^ in adults [14]. Allegretta et al. reported on the presence of HPRT-deficient T-lymphocyte clones in MS and, interestingly, some of these mutant clones, but not wild-type clones, were potentially autoreactive, i.e. proliferated in response to a myelin autoantigen [13]. Another study reported higher HPRT mutant frequency in MS patients' T lymphocytes as compared to controls [15]. A pilot study on direct detection of somatic mutations in MS has been reported [16]. The study was focused on single CD4 + T-lymphocytes, derived from two MS patients' cerebrospinal fluid. In an exome sequencing analysis of 21 individual whole genome amplified cells thousands of variants were reported, but the authors concluded that they were unable to confidently identify true somatic mutations, as these could not be discerned from whole genome amplification induced mutational noise. The study points to technical obstacles when mutations in small cell populations are screened.

In the present study we analysed whether nonsynonymous somatic mutations can be reliably found in the blood. We enriched peripheral blood mononuclear cells (PBMCs) into subpopulations and utilized next-generation sequencing of 986 immune-related genes (with additional validation sequencing), allowing the detection of mutations with low allelic fractions (down to 0.1–0.2%). We addressed the following questions. Are somatic mutations detectable with this approach? What is their frequency among the patients and allelic fraction within cell populations? Do the mutations preferentially occur in a particular cell population? Are there mutational hotspots? This information would provide important insights into the design of studies in larger materials and reveal details about the general landscape of blood somatic mutations in non-cancerous settings.

## 2 Results

### 2.1 Screening for somatic mutations in peripheral blood mononuclear cell populations

We obtained blood samples from 20 patients. 16 of the patients had relapsing MS, 2 had narcolepsy, one had myasthenia gravis, and one had stiff person syndrome and systemic lupus erythematosus (clinical details given in Table 1). We used immunomagnetic beads to enrich PBMCs into CD4 +, CD8 + and CD19 + cell populations and a remaining CD4 − CD8 − CD19 − triple negative cell population. These markers were chosen to capture basic subgroups of interest.

We used high-depth next generation sequencing (NGS) to screen the DNAs for somatic mutations. The sequencing covered all exons of a panel of 986 immune-related genes (Table S1). We restricted our sequencing efforts to this predefined panel (referred to as Immunopanel-986 from hereon) in order to cost-effectively approach a target sequencing depth of up to 1000 ×. In total we successfully sequenced 71 cell populations obtained from the PBMCs of the 20 patients (mean depths within coding regions: CD4 + 753x, CD8 + 723x, CD19 + 671x, CD4 − CD8 − CD19 − 491x). From this Immunopanel-986 sequencing data, we performed somatic variant calling to find variants that were detectable in a particular cell population and were not present in other (control) cell populations of the same patient (more details in Materials and Methods, Screening phase data analysis). We were able to produce a list of 154 putative somatic mutations for further validation using another sequencing method (amplicon sequencing).

### 2.2 Amplicon sequencing confirms 27 nonsynonymous somatic mutations

Using DNA from the same cell populations in which the putative somatic variants were called, we prepared validation PCR amplicons corresponding to the 154 putative nonsynonymous somatic variant calls. We again employed NGS and sequenced the patient DNA amplicons along with amplicons prepared from control DNA to an excessive mean depth of over 100,000 ×. We were able validate 27 of the 154 variant calls as true somatic mutations. Both screening and validation step sequencing depth and allelic fractions for each confirmed mutation are given in Table S2, along with p-values for the somatic mutation calls.

A subset of 8 mutations were tested in two PCR replicates from whole blood DNA. All mutations were detectable in whole blood DNA in both replicates. The mean allelic fraction of these 8 mutations was 0.35% in whole blood as compared to 2.25% (6.4 fold higher) in the enriched subpopulation of cells (Supplementary Table S3).

A technical reason for the inclusion of so many false positives (n = 127; 82%) was found to be counting some sequence mismatches twice, when the two opposite direction reads of paired end sequencing overlapped on short DNA fragments. This inflated the read count for some rare observations. In a post hoc analysis, we observed that counting the mismatches present on such overlapping paired end reads only once per pair eliminated the false positives, although with a cost in sensitivity (missing 16 of the 27 true somatic variants).

### 2.3 Somatic mutation frequencies

Out of the 20 patients, we were able to validate somatic mutations in 12 (60%). Eight of the 12 patients had multiple mutations (2–4 mutations per patient, Table 2). Ten of the patients with somatic mutations had MS, while the other two had myasthenia gravis and narcolepsy.

**Fig. 1:**
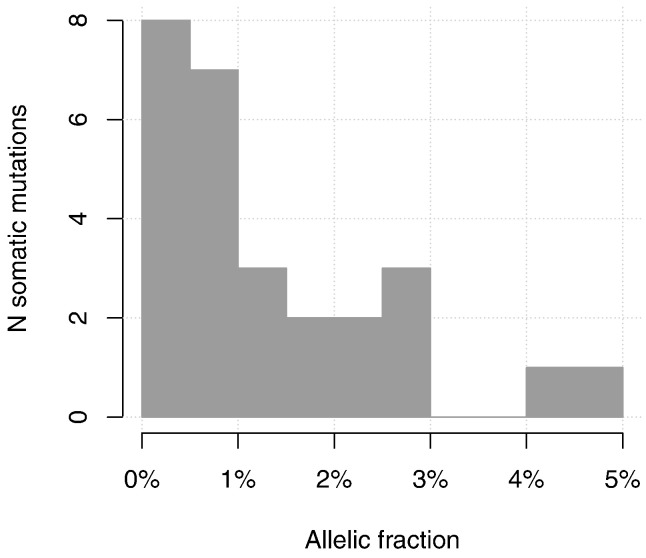
Distribution of the allelic fractions of validated somatic mutations.

The highest allelic fraction observed for a somatic mutation was 4.6%, which would correspond to a clone size of 9.2% assuming a heterozygous mutation. The lowest allelic fraction of a somatic mutation was 0.19% while the median allelic fraction of all validated mutations was 0.95%. Fig. 1 shows the distribution of allelic fractions.

### 2.4 Unique and predominantly novel mutations in each patient

Table 3 shows the 27 validated somatic mutations. In total, we observed 23 single nucleotide variants (SNV) that were predicted to result in an amino acid changes, 2 in-frame 3-bp deletions, one splice-site SNV, and one premature stop-codon producing SNV. Each patient had a unique mutation profile – there were no mutational hotspots. Two patients had mutations in the CD56/NCAM1 gene, but the mutations were at different sites.

Out of the 27 somatic mutations, 19 (67%) were novel, i.e. they were not found in COSMIC [17], dbSNP [18] or ExAC [19] databases, whereas 8 of the somatic mutations could be found in at least one of these databases. Nineteen (70%) of the mutations were predicted deleterious by the CADD [20] algorithm (Table 3).

We observed no association between patient's age (p = 0.23, two-tailed *t*-test) or disease duration (p = 0.93) and mutation positivity in this dataset. Normal expression of the genes, in which mutations were detected was analysed by RNA sequencing in reference cell populations sorted according to the same protocol as all study samples (the expression status is given in Table 3 and RNA sequencing expression values in Table S4).

### 2.5 Enrichment of somatic mutations in the CD8 + cell population

Intriguingly, the clear majority (n = 23, 85%) of the 27 somatic mutations were observed in the CD8 + cell population. Although we sequenced a similar amount of CD4 + and CD19 + cells (based on sequencing depths), we were not able to validate more than a single somatic mutation in each of these cell populations (Table 4).

For the CD8 + and CD4 + cell populations, we performed T cell receptor Vβ fluorescence-activated cell sorting (FACS) analysis to characterize clone sizes in the patient samples. Ten patients (50%) had an expanded “large” CD8 + clone (see definition in Table S5 footnote), but the presence of a large clone did not significantly predict the detection of a somatic mutation (p = 0.65, Table S5). Hence, preselection of the samples by T cell receptor Vβ FACS does not seem to facilitate the detection of this type of mutant clones with low allelic fractions. A representative T cell receptor Vβ FACS analysis of one patient (MS-8) is given in Supplementary Fig. S1. The Vβ quantifications of all 20 patients are given in Supplementary Fig. S2.

### 2.6 Persistence of detected somatic mutations

We collected a second blood sample from each of the patients whose initial sample contained one or more somatic mutations (median sampling interval 2.3 years). We separated the cell populations from PBMCs and again performed high depth amplicon sequencing in order to examine whether the somatic mutations persisted. All but one (26 of 27) of the somatic mutations were detected in the second sample. There was no general trend towards increase or decrease in allelic fraction: 14 mutations increased and 12 decreased in allelic fraction (including one, which was not anymore detectable), while one remained at the same level. The increases and decreases were mostly 50% or less in magnitude (in 19 of the 27 mutations). Table S6 shows the detailed changes in somatic mutation allelic fractions over time. One mutated CD8 + clone (carrying a deletion in the *IKZF3* gene) increased 7-fold in allelic fraction from 2.3% to 16.4%. This MS patient had changed treatment from baseline natalizumab, which induced lymphocytosis (blood lymphocyte count was 4.09 × 10^− 6^/ml) to fingolimod, which induced lymphopenia (blood lymphocyte count was 0.66 × 10^− 6^/ml). This data suggest that fingolimod targets other CD8 + populations more efficiently than the mutant clone. P-values and related observation counts for persistence data are listed in Table S7.

### 2.7 Autoimmunity associated STAT3*D661Y mutation in CD8 + cells of an MS patient

A notable observation was the presence of the STAT3*D661Y mutation in the CD8 + subpopulation of an MS patient, at an allelic fraction of 0.45% upon discovery and 0.74% in follow-up 19 months later (Table 3, Table S6). This mutation has been reported in large granular lymphocytic leukemia patients' CD8 + T-lymphocytes and among these patients this particular mutation associates with neutropenia and autoimmune rheumatoid arthritis [8], [21]. The MS patient with this mutation did not have neutropenia or any other clear abnormalities in blood (data not shown), nor did she have rheumatoid arthritis. She responded poorly to treatments. She developed a relapse during the initial beta-interferon treatment. Next she was treated with natalizumab, but developed progressive disease course during treatment and a relapse after cessation of natalizumab. Then, she started dimethyl fumarate treatment, but developed side effects (nausea, diarrhea, tachycardia) and during subsequent fingolimod treatment she developed gradual disease progression. Currently she is on teriflunomide (started 2 months ago) and has not had any relapses, disease progression or significant side-effects.

## 3 Discussion

We tested, whether somatic mutations can be detected in peripheral blood mononuclear cells in patients with neurological autoimmune disease, most of whom had MS. In contrast to previous population studies [3], [4] where mutations of allelic fraction above 10% were discovered in whole blood, we applied a discovery pipeline for lower allelic fractions. By enriching blood mononuclear cells into CD4 +, CD8 +, CD19 + and other subsets and applying high depth next-generation sequencing of 986 immune-related genes we were able to detect and validate persistent somatic mutations in over half of the patients enrolled. Mutations were found both in MS and other neurological autoimmune disease patients in the present study. The initially discovered mutations were present at allelic fractions between 0.19%–4.5% in each cell population. In whole blood the estimated allelic fractions would be < 0.5% for most of the mutations. We confirmed this estimate by directly sequencing 8 selected mutations from whole blood, where the observed mean allelic fraction was 0.34% (range 0.06–0.56%). These results demonstrate that high depth next generation sequencing is a viable way to discover somatic mutations from non-cancer patients' blood samples, even when the mutant clones are small. Importantly, the results define an interesting hitherto unknown research target for future studies. The major limitation of our study is that at this stage we cannot make any conclusions on the pathogenic role of the mutations. First, we did not analyze healthy controls and it is presently not known, if persistent somatic mutations in CD8 + cells are also common in healthy adults. Secondly, we do not know, if some of the mutant clones are also present in the central nervous system. Third, it is unclear, if some of these clones are able to react against own tissue either via autoantigen recognition or bystander demyelination [22]. Hence, further steps are needed to demonstrate pathogenicity of the mutant clones. Recent analyses of MS patient's T-cell receptor Vβ repertoire suggest that the same CD8 + clones present in MS plaques can also be detected in the cerebrospinal fluid and blood [23]. Further studies seem therefore realistic and are required to test the possible role of the mutated clones in disease, including sequencing of controls, patients' cerebrospinal fluid cells as well as analysis of antigen specificities and other phenotypic features of the mutant clones.

The mean sequencing depth at sites where validated somatic mutations were detected in the screening phase was 1062 × (median 934 ×) after duplicate read removal (Table S8). Therefore the target sequencing depth should be set to at least 1000 × in subsequent studies, with added depth beyond this expected to generate more findings. With more target genes/regions and more sequencing depth it is likely that more mutations would have been discovered in the present dataset. It should be noted that because of sequencing noise the depth cannot be increased indefinitely. The highest quality base calls that the Illumina platform produces have a quality score estimate of around 1/10,000 errors, which was found to be empirically quite accurate in our data. In order to find somatic mutations as rare as 1/10,000 and beyond, specialized techniques such as tagging DNA fragments with random barcodes [24] would have to be employed.

A striking finding in our study was the clear enrichment (85%) of the somatic mutations in the CD8 + cell population. This enrichment may stem from technical reasons (detectability) or reflect overall higher mutational load in CD8 + cells. CD8 + memory cells have been shown to produce larger clones than other populations [25] rendering mutations more readily detectable. On the other hand, mutagenesis itself may be especially prominent in CD8 + cells. CD8 + cells exhibit more clonal expansions upon antigen stimulus than CD4 + cells [26] and especially large clonal expansions of CD8 cells have been demonstrated in infectious mononucleosis [27]. More mitoses generate more mutations. Whether other biological reasons are also involved in the enrichment of mutation in CD8 cells (e.g. higher sensitivity to external mutagens, lower fidelity of DNA repair, lower rate of mutant clone elimination) is currently less clear. From an autoimmunity standpoint, activating mutations in CD8 + T cells are interesting because of these cells' role as autoimmune effectors in a mouse model [28], their high abundance in active MS lesions [29], and significant genetic effects of CD8 + cell's antigen recognition molecules, HLA class I in MS [30].

We did not detect any mutational hotspots in this data as each mutation was unique and all but two resided in distinct genes. A larger analysis with more target genes will be required to properly answer the question of hotspot existence. All mutations that we discovered were located in immune-related genes because we a priori selected only these genes in our panel. Because of this, to answer whether mutations accumulate in certain biological pathways would need a different type of study design. A few potentially interesting mutations were observed. The most prominent of these was the STAT3*D661Y mutation which is known to be present in large granular lymphocytic leukemia cases and associates with neutropenia and rheumatoid arthritis symptoms [8], [21]. Moreover, *STAT3* is one of the susceptibility loci in MS [31]. A shortlist of other interesting mutations, based on gene expression in the carrier cell population type (Table S4), mutation persistence (Table S6) and predicted deleteriousness, includes *ATM, BTK, CD46, CD180, CLIP2, HMMR, IKFZF3, ITGB3, KIR3DL2, MAPK10, CD56/NCAM1, RBM6, RORA*, and *RPA1* (Table 3). Previous studies utilizing the HPRT assay (12–14) used somatic mutation as an index of T-cell amplification to capture autoreactive clones for subsequent T-cell receptor and autoantigen characterization. Our present results demonstrate mutations that are potentially more than just indexes or genomic scars from past mitoses, their potential role in autoimmunity warrants further research.

## 4 Materials and methods

### 4.1 Study participants and sample collection

Patients for this study were recruited at the Department of Neurology of Helsinki University Hospital. This study has been approved by the regional ethics committee at the Helsinki University Hospital (Dno 83/13/03/01/2013). Each patient gave informed consent for participating in the research.

### 4.2 Cell separation and DNA extraction

Mononuclear cells were extracted from 120 to 140 ml venous EDTA blood using Ficoll-Paque PLUS (GE Healthcare Bio-Sciences, Piscataway, NJ, USA). First, 13 ml of Ficoll-Paque was added to a centrifuge tube. Then, 9 ml of blood diluted with 28 ml of PBS was layered on top of it. The tube was centrifuged at 800 ×* g* for 30 min after which the mononuclear cell layer was transferred to a new tube with a pipette. The cells were washed twice, using PBS and centrifugation at 500 ×* g* for 15 min and at 500 ×* g* for 10 min. From the mononuclear cells, positive separation with MACS CD4, CD8 and CD19 antibody MicroBeads (Miltenyi Biotec, Bergisch Gladbach, Germany) was performed using an OctoMACS magnetic separator (Miltenyi Biotec) following the manufacturers protocol. In addition to the CD4 +, CD8 + and CD19 + fractions, a CD4 − CD8 − CD19 − fraction was separated by using all of the above bead types to give a negative selection. Catalogue numbers for the used beads were 130-050-301 (CD19), 130-045-201 (CD8) and 130-045-101 (CD4). One separated CD8 + fraction was stimulated with a CD28 antibody. From the separated cell populations, DNA and RNA were extracted using the InviTrap Spin Universal RNA Mini Kit (Stratec Biomedical, Birkenfeld, Germany) according to manufacturer's instructions. The purities of the separated CD4 +, CD8 + and C19 + cells were tested in a subsample by flow cytometric analysis, in which the observed purities were > 90% for the CD4 + and CD8 + cells and > 70% for the CD19 + cells.

### 4.3 Custom gene panel

A custom designed gene panel referred to as Immunopanel-986 was utilized for the mutation screening step. The panel consists of 986 genes related to immunity and cancer, the bulk of which originated from the curated InnateDB database [32] with additional targets such as genes related to the JAK-STAT pathway compiled from different publications (for full gene list, see Table S1).

### 4.4 Screening phase library preparation and sequencing

DNA from the separated cell populations was fragmented using a Covaris S2 instrument (Covaris, Woburn, MA, USA) and then a sequencing library was prepared according to the NEBNext DNA Sample Prep Master Mix Set 1 (New England BioLabs, Ipswich, MA, USA) manual with some minor exceptions. Target enrichment for all exons of the genes included in the Immunopanel-986 gene panel was achieved with the Nimblegen SeqCap exon capture system (Roche NimbleGen, Madison, WI, USA). Library Purification was performed using Agencourt AMPure XP beads (Beckman Coulter, Brea, CA, USA). The target enriched library was sequenced with a HiSeq 2500 instrument (Illumina, San Diego, CA, USA) using paired end 101 bp reads.

### 4.5 Screening phase data analysis

Sequencing reads were mapped against the GRCh37 reference genome using BWA-MEM [33] after adapter trimming with the Trimmomatic [34] software. PCR duplicates were removed using Picard MarkDuplicates (http://broadinstitute.github.io/picard). VarScan2 [35] was used for somatic mutation calling. Each sequenced cell population acted in turn as the VarScan2 “tumor” (target) sample from which somatic mutations were called, and merged data from the other cell populations originating from the same patient acted as the VarScan2 “normal” (control) sample. The variant detection strategy of VarScan2 was augmented with custom logic to enable defining a cutoff for somatic variants of high enough confidence to be included in a validation step. The custom steps were as follows: first, all mismatches against the reference genome were tested using a binomial test with error rate estimated from the data to discard random non-systematic sequencing noise, Bonferroni corrected by the number of tests (size of target area ∗ 4, a test for each possible base). The variant sites were filtered for strand bias and against possible contamination from germline variants from other samples. Then the target (“tumor”) and control (“normal”) samples were compared using Fisher's exact test to show somatic status for sites that passed the first filters. The putative somatic variant list from all samples was then FDR corrected using the Benjamini and Hochberg method with FDR cutoff set to 0.05 to yield a final list of variants to feed into validation. Variants were annotated using the ANNOVAR [36] package. Execution of the analysis pipeline was performed using the Snakemake [37] software.

### 4.6 Validation and re-analysis of mutations by amplicon sequencing

PCR amplicons for each of the putative somatic variants identified in the screening phase were prepared following the “Illumina 16S Metagenomic Sequencing Library Preparation” [38] protocol, adjusted for targets other than the bacterial 16S gene. Briefly, all designed PCR primer pairs had their forward primer prepended with the sequence TCGTCGGCAGCGTCAGATGTGTATAAGAGACAG while the reverse primer was prepended with the sequence GTCTCGTGGGCTCGGAGATGTGTATAAGAGACAG. Primer oligos were ordered from Sigma Aldrich (St. Louis, MO, USA) and PCR was carried out using the KAPA HiFi polymerase (Kapa Biosystems, Wilmington, MA, USA). A second round of PCR utilizing the overhang sequences was then performed to incorporate full Illumina sequencing adapters using primers from the Nextera XT Index Kit (Illumina, San Diego, CA, USA). The amplicons were purified after both PCR steps using Agencourt AMPure XP beads (Beckman Coulter, Brea, CA, USA). The resulting library was sequenced using a MiSeq instrument (Illumina, San Diego, CA, USA) using 200 bp paired end reads. Amplicon sequencing was performed using DNA from the same cell populations in which a putative somatic variant was identified. In addition, amplicons of all targets were prepared using separate donor control DNA. Eight mutations that were validated in the first sequencing run were also tested in whole blood DNA using the same protocol. Sequencing reads were mapped against the GRCh37 reference genome using BWA-MEM [33] after adapter trimming with the Trimmomatic [34] software. VarScan2 [35] was used in validation mode to report somatic p-values (Fisher's exact test) at each of the genomic coordinates containing a variant to be validated. Data from patient cell populations served as the VarScan “tumor” input and data from the control DNA amplicons as the VarScan “normal” input. The data was also inspected directly from samtools [39] mpileup output. The somatic variants from Immunopanel HiSeq data were considered successfully verified if they met a p-value threshold of 0.001 in this data, Bonferroni corrected by the number of verifications performed and did not display strand bias. Variant deleteriousness predictions were performed using the CADD [20] algorithm, with a scaled score of at least 20 considered predictably deleterious.

### 4.7 FACS

An analysis of T cell receptor Vβ (beta chain variable region) repertoire was performed in CD4 + and CD8 + cells. The IO Test Beta Mark TCR Vβ Repertoire Kit (Beckman Coulter, Brea, CA, US) was used and flow cytometry was performed with a BD FACSAria II instrument (BD Biosciences, San Jose, CA, USA). A cell population was considered to contain a large clonal expansion if a Vβ type was seen in 10% or more of the cells and was present at over twice of the intensity of a population control frequency.

### 4.8 RNA sequencing

Gene expression in the target cell subpopulations (CD4 +, CD8 +, CD19 + and CD4 −/CD8 −/CD19 −) was analysed by RNA sequencing of two MS patients: MS17 and one patient (MS-0), who was not included to the somatic mutation analysis (female, no treatment, age 42). Additionally, CD8 + target cell basal RNA expression was analysed in two patients (MS-7, MS-8) and in one patient (MS-8) after stimulation with CD3 and CD28 antibodies [40]. Sequencing libraries were prepared using a strand-specific kit (New England BioLabs, Ipswich, MA, USA) and sequenced with the MiSeq instrument (Illumina, San Diego, CA, USA) with at least 25 million 300 bp read pairs produced per library. Reads were aligned using TopHat2 [41] and expression quantified using HTSeq-Count [42] and a custom script. The expression data is given in Table S4. Genes were considered expressed, when the cut-off 1 was reached in the FPKM value.

The following are the supplementary data related to this article.

Supplementary Fig. S1. Flow cytometry Vbeta analysis of the patient MS-8. Frozen live peripheral blood mononuclear cells were thawed and labeled with anti-CD3, anti-CD8, anti-CD4 and monoclonal Vbeta antibodies. Vbeta antibodies are conjugated either with FITC (x-axis), PE (y-axis), or both PE and FITC (a double positive population). CD4 + and CD8 + lymphocytes are analysed separately. The 24 Vbeta populations analysed in 8 tubes (A-H) are shown. Supplementary Fig. S1

Supplementary Fig. S2. Vbetaquantification of all 20 patients. 10% bar added to those patients, who had a large (> 10%) clone in CD8 + cells. Supplementary Fig. S2

Supplementary Table S1. Genes included in the screening phase panel “Immunopanel-986” (exon capture). Supplementary Table S1

Supplementary Table S2. Genomic coordinates (GRCh37) for successfully validated somatic mutations and the level of supporting evidence. Supplementary Table S2

Supplementary Table S3. Read count data for whole blood somatic mutation amplicons. Supplementary Table S3

Supplementary Table S4. RNA-Sequencing FPKM expression values for the 27 genes in which somatic mutations were detected.
Genes were considered expressed, when the FPKM value > 1 was reached, The values serve as a reference of gene expression in specific cell populations separated using our protocol. As such these values do not represent the expression status of the mutated allele. All target cell populations were analysed in two patients (MS17 and MS0). CD8 + basal expression was analysed in two additional patients (MS-7 and MS-8) and CD8 + expression after stimulation in one patient (MS-8). Supplementary Table S4

Supplementary Table S5. Presence of large clones in CD8 + patient cell populations. Supplementary Table S5

Supplementary Table S6. Somatic mutation persistence over time. Supplementary Table S6

Supplementary Table S7. Somatic mutation persistence read count data (amplicon sequencing). Supplementary Table S7

Supplementary Table S8. RNA-Seq FPKM expression values for genes in which somatic mutations were detected in the study. Supplementary Table S8

## Figures and Tables

**Table 1:** Baseline characteristics of the patients.

Patient	Diagnosis	Immunological medication	Age	Sex	Disease duration (years)	Separated cell populations^a^
MS-1	Multiple sclerosis	Natalizumab	35	M	2	CD4 +, CD8 +, CD19 +
MS-2	Multiple sclerosis	None^b^	55	F	9	CD4 +, CD8 +, CD19 +, CD4 − CD8 − CD19 −
MS-3	Multiple sclerosis	Natalizumab	38	M	4	CD4 +, CD8 +, CD19 +, CD4 − CD8 − CD19 −
MS-4	Multiple sclerosis	Natalizumab	32	F	3	CD4 +, CD8 +, CD19 +, CD4 − CD8 − CD19 −
MG-5	Myasthenia gravis	None	67	F	0.5	CD4 +, CD8 +, CD19 +, CD4 − CD8 − CD19 −
SP-6	Stiff-person syndrome (GAD antibody positive), SLE	Rituximab, mycophenolate mofetil, gammaglobulin	36	F	13	CD4 +, CD8 +, CD4 − CD8 − CD19 −
MS-7	Multiple sclerosis	Glatiramer acetate	57	F	13	CD4 +, CD8 +, CD19 +, CD4 − CD8 − CD19 −
MS-8	Multiple sclerosis	Azathioprine	59	F	35	CD4 +, CD8 +, CD19 +, CD4 − CD8 − CD19 −, CD28 ab stimulated CD8 +
NL-9	Narcolepsy (type 2 non-cataplectic)	None	24	M	3	CD4 +, CD8 +, CD19 +, CD4 − CD8 − CD19 −
MS-10	Multiple sclerosis	Interferon beta	25	F	2	CD4 +, CD8 +, CD19 +, CD4 − CD8 − CD19 −
MS-11	Multiple sclerosis + Type 1 diabetes	None	40	F	6	CD4 +, CD8 +, CD19 +, CD4 − CD8 − CD19 −
MS-12	Multiple sclerosis	Interferon beta	46	F	0.5	CD4 +, CD8 +, CD19 +, CD4 − CD8 − CD19 −
MS-14	Multiple sclerosis	None	48	F	7	CD4 +, CD8 +, CD19 +
NL-16	Narcolepsy-cataplexy (type-2)	None	22	M	3	CD4 +, CD8 +, CD19 +
MS-17	Multiple sclerosis	Natalizumab	58	F	15	CD4 +, CD8 +, CD19 +
MS-19	Multiple sclerosis	Glatiramer acetate	35	F	16	CD4 +, CD8 +, CD19 +
MS-21	Multiple sclerosis	Interferon beta	54	F	10	CD4 +, CD8 +, CD19 +
MS-22	Multiple sclerosis	None	43	F	0.5	CD4, CD8 +, CD19 +
MS-23	Multiple sclerosis	None	29	F	0.5	CD4 +, CD8 +, CD19 +
MS-24	Multiple sclerosis	Interferon beta	22	F	1.5	CD4 +, CD8 +, CD19 +

a Cell populations with adequate amount of DNA for sequencing are shown.

b Previous treatment with interferon-beta, glatiramer acetate, natalizumab, mitoxanthrone, and azathioprine.

**Table 2:** Number of somatic mutations discovered per patient.

N mutations	Patients
4	MS-8, MS-19, MS-21
3	MS-2
2	MS-1, MS-3, NL-9, MS-12
1	MG-5, MS-14, MS-22, MS-23
0	MS-4, SP-6, MS-7, MS-10, MS-11, NL-16, MS-17, MS-24

**Table 3:** Successfully validated somatic mutations in decreasing order of allelic fraction.

Allelic fraction (validation)	Allelic fraction (discovery)	Sample	Gene	AA change	CADD score
4.63%	4.71%	MS-12-CD8 +	CD1C	R89C	10.0
4.03%	2.71%	MS-8-CD19 +	TRAF2	409–410 LF/L^b^	21.6^c^
2.88%	2.29%	MS-19-CD8 +	CD46	A250T^b^	23.4^c^
2.81%	2.70%	MS-21-CD8 +	RBM6	P24S^b^	25.3^c^
2.71%	2.70%	MS-2-CD8 +	A2ML1^a^	R1001W	32.0^c^
2.28%	1.62%	MS-1-CD8 +	IKZF3	155–156 FT/S^b^	22.9^c^
2.23%	2.14%	MS-2-CD8 +	BTK	Splicing^b^	24.2^c^
1.58%	1.69%	MS-19-CD8 +	PTPMT1	I165V^b^	17.0
1.58%	1.29%	MG-5-others	CD56/NCAM1	G417R^b^	34.0^c^
1.36%	0.70%	MS-19-CD8 +	ITGA2^a^	Q581K	16.4
1.22%	0.86%	MS-21-CD8 +	CD56/NCAM1	R358X^b^	18.2
1.21%	1.68%	MS-8-CD8 +	RPA1	L394P^b^	25.5^c^
0.98%	1.32%	MS-8-CD8 +	KIR3DL2	D392Y	23.4^c^
0.95%	1.15%	MS-21-CD8 +	RORA	R533Q	23.0^c^
0.83%	1.23%	MS-19-CD8 +	PSG1^a^	S23T^b^	12.4
0.83%	1.04%	MS-3-CD8 +	HMMR	E405K^b^	26.5^c^
0.80%	1.31%	NL-9-CD8 +	C6^a^	A298P^b^	23.1^c^
0.68%	0.80%	NL-9-CD8 +	CLIP2	R932H	34.0^c^
0.59%	1.07%	MS-1-CD8 +	MBL2^a^	A37S	8.9
0.46%	0.74%	MS-2-CD4 +	CD180	D526Y^b^	24.4^c^
0.45%	0.70%	MS-21-CD8 +	STAT3	D661Y	34.0^c^
0.40%	0.74%	MS-22-CD8 +	INSR^a^	A119V^b^	31.0^c^
0.35%	0.46%	MS-14-CD8 +	ITGB3	F229L^b^	29.7^c^
0.32%	0.53%	MS-8-CD8 +	TLR7^a^	N275K^b^	0.009
0.23%	0.60%	MS-3-CD8 +	MAPK10^a^	S316F^b^	22.2^c^
0.19%	1.34%	MS-12-others	ATM	L2519V^b^	26.3^c^
0.19%	0.56%	MS-23-CD8 +	CFH	V407L^b^	0.001

The allelic fraction of a somatic mutation is calculated as the percentage of sequencing reads that contain the variant base. Genomic coordinates are shown in Table S2. Sample code consists of the patient number and a cell population specifier where “others” denotes the CD4 − CD8 − CD19 − population.

The allelic fraction of a somatic mutation is calculated as the percentage of sequencing reads that contain the variant base. Genomic coordinates are shown in Table S2. Sample code consists of the patient number and a cell population specifier where “others” denotes the CD4 − CD8 − CD19 − population.

a Gene not expressed in the target cell population type based on RNA sequencing data.

b Indicates a novel mutation.

c CADD scores of 20 or more can be considered predictably deleterious.

**Table 4:** Number of validated somatic mutations per cell population.

Cell population type	Mutations detected
CD4 +	1
CD8 +	23
CD19 +	1
CD4 − CD8 − CD19 −	2

## References

[1] Watson I.R., Takahashi K., Futreal P.A., Chin L. (2013). Emerging patterns of somatic mutations in cancer. Nat. Rev. Genet..

[2] Busque L., Patel J.P., Figueroa M.E., Vasanthakumar A., Provost S., Hamilou Z., Mollica L., Li J., Viale A., Heguy A. (2012). Recurrent somatic TET2 mutations in normal elderly individuals with clonal hematopoiesis. Nat. Genet..

[3] Genovese G., Kähler A.K., Handsaker R.E., Lindberg J., Rose S.A., Bakhoum S.F., Chambert K., Mick E., Neale B.M., Fromer M. (2014). Clonal hematopoiesis and blood-cancer risk inferred from blood DNA sequence. N. Engl. J. Med..

[4] Jaiswal S., Fontanillas P., Flannick J., Manning A., Grauman P.V., Mar B.G., Lindsley R.C., Mermel C.H., Burtt N., Chavez A. (2014). Age-related clonal hematopoiesis associated with adverse outcomes. N. Engl. J. Med..

[5] Xie M., Lu C., Wang J., McLellan M.D., Johnson K.J., Wendl M.C., McMichael J.F., Schmidt H.K., Yellapantula V., Miller C.A. (2014). Age-related mutations associated with clonal hematopoietic expansion and malignancies. Nat. Med..

[6] Holzelova E., Vonarbourg C., Stolzenberg M., Arkwright P.D., Selz F., Prieur A., Blanche S., Bartunkova J., Vilmer E., Fischer A. (2004). Autoimmune lymphoproliferative syndrome with somatic Fas mutations. N. Engl. J. Med..

[7] Niemela J.E., Lu L., Fleisher T.A., Davis J., Caminha I., Natter M., Beer L.A., Dowdell K.C., Pittaluga S., Raffeld M., Rao V.K., Oliveira J.B. (2011). Somatic KRAS mutations associated with a human nonmalignant syndrome of autoimmunity and abnormal leukocyte homeostasis. Blood.

[8] Koskela H.L., Eldfors S., Ellonen P., van Adrichem A.J., Kuusanmäki H., Andersson E.I., Lagström S., Clemente M.J., Olson T., Jalkanen S.E. (2012). Somatic STAT3 mutations in large granular lymphocytic leukemia. N. Engl. J. Med..

[9] International Multiple Sclerosis Genetics Consortium (2011). Wellcome Trust Case Control Consortium 2. Genetic risk and a primary role for cell-mediated immune mechanisms in Multiple Sclerosis. Nature.

[10] International Multiple Sclerosis Genetics Consortium (2013). Analysis of immune-related loci identifies 48 new susceptibility variants for multiple sclerosis. Nat. Genet..

[11] Wingerchuk D.M., Weinshenker B.G. (2016). Disease modifying therapies for relapsing multiple sclerosis. BMJ.

[12] Ascherio A., Munger K.L. (2015). EBV and autoimmunity. Curr. Top. Microbiol. Immunol..

[13] Allegretta M., Nicklas J.A., Sriram S., Albertini R.J. (1990). T cells responsive to myelin basic protein in patients with multiple sclerosis. Science.

[14] Albertini R.J., Castle K.L., Borcherding W.R. (1982). T-cell cloning to detect the mutant 6-thioguanine-resistant lymphocytes present in human peripheral blood. Proc. Natl. Acad. Sci. U. S. A..

[15] Sriram S. (1994). Longitudinal study of frequency of HPRT mutant T cells in patients with multiple sclerosis. Neurology.

[16] Kemppinen A.K., Baker A., Liao W., Fiddes B., Jones J., Compston A., Ban M., Sawcer S. (2014). Exome sequencing in single cells from the cerebrospinal fluid in multiple sclerosis. Mult. Scler..

[17] Forbes S.A., Beare D., Gunasekaran P., Leung K., Bindal N., Boutselakis H., Ding M., Bamford S., Cole C., Ward S., Kok C.Y., Jia M., De T., Teague J.W., Stratton M.R., McDermott U., Campbell P.J. (2015). COSMIC: exploring the world's knowledge of somatic mutations in human cancer. Nucleic Acids Res..

[18] Sherry S.T., Ward M.H., Kholodov M., Baker J., Phan L., Smigielski E.M., Sirotkin K. (2001). dbSNP: the NCBI database of genetic variation. Nucleic Acids Res..

[19] Lek M., Karczewski K., Minikel E., Samocha K., Banks E., Fennell T., O'Donnell-Luria A., Ware J., Hill A., Cummings B. (2015). Analysis of protein-coding genetic variation in 60,706 humans. bioRxiv.

[20] Kircher M., Witten D.M., Jain P., O'Roak B.J., Cooper G.M., Shendure J. (2014). A general framework for estimating the relative pathogenicity of human genetic variants. Nat. Genet..

[21] Rajala H.L., Olson T., Clemente M.J., Lagstrom S., Ellonen P., Lundan T., Hamm D.E., Zaman S.A., Lopez Marti J.M., Andersson E.I., Jerez A., Porkka K., Maciejewski J.P., Loughran T.P., Mustjoki S. (2015). The analysis of clonal diversity and therapy responses using STAT3 mutations as a molecular marker in large granular lymphocytic leukemia. Haematologica.

[22] Haring J.S., Pewe L.L., Perlman S. (2002). Bystander CD8 T cell-mediated demyelination after viral infection of the central nervous system. J. Immunol..

[23] Salou M., Garcia A., Michel L., Gainche-Salmon A., Loussouarn D., Nicol B., Guillot F., Hulin P., Nedellec S., Baron D. (2015). Expanded CD8 T-cell sharing between periphery and CNS in multiple sclerosis. Ann. Clin. Transl. Neurol..

[24] Schmitt M.W., Kennedy S.R., Salk J.J., Fox E.J., Hiatt J.B., Loeb L.A. (2012). Detection of ultra-rare mutations by next-generation sequencing. Proc. Natl. Acad. Sci. U. S. A..

[25] Qi Q., Liu Y., Cheng Y., Glanville J., Zhang D., Lee J.Y., Olshen R.A., Weyand C.M., Boyd S.D., Goronzy J.J. (2014). Diversity and clonal selection in the human T-cell repertoire. Proc. Natl. Acad. Sci. U. S. A..

[26] Maini M.K., Casorati G., Dellabona P., Wack A., Beverley P.C. (1999). T-cell clonality in immune responses. Immunol. Today.

[27] Callan M.F., Steven N., Krausa P., Wilson J.D., Moss P.A., Gillespie G.M., Bell J.I., Rickinson A.B., McMichael A.J. (1996). Large clonal expansions of CD8 T cells in acute infectious mononucleosis. Nat. Med..

[28] Huseby E.S., Liggitt D., Brabb T., Schnabel B., Ohlen C., Goverman J. (2001). A pathogenic role for myelin-specific CD8(+) T cells in a model for multiple sclerosis. J. Exp. Med..

[29] Babbe H., Roers A., Waisman A., Lassmann H., Goebels N., Hohlfeld R., Friese M., Schroder R., Deckert M., Schmidt S., Ravid R., Rajewsky K. (2000). Clonal expansions of CD8(+) T cells dominate the T cell infiltrate in active multiple sclerosis lesions as shown by micromanipulation and single cell polymerase chain reaction. J. Exp. Med..

[30] Friese M.A., Jakobsen K.B., Friis L., Etzensperger R., Craner M.J., McMahon R.M., Jensen L.T., Huygelen V., Jones E.Y., Bell J.I. (2008). Opposing effects of HLA class I molecules in tuning autoreactive CD8 T cells in multiple sclerosis. Nat. Med..

[31] Jakkula E., Leppä V., Sulonen A., Varilo T., Kallio S., Kemppinen A., Purcell S., Koivisto K., Tienari P., Sumelahti M. (2010). Genome-wide association study in a high-risk isolate for multiple sclerosis reveals associated variants in STAT3 gene. Am. J. Hum. Genet..

[32] Breuer K., Foroushani A.K., Laird M.R., Chen C., Sribnaia A., Lo R., Winsor G.L., Hancock R.E., Brinkman F.S., Lynn D.J. (2013). InnateDB: systems biology of innate immunity and beyond—recent updates and continuing curation. Nucleic Acids Res..

[33] Li H. (2013). Aligning sequence reads, clone sequences and assembly contigs with BWA-MEM. arXiv preprint arXiv:1303.3997.

[34] Bolger A.M., Lohse M., Usadel B. (2014). Trimmomatic: a flexible trimmer for Illumina sequence data. Bioinformatics.

[35] Koboldt D.C., Zhang Q., Larson D.E., Shen D., McLellan M.D., Lin L., Miller C.A., Mardis E.R., Ding L., Wilson R.K. (2012). VarScan 2: somatic mutation and copy number alteration discovery in cancer by exome sequencing. Genome Res..

[36] Wang K., Li M., Hakonarson H. (2010). ANNOVAR: functional annotation of genetic variants from high-throughput sequencing data. Nucleic Acids Res..

[37] Koster J., Rahmann S. (2012). Snakemake–a scalable bioinformatics workflow engine. Bioinformatics.

[38] Illumina. 16S Metagenomic Sequencing Library Preparation, http://support.illumina.com/documents/documentation/chemistry_documentation/16s/16s-metagenomic-library-prep-guide-15044223-b.pdf.

[39] Li H., Handsaker B., Wysoker A., Fennell T., Ruan J., Homer N., Marth G., Abecasis G., Durbin R. (2009). 1000 genome project data processing subgroup. The sequence alignment/map format and SAMtools. Bioinformatics.

[40] Honkanen J., Nieminen J.K., Gao R., Luopajarvi K., Salo H.M., Ilonen J., Knip M., Otonkoski T., Vaarala O. (2010). IL-17 immunity in human type 1 diabetes. J. Immunol..

[41] Kim D., Pertea G., Trapnell C., Pimentel H., Kelley R., Salzberg S.L. (2013). TopHat2: accurate alignment of transcriptomes in the presence of insertions, deletions and gene fusions. Genome Biol..

[42] Anders S., Pyl P.T., Huber W. (2015). HTSeq–a python framework to work with high-throughput sequencing data. Bioinformatics.

