# Supplementary material for: A novel class of somatic mutations in blood detected preferentially in CD8 + cells
Source: Clin Immunol. 2017 Feb;175:75–81. doi: 10.1016/j.clim.2016.11.018 (PMC5341785; doi:10.1016/j.clim.2016.11.018)
Supplement: Supplementary Fig. S1. — Flow cytometry Vbeta analysis of the patient MS-8. Frozen live peripheral blood mononuclear cells were thawed and labeled with anti-CD3, anti-CD8, anti-CD4 and monoclonal Vbeta antibodies. Vbeta antibodies are conjugated either with FITC (x-axis), PE (y-axis), or both PE and FITC (a double positive population). CD4 + and CD8 + lymphocytes are analysed separately. The 24 Vbeta populations analysed in 8 tubes (A-H) are shown. [file mmc1.pdf]

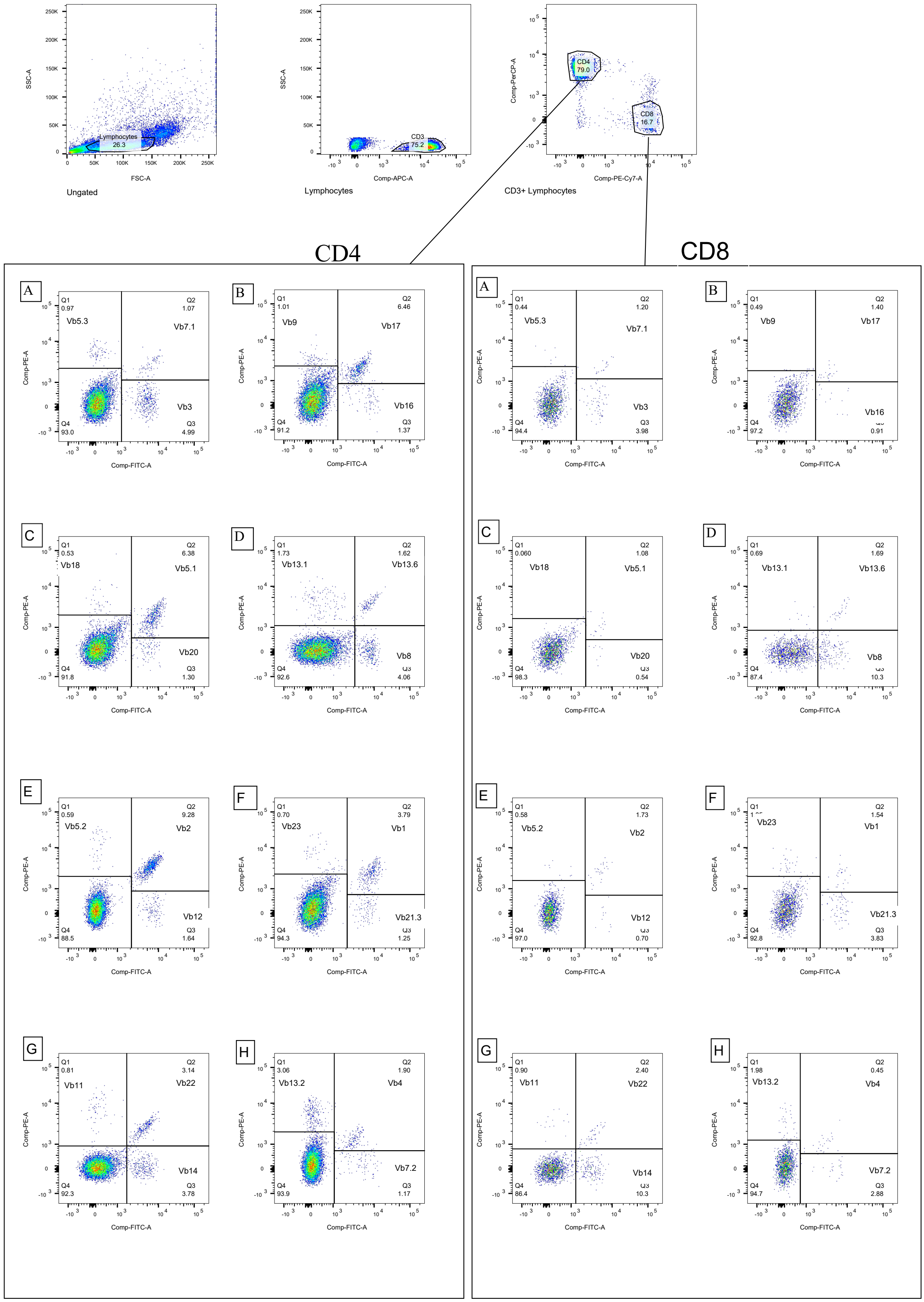


**Supplementary Figure 1. Flow cytometry Vbeta analysis of the patient MS-8.** Frozen live peripheral blood mononuclear cells were thawed and labeled with anti-CD3, anti-CD8, anti-CD4 and monoclonal Vbeta antibodies. Vbeta antibodies are conjugated either with FITC (x-axis), PE (y-axis), or both PE and FITC (a double positive population). CD4+ and CD8+ lymphocytes are analyzed separately. The 24 Vbeta populations analyzed in 8 tubes (A-H) are shown.
