# Supplementary material for: A novel class of somatic mutations in blood detected preferentially in CD8 + cells
Source: Clin Immunol. 2017 Feb;175:75–81. doi: 10.1016/j.clim.2016.11.018 (PMC5341785; doi:10.1016/j.clim.2016.11.018)
Supplement: Supplementary Fig. S2. — Vbetaquantification of all 20 patients. 10% bar added to those patients, who had a large (> 10%) clone in CD8 + cells. [file mmc2.pdf]

Supplementary Figure 2. Vbeta quantification of all 20 patients. 10 percent bar added to those patients, who had a large (>10%) clone in CD8+ cells.

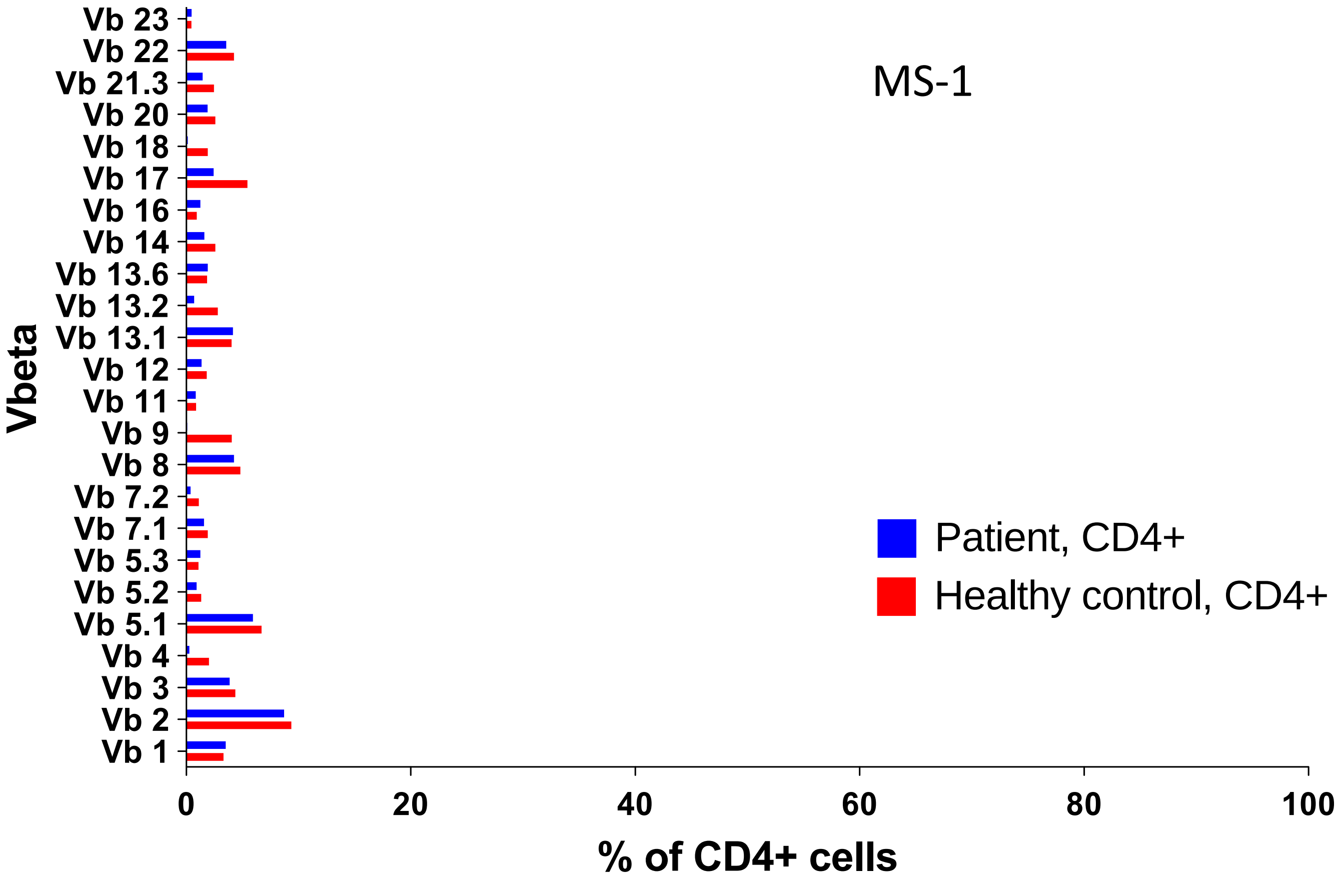

MS-1
Vbeta
Vb 23
Vb 22
Vb 21.3
Vb 20
Vb 18
Vb 17
Vb 16
Vb 14
Vb 13.6
Vb 13.2
Vb 13.1
Vb 12
Vb 11
Vb 9
Vb 8
Vb 7.2
Vb 7.1
Vb 5.3
Vb 5.2
Vb 5.1
Vb 4
Vb 3
Vb 2
Vb 1
0
20
40
60
80
100
% of CD4+ cells
Patient, CD4+
Healthy control, CD4+

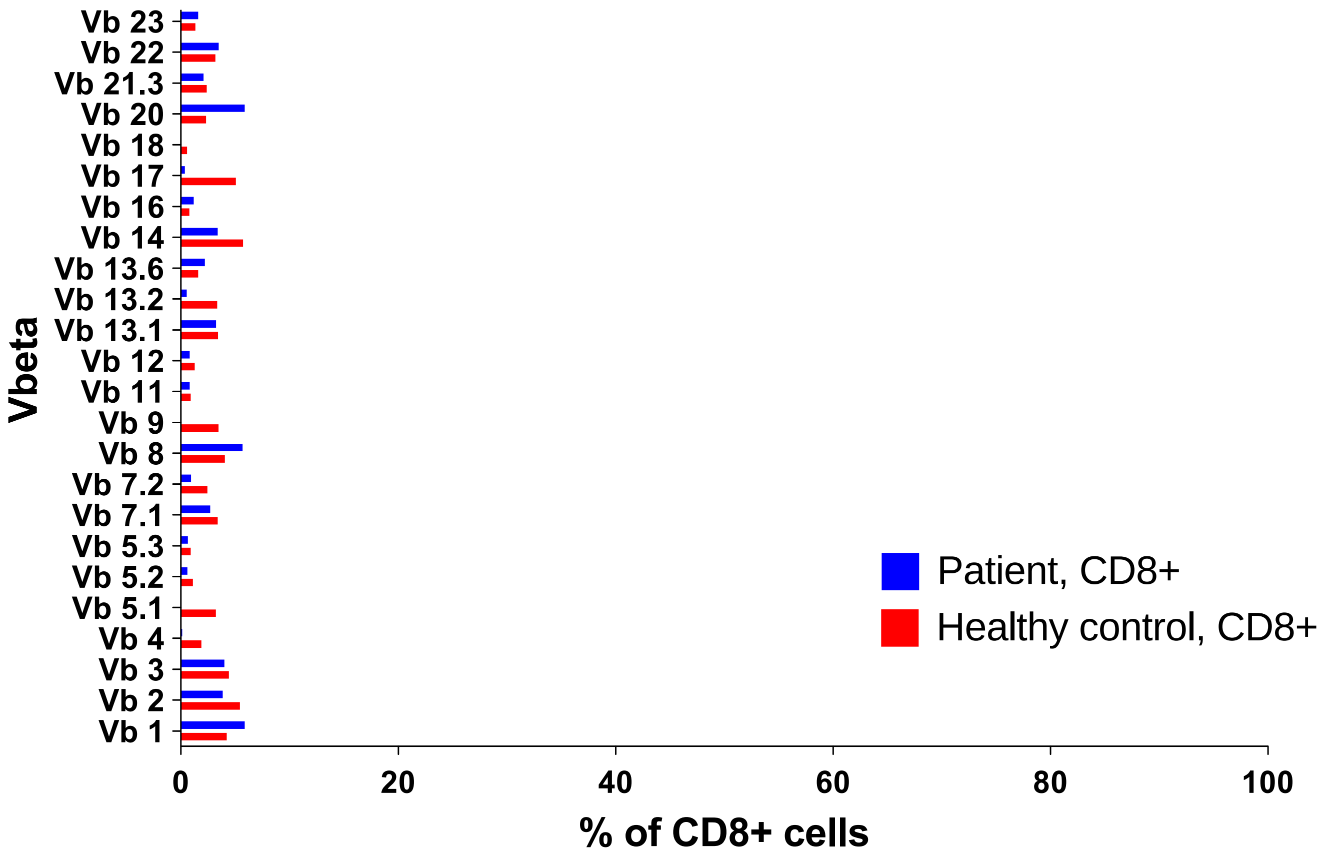

Vbeta
Vb 23
Vb 22
Vb 21.3
Vb 20
Vb 18
Vb 17
Vb 16
Vb 14
Vb 13.6
Vb 13.2
Vb 13.1
Vb 12
Vb 11
Vb 9
Vb 8
Vb 7.2
Vb 7.1
Vb 5.3
Vb 5.2
Vb 5.1
Vb 4
Vb 3
Vb 2
Vb 1
0
20
40
60
80
100
% of CD8+ cells
Patient, CD8+
Healthy control, CD8+

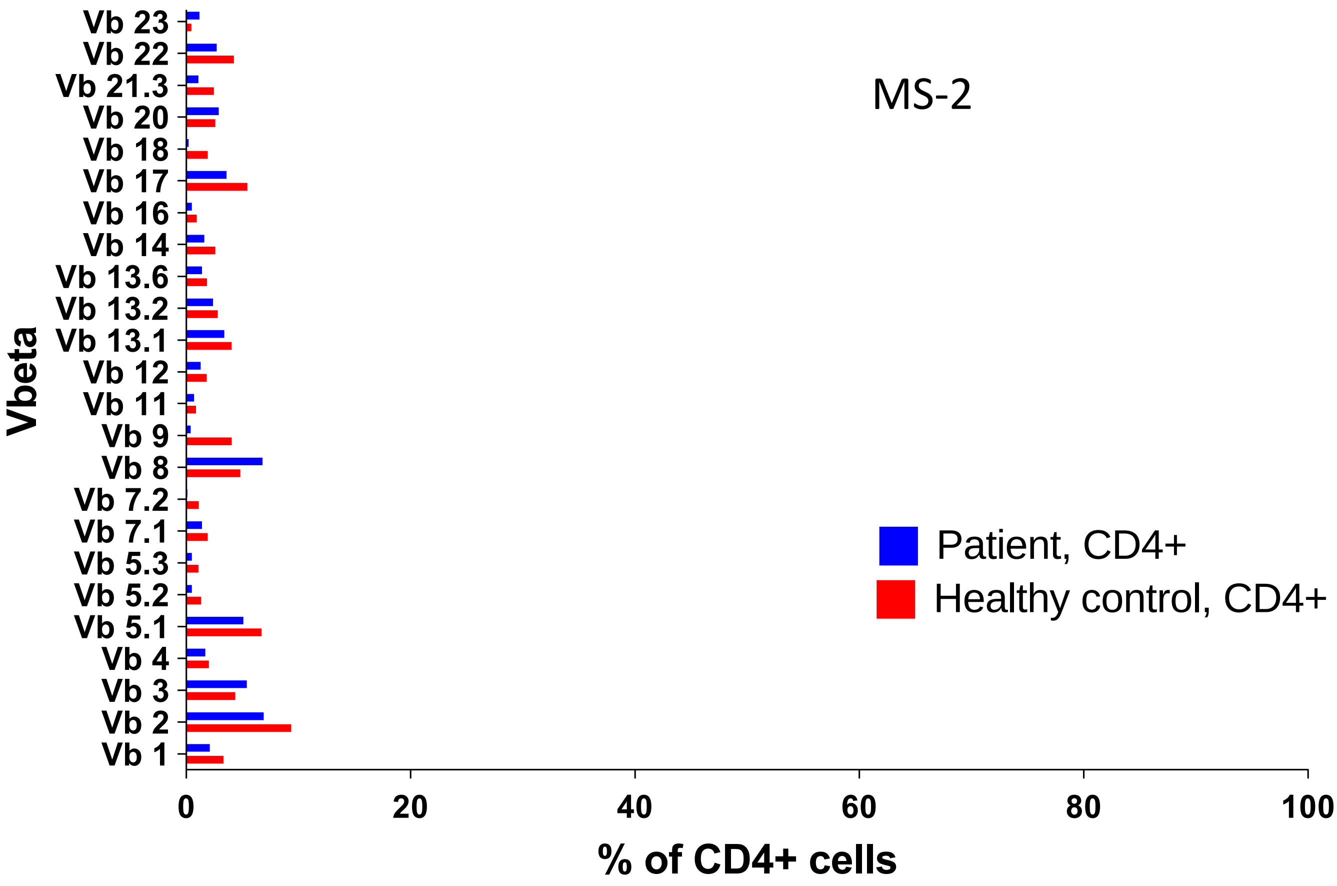

MS-2
Vbeta
Vb 23
Vb 22
Vb 21.3
Vb 20
Vb 18
Vb 17
Vb 16
Vb 14
Vb 13.6
Vb 13.2
Vb 13.1
Vb 12
Vb 11
Vb 9
Vb 8
Vb 7.2
Vb 7.1
Vb 5.3
Vb 5.2
Vb 5.1
Vb 4
Vb 3
Vb 2
Vb 1
0
20
40
60
80
100
% of CD4+ cells
Patient, CD4+
Healthy control, CD4+

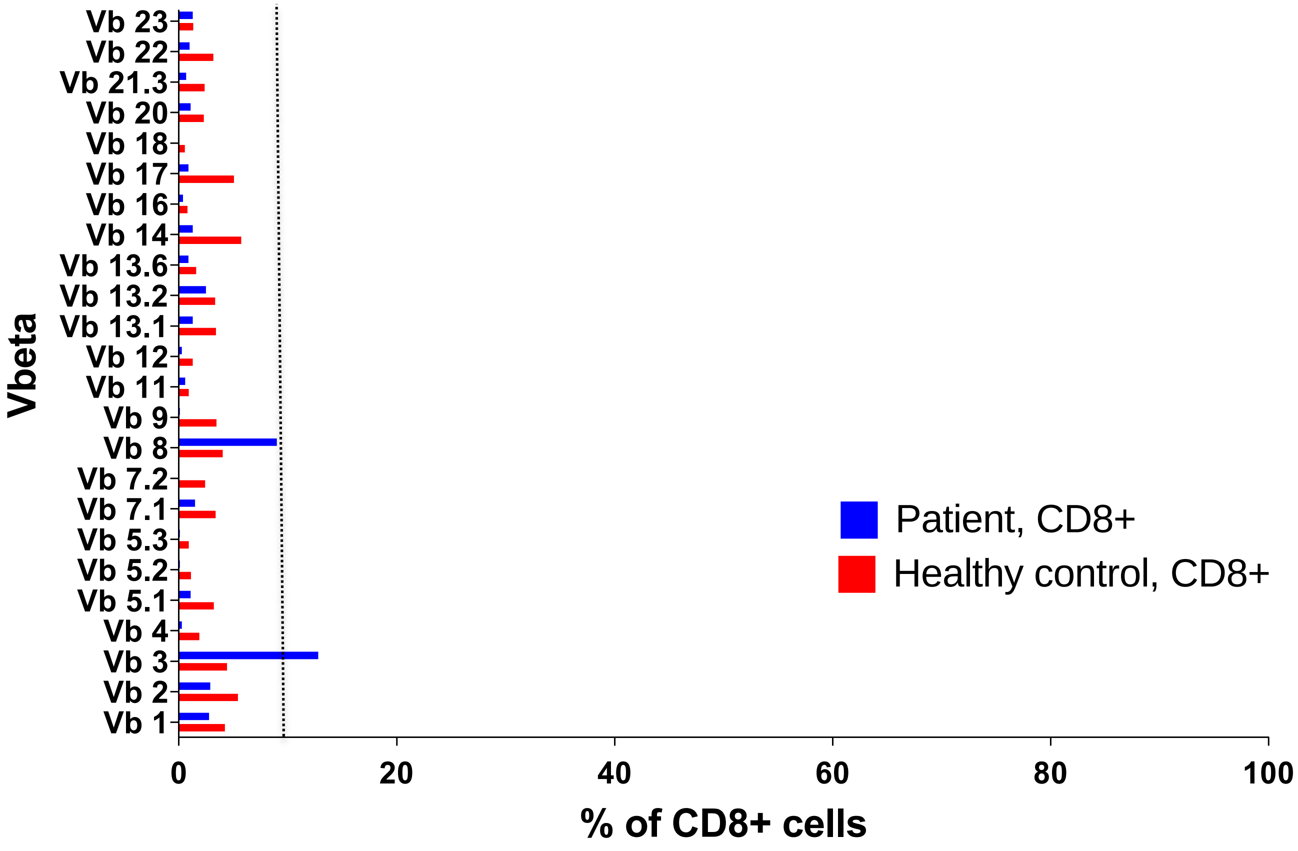

Vbeta
Vb 23
Vb 22
Vb 21.3
Vb 20
Vb 18
Vb 17
Vb 16
Vb 14
Vb 13.6
Vb 13.2
Vb 13.1
Vb 12
Vb 11
Vb 9
Vb 8
Vb 7.2
Vb 7.1
Vb 5.3
Vb 5.2
Vb 5.1
Vb 4
Vb 3
Vb 2
Vb 1
0
20
40
60
80
100
% of CD8+ cells
Patient, CD8+
Healthy control, CD8+

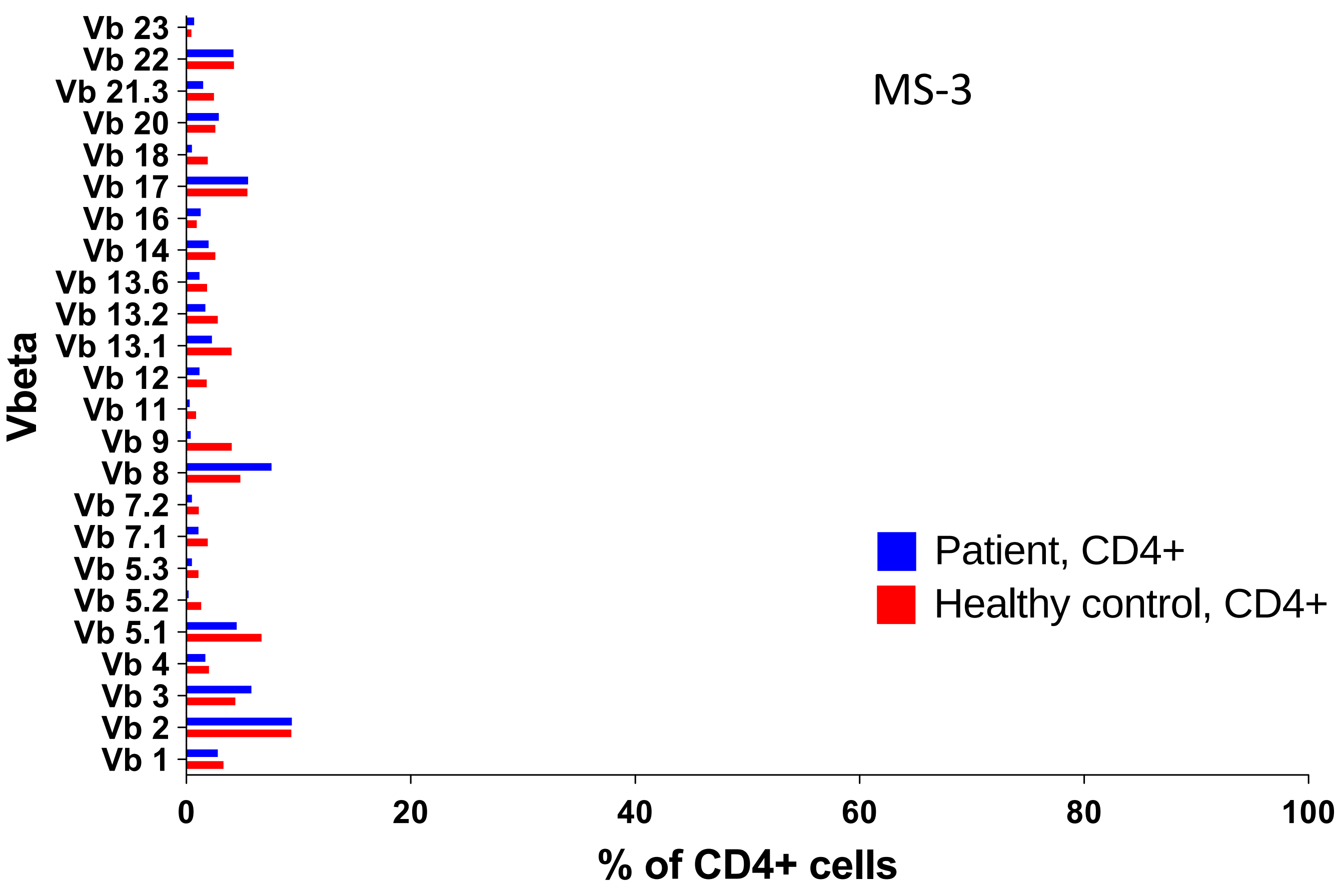
MS-3
Vbeta
Vb 23
Vb 22
Vb 21.3
Vb 20
Vb 18
Vb 17
Vb 16
Vb 14
Vb 13.6
Vb 13.2
Vb 13.1
Vb 12
Vb 11
Vb 9
Vb 8
Vb 7.2
Vb 7.1
Vb 5.3
Vb 5.2
Vb 5.1
Vb 4
Vb 3
Vb 2
Vb 1
0
20
40
60
80
100
% of CD4+ cells
Patient, CD4+
Healthy control, CD4+

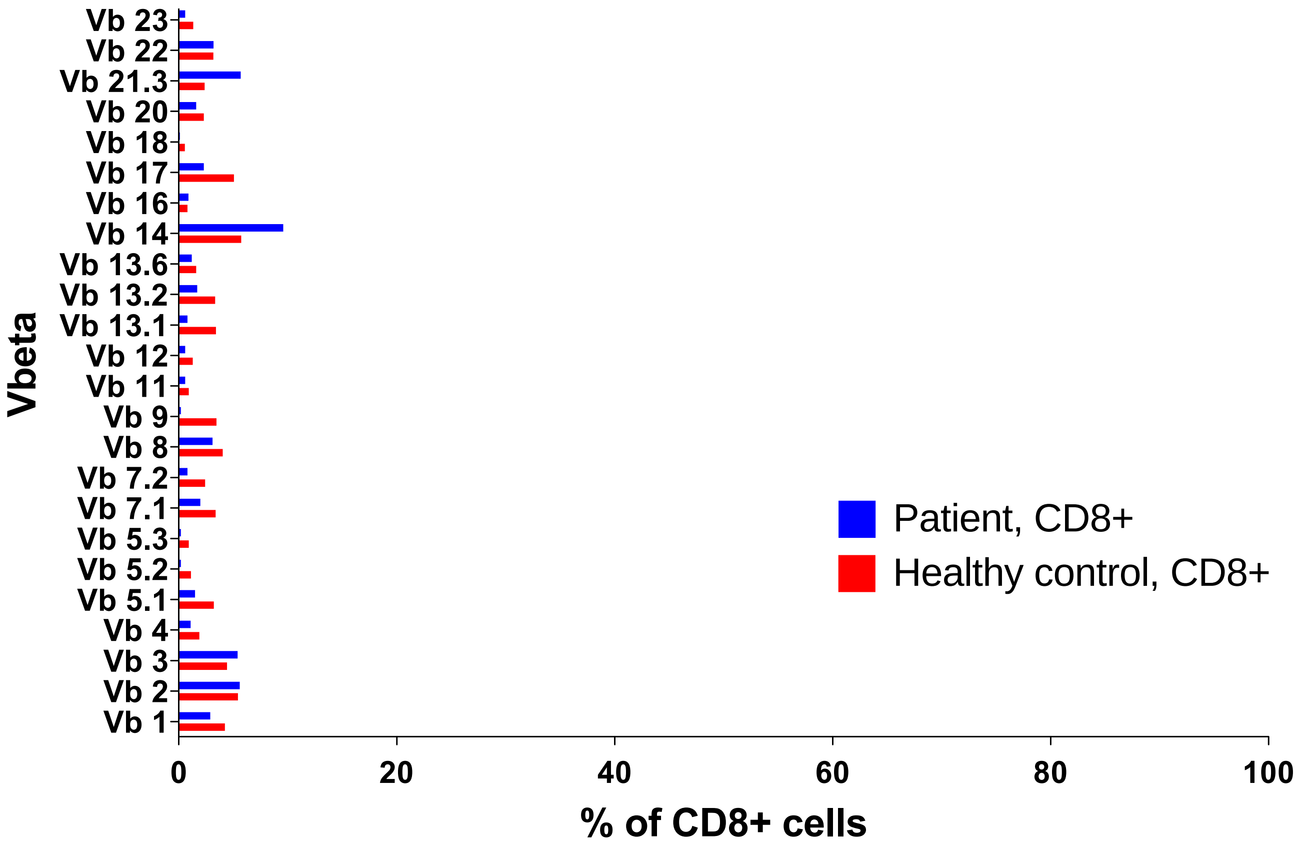
Vbeta
Vb 23
Vb 22
Vb 21.3
Vb 20
Vb 18
Vb 17
Vb 16
Vb 14
Vb 13.6
Vb 13.2
Vb 13.1
Vb 12
Vb 11
Vb 9
Vb 8
Vb 7.2
Vb 7.1
Vb 5.3
Vb 5.2
Vb 5.1
Vb 4
Vb 3
Vb 2
Vb 1
0
20
40
60
80
100
% of CD8+ cells
Patient, CD8+
Healthy control, CD8+

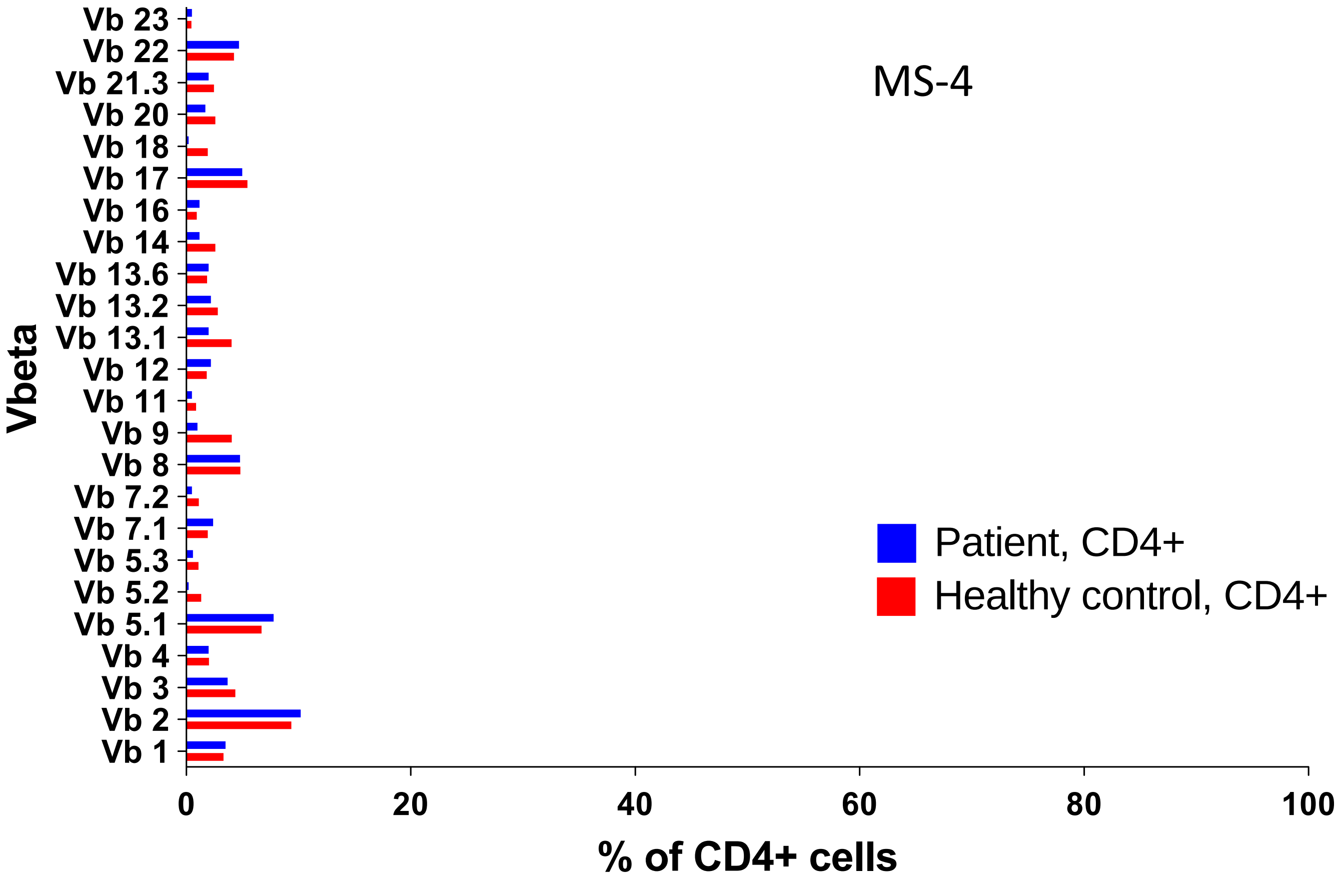
MS-4
Vbeta
Vb 23
Vb 22
Vb 21.3
Vb 20
Vb 18
Vb 17
Vb 16
Vb 14
Vb 13.6
Vb 13.2
Vb 13.1
Vb 12
Vb 11
Vb 9
Vb 8
Vb 7.2
Vb 7.1
Vb 5.3
Vb 5.2
Vb 5.1
Vb 4
Vb 3
Vb 2
Vb 1
0
20
40
60
80
100
% of CD4+ cells
Patient, CD4+
Healthy control, CD4+

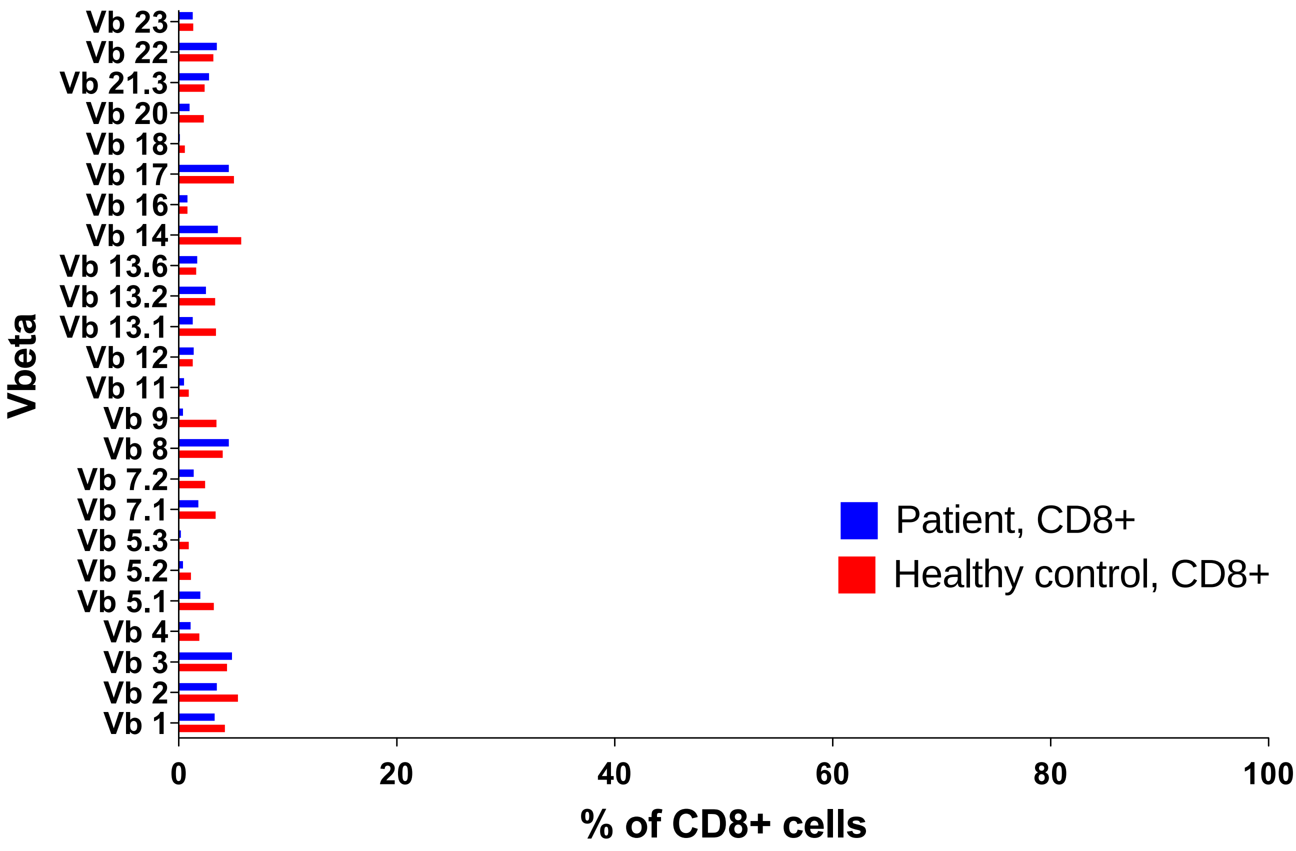
Vbeta
Vb 23
Vb 22
Vb 21.3
Vb 20
Vb 18
Vb 17
Vb 16
Vb 14
Vb 13.6
Vb 13.2
Vb 13.1
Vb 12
Vb 11
Vb 9
Vb 8
Vb 7.2
Vb 7.1
Vb 5.3
Vb 5.2
Vb 5.1
Vb 4
Vb 3
Vb 2
Vb 1
0
20
40
60
80
100
% of CD8+ cells
Patient, CD8+
Healthy control, CD8+

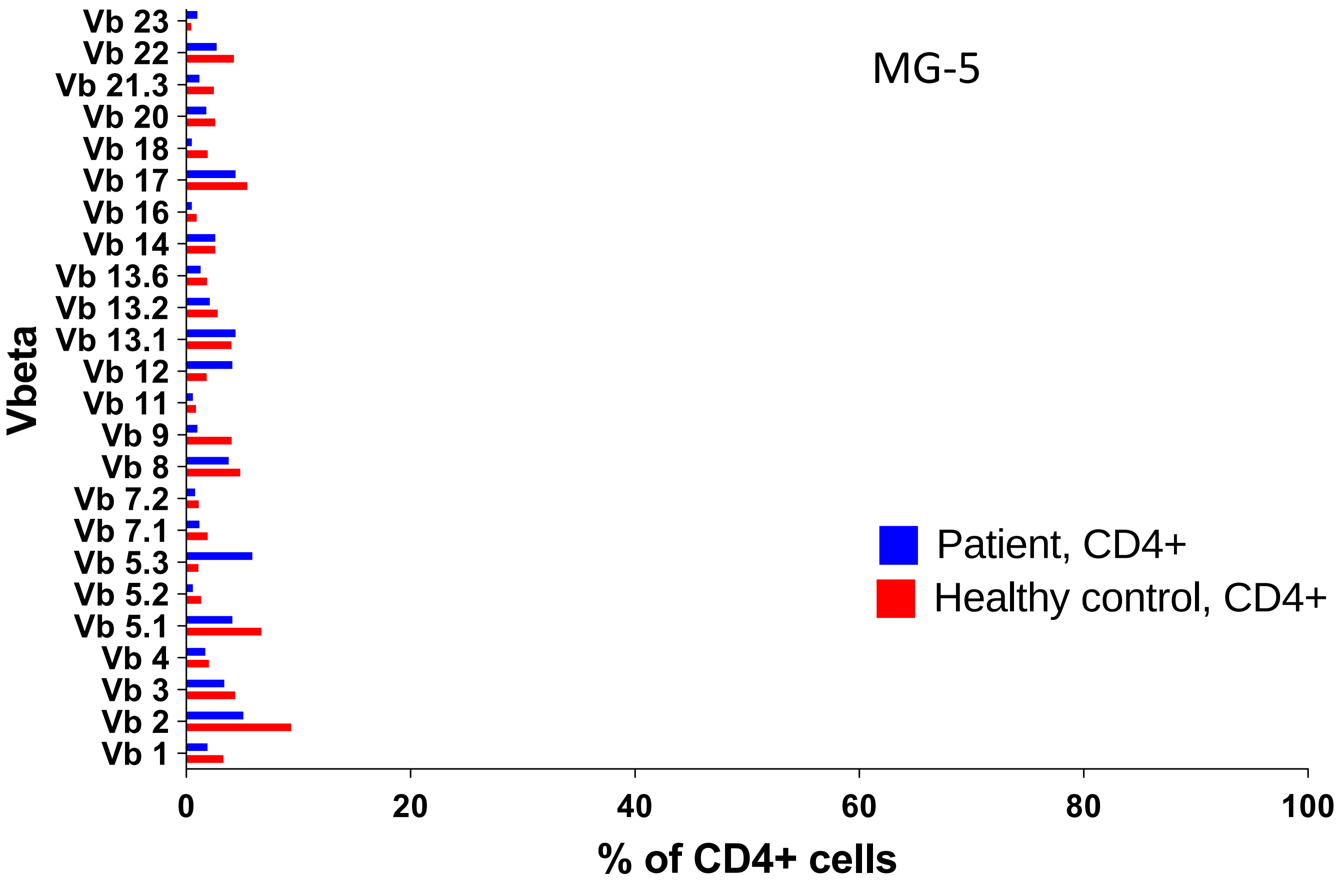

MG-5
Vbeta
Vb 23
Vb 22
Vb 21.3
Vb 20
Vb 18
Vb 17
Vb 16
Vb 14
Vb 13.6
Vb 13.2
Vb 13.1
Vb 12
Vb 11
Vb 9
Vb 8
Vb 7.2
Vb 7.1
Vb 5.3
Vb 5.2
Vb 5.1
Vb 4
Vb 3
Vb 2
Vb 1
Patient, CD4+
Healthy control, CD4+
0
20
40
60
80
100
% of CD4+ cells

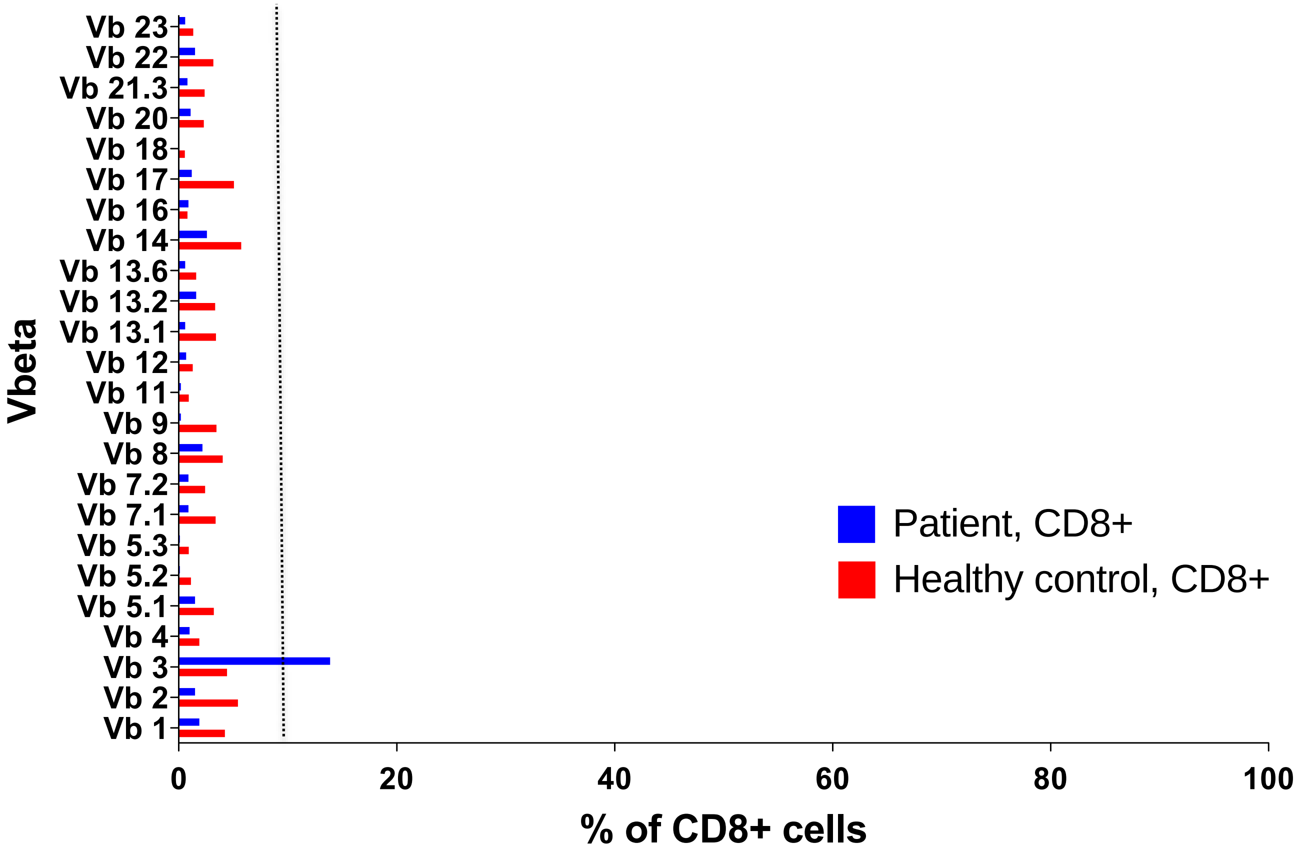

Vbeta
Vb 23
Vb 22
Vb 21.3
Vb 20
Vb 18
Vb 17
Vb 16
Vb 14
Vb 13.6
Vb 13.2
Vb 13.1
Vb 12
Vb 11
Vb 9
Vb 8
Vb 7.2
Vb 7.1
Vb 5.3
Vb 5.2
Vb 5.1
Vb 4
Vb 3
Vb 2
Vb 1
Patient, CD8+
Healthy control, CD8+
0
20
40
60
80
100
% of CD8+ cells

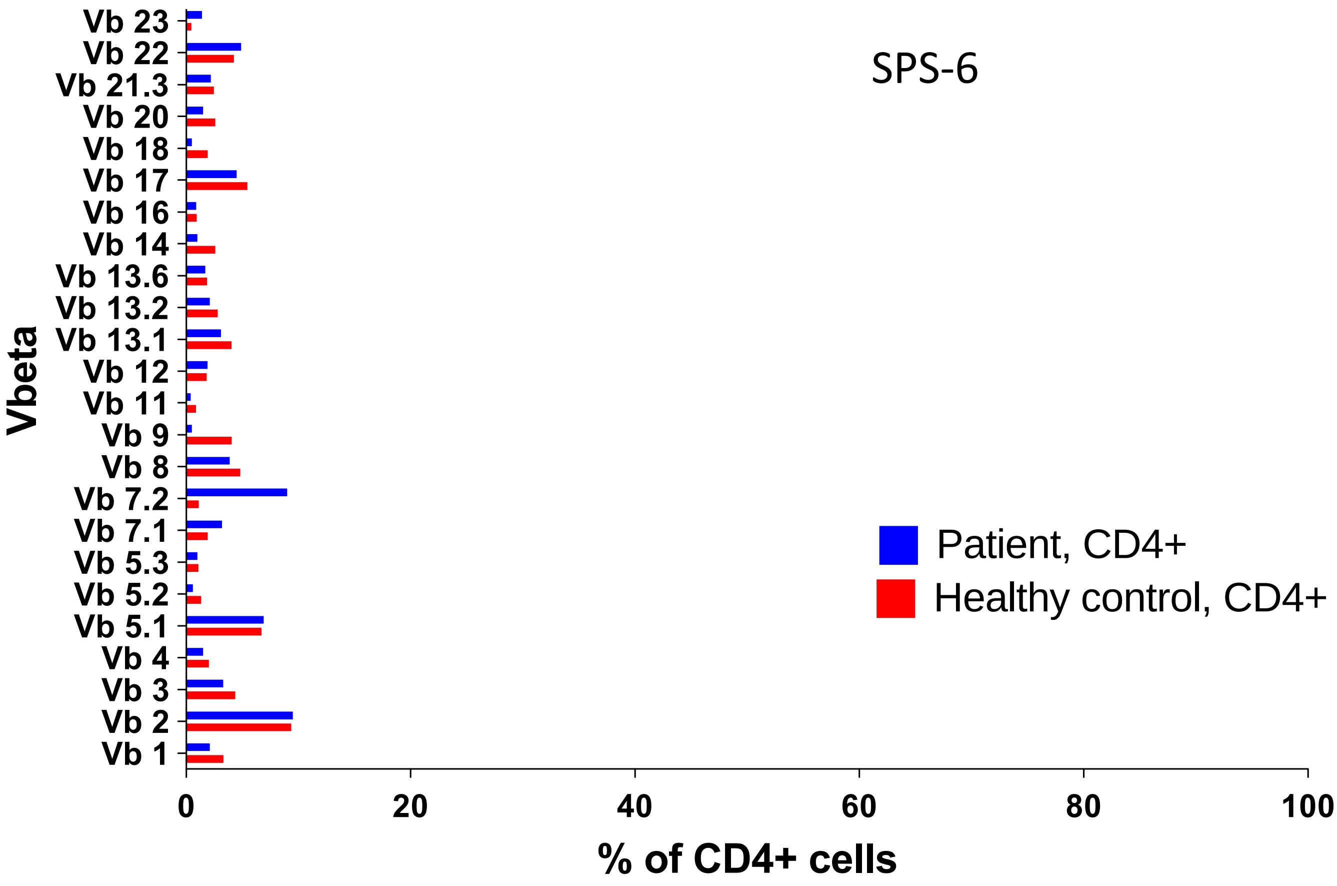
SPS-6
Vbeta
Vb 23
Vb 22
Vb 21.3
Vb 20
Vb 18
Vb 17
Vb 16
Vb 14
Vb 13.6
Vb 13.2
Vb 13.1
Vb 12
Vb 11
Vb 9
Vb 8
Vb 7.2
Vb 7.1
Vb 5.3
Vb 5.2
Vb 5.1
Vb 4
Vb 3
Vb 2
Vb 1
0
20
40
60
80
100
% of CD4+ cells
Patient, CD4+
Healthy control, CD4+

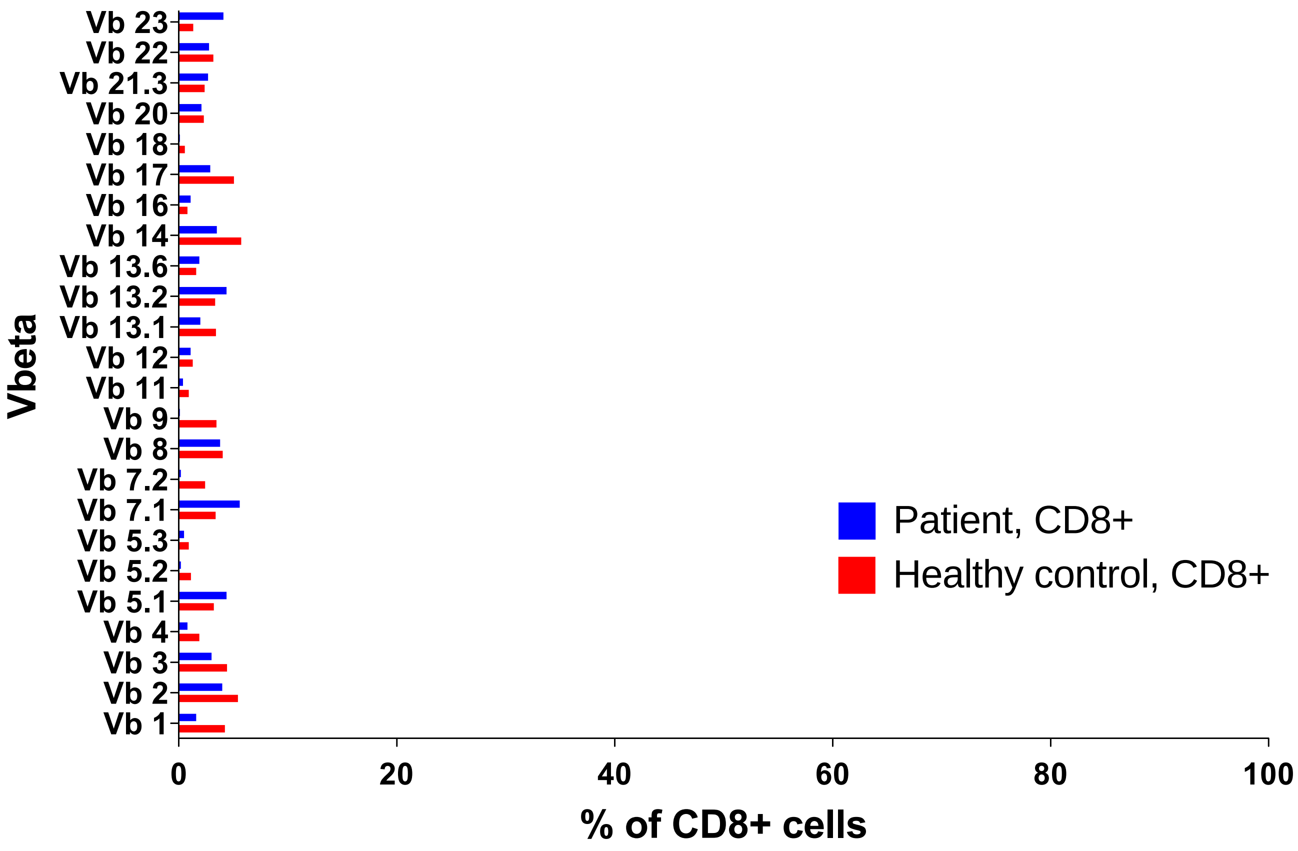
Vbeta
Vb 23
Vb 22
Vb 21.3
Vb 20
Vb 18
Vb 17
Vb 16
Vb 14
Vb 13.6
Vb 13.2
Vb 13.1
Vb 12
Vb 11
Vb 9
Vb 8
Vb 7.2
Vb 7.1
Vb 5.3
Vb 5.2
Vb 5.1
Vb 4
Vb 3
Vb 2
Vb 1
0
20
40
60
80
100
% of CD8+ cells
Patient, CD8+
Healthy control, CD8+

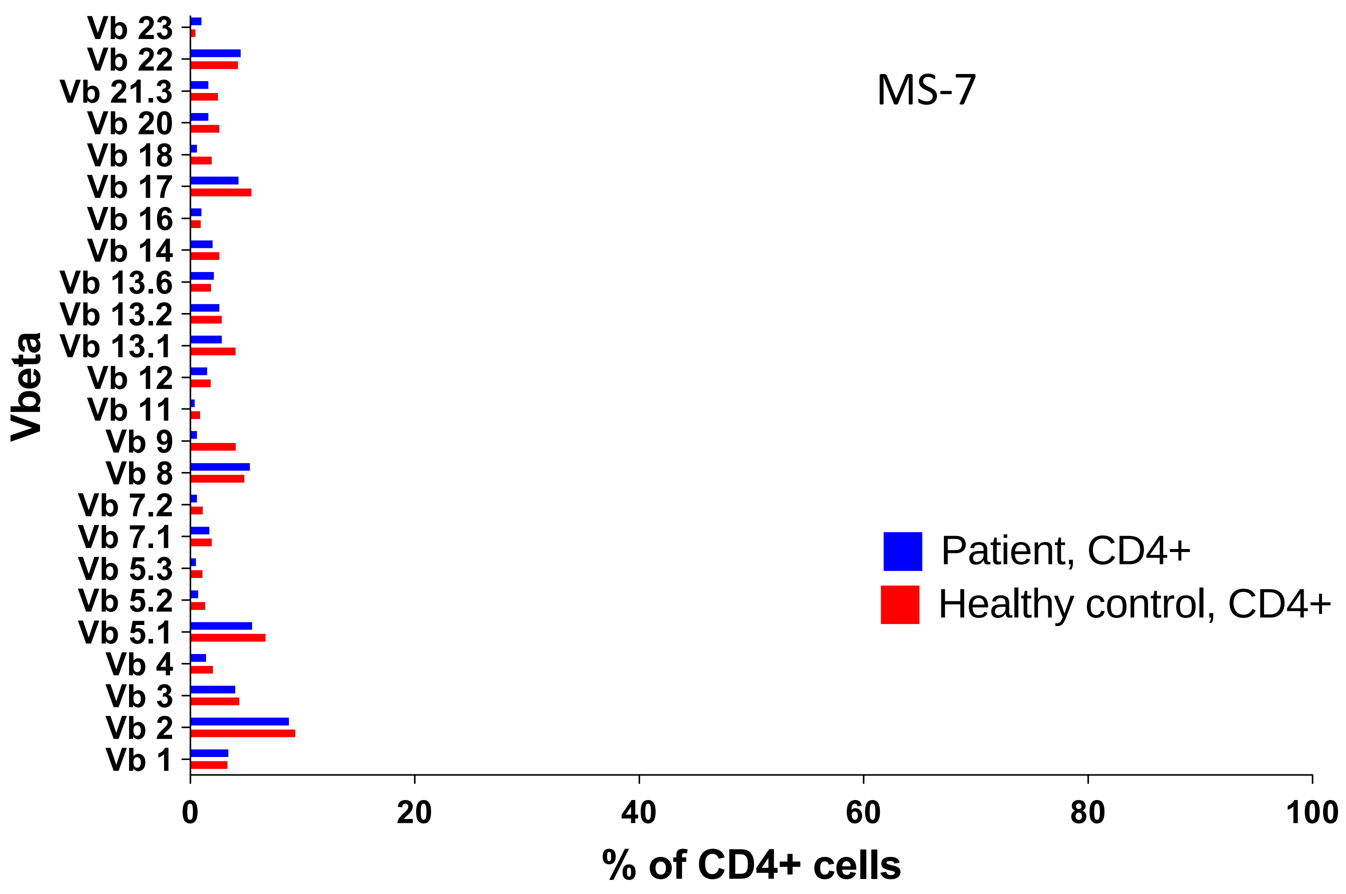

MS-7
Vbeta
Vb 23
Vb 22
Vb 21.3
Vb 20
Vb 18
Vb 17
Vb 16
Vb 14
Vb 13.6
Vb 13.2
Vb 13.1
Vb 12
Vb 11
Vb 9
Vb 8
Vb 7.2
Vb 7.1
Vb 5.3
Vb 5.2
Vb 5.1
Vb 4
Vb 3
Vb 2
Vb 1
0
20
40
60
80
100
% of CD4+ cells
Patient, CD4+
Healthy control, CD4+

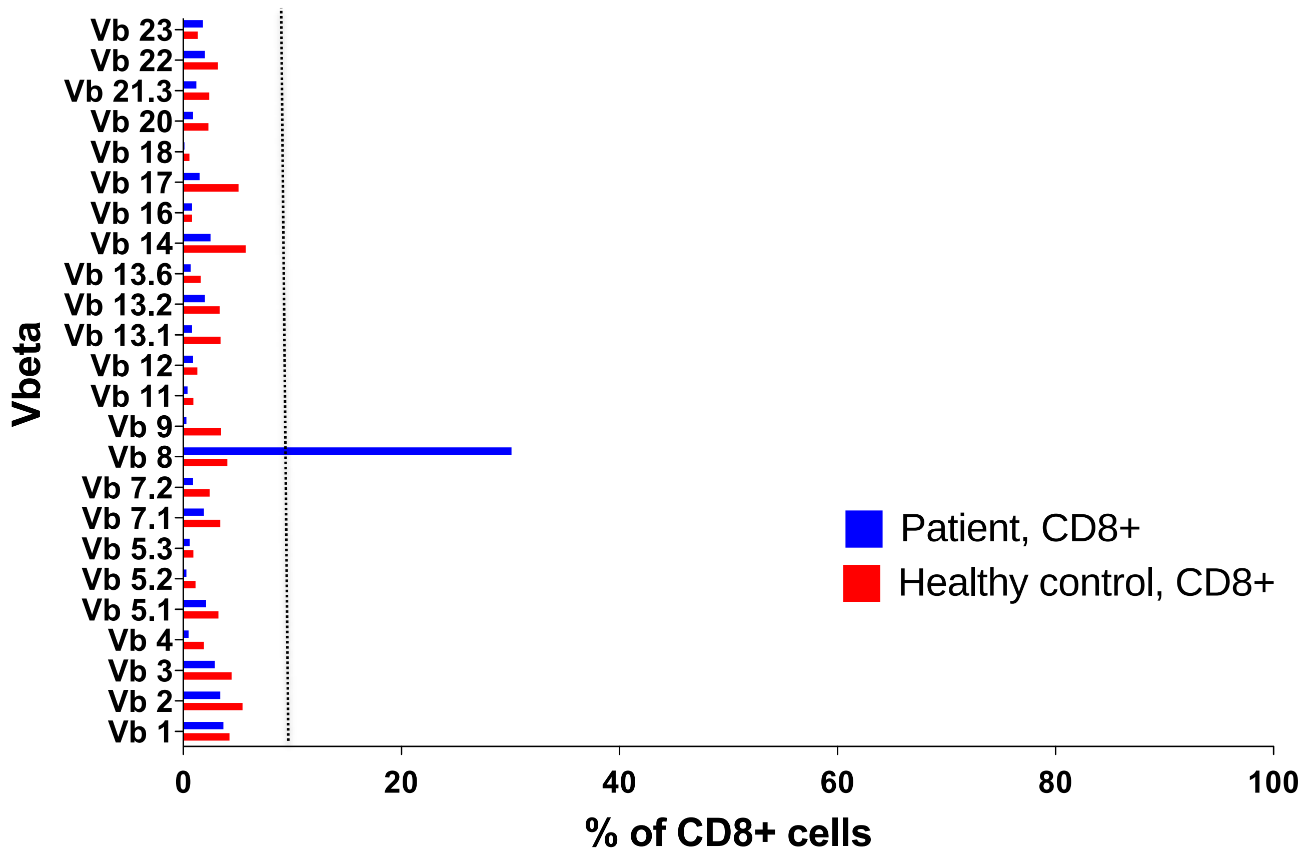

Vbeta
Vb 23
Vb 22
Vb 21.3
Vb 20
Vb 18
Vb 17
Vb 16
Vb 14
Vb 13.6
Vb 13.2
Vb 13.1
Vb 12
Vb 11
Vb 9
Vb 8
Vb 7.2
Vb 7.1
Vb 5.3
Vb 5.2
Vb 5.1
Vb 4
Vb 3
Vb 2
Vb 1
0
20
40
60
80
100
% of CD8+ cells
Patient, CD8+
Healthy control, CD8+

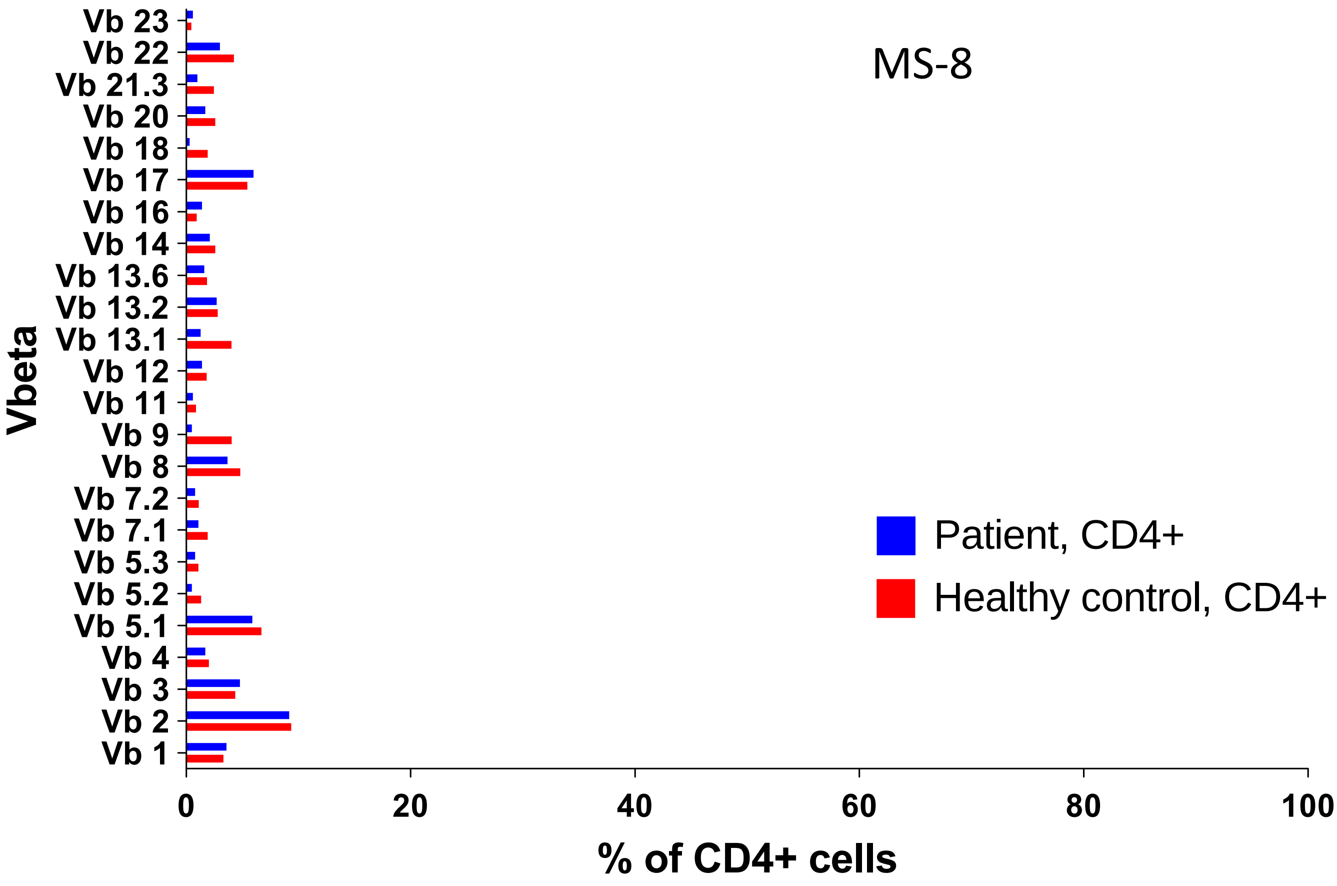
MS-8
Vbeta
Vb 23
Vb 22
Vb 21.3
Vb 20
Vb 18
Vb 17
Vb 16
Vb 14
Vb 13.6
Vb 13.2
Vb 13.1
Vb 12
Vb 11
Vb 9
Vb 8
Vb 7.2
Vb 7.1
Vb 5.3
Vb 5.2
Vb 5.1
Vb 4
Vb 3
Vb 2
Vb 1
0
20
40
60
80
100
% of CD4+ cells
Patient, CD4+
Healthy control, CD4+

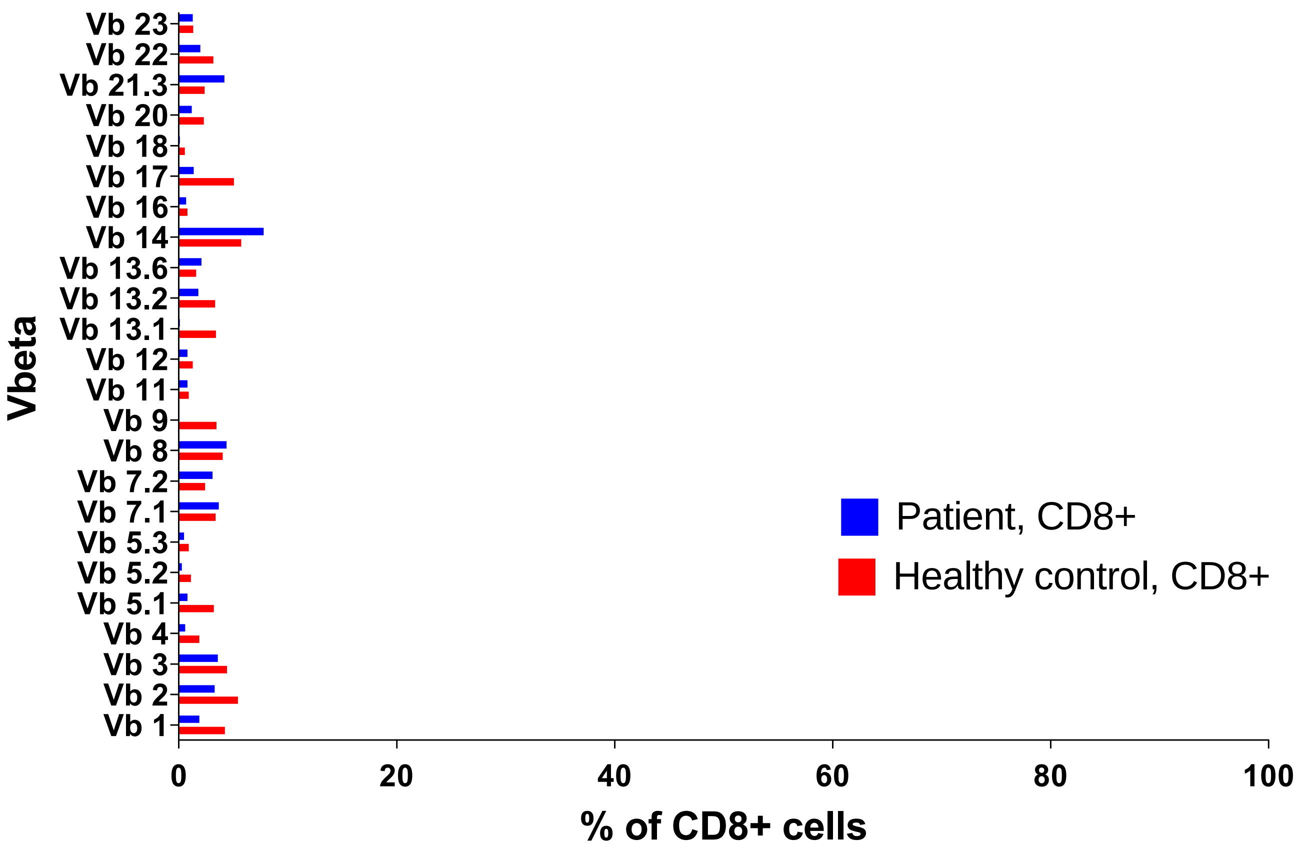
Vbeta
Vb 23
Vb 22
Vb 21.3
Vb 20
Vb 18
Vb 17
Vb 16
Vb 14
Vb 13.6
Vb 13.2
Vb 13.1
Vb 12
Vb 11
Vb 9
Vb 8
Vb 7.2
Vb 7.1
Vb 5.3
Vb 5.2
Vb 5.1
Vb 4
Vb 3
Vb 2
Vb 1
0
20
40
60
80
100
% of CD8+ cells
Patient, CD8+
Healthy control, CD8+

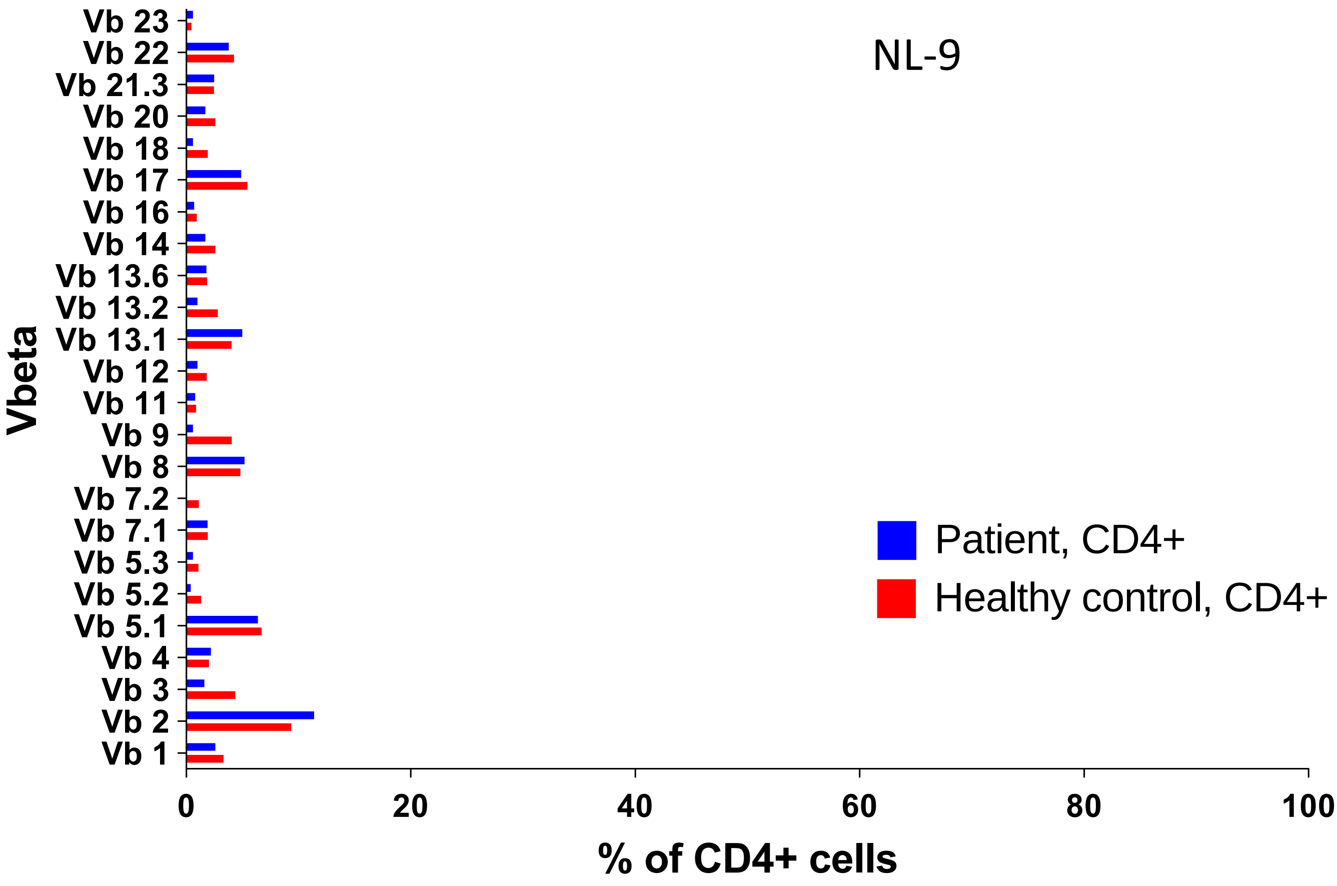
NL-9
Vbeta
Vb 23
Vb 22
Vb 21.3
Vb 20
Vb 18
Vb 17
Vb 16
Vb 14
Vb 13.6
Vb 13.2
Vb 13.1
Vb 12
Vb 11
Vb 9
Vb 8
Vb 7.2
Vb 7.1
Vb 5.3
Vb 5.2
Vb 5.1
Vb 4
Vb 3
Vb 2
Vb 1
0
20
40
60
80
100
% of CD4+ cells
Patient, CD4+
Healthy control, CD4+

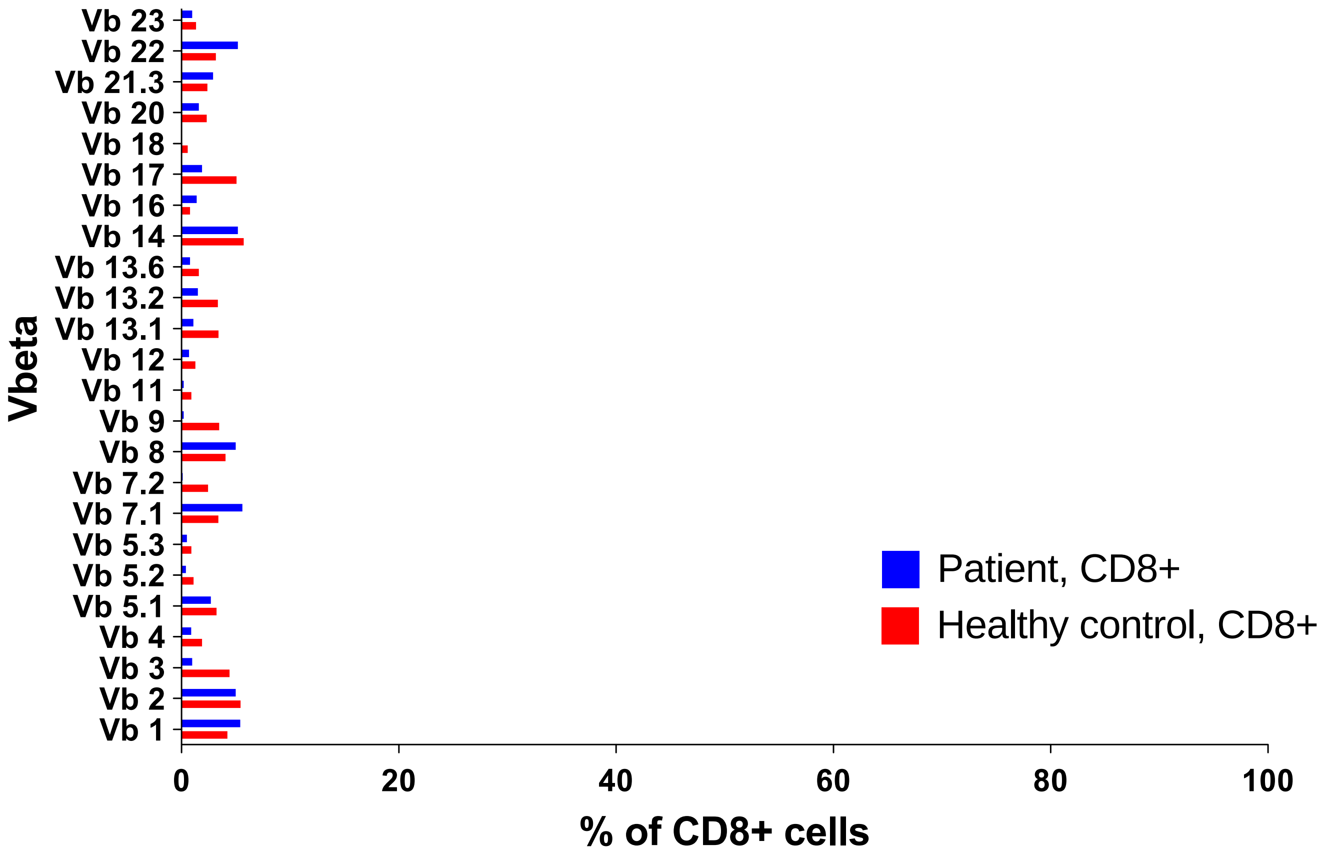
Vbeta
Vb 23
Vb 22
Vb 21.3
Vb 20
Vb 18
Vb 17
Vb 16
Vb 14
Vb 13.6
Vb 13.2
Vb 13.1
Vb 12
Vb 11
Vb 9
Vb 8
Vb 7.2
Vb 7.1
Vb 5.3
Vb 5.2
Vb 5.1
Vb 4
Vb 3
Vb 2
Vb 1
0
20
40
60
80
100
% of CD8+ cells
Patient, CD8+
Healthy control, CD8+

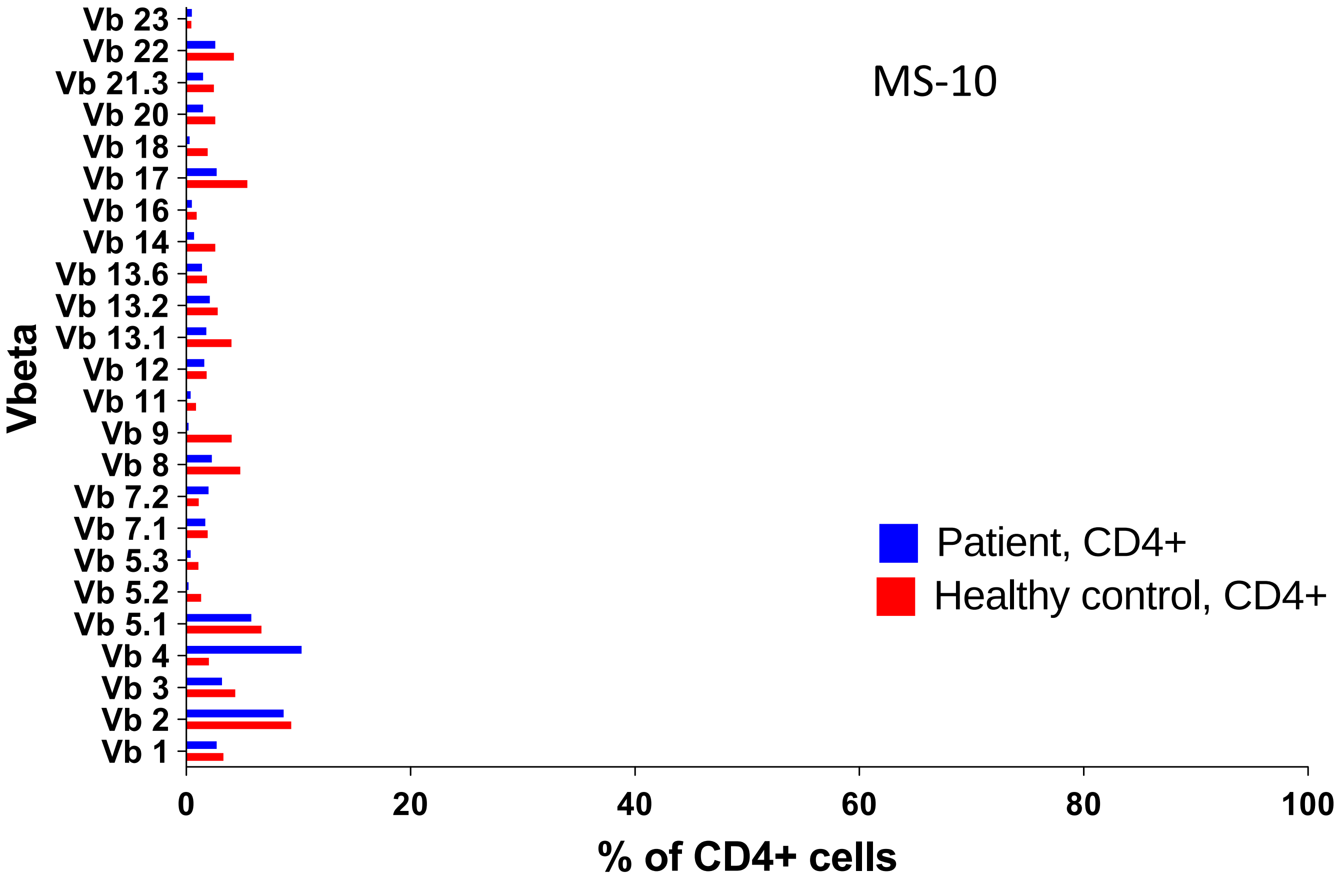
MS-10
Vbeta
Vb 23
Vb 22
Vb 21.3
Vb 20
Vb 18
Vb 17
Vb 16
Vb 14
Vb 13.6
Vb 13.2
Vb 13.1
Vb 12
Vb 11
Vb 9
Vb 8
Vb 7.2
Vb 7.1
Vb 5.3
Vb 5.2
Vb 5.1
Vb 4
Vb 3
Vb 2
Vb 1
0
20
40
60
80
100
% of CD4+ cells
Patient, CD4+
Healthy control, CD4+

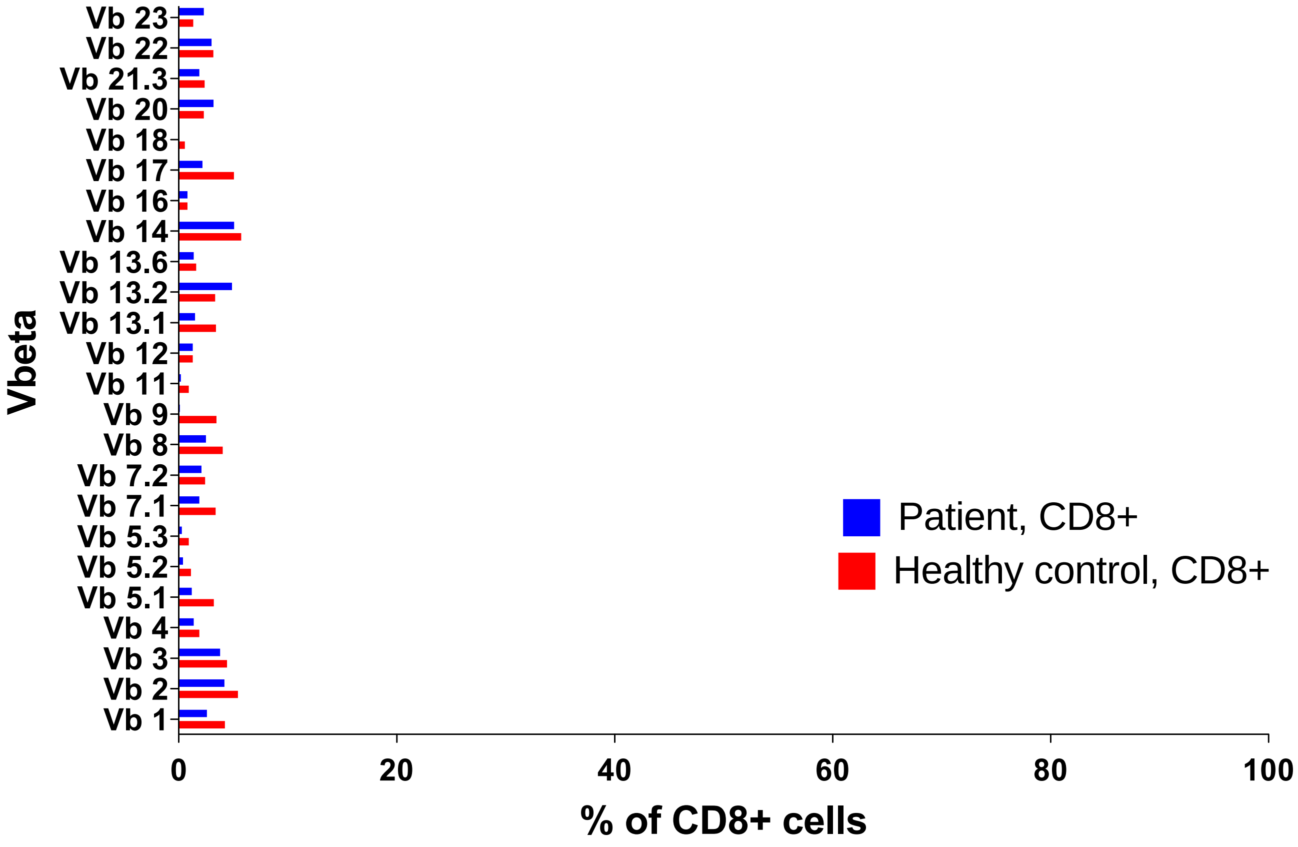
Vbeta
Vb 23
Vb 22
Vb 21.3
Vb 20
Vb 18
Vb 17
Vb 16
Vb 14
Vb 13.6
Vb 13.2
Vb 13.1
Vb 12
Vb 11
Vb 9
Vb 8
Vb 7.2
Vb 7.1
Vb 5.3
Vb 5.2
Vb 5.1
Vb 4
Vb 3
Vb 2
Vb 1
0
20
40
60
80
100
% of CD8+ cells
Patient, CD8+
Healthy control, CD8+

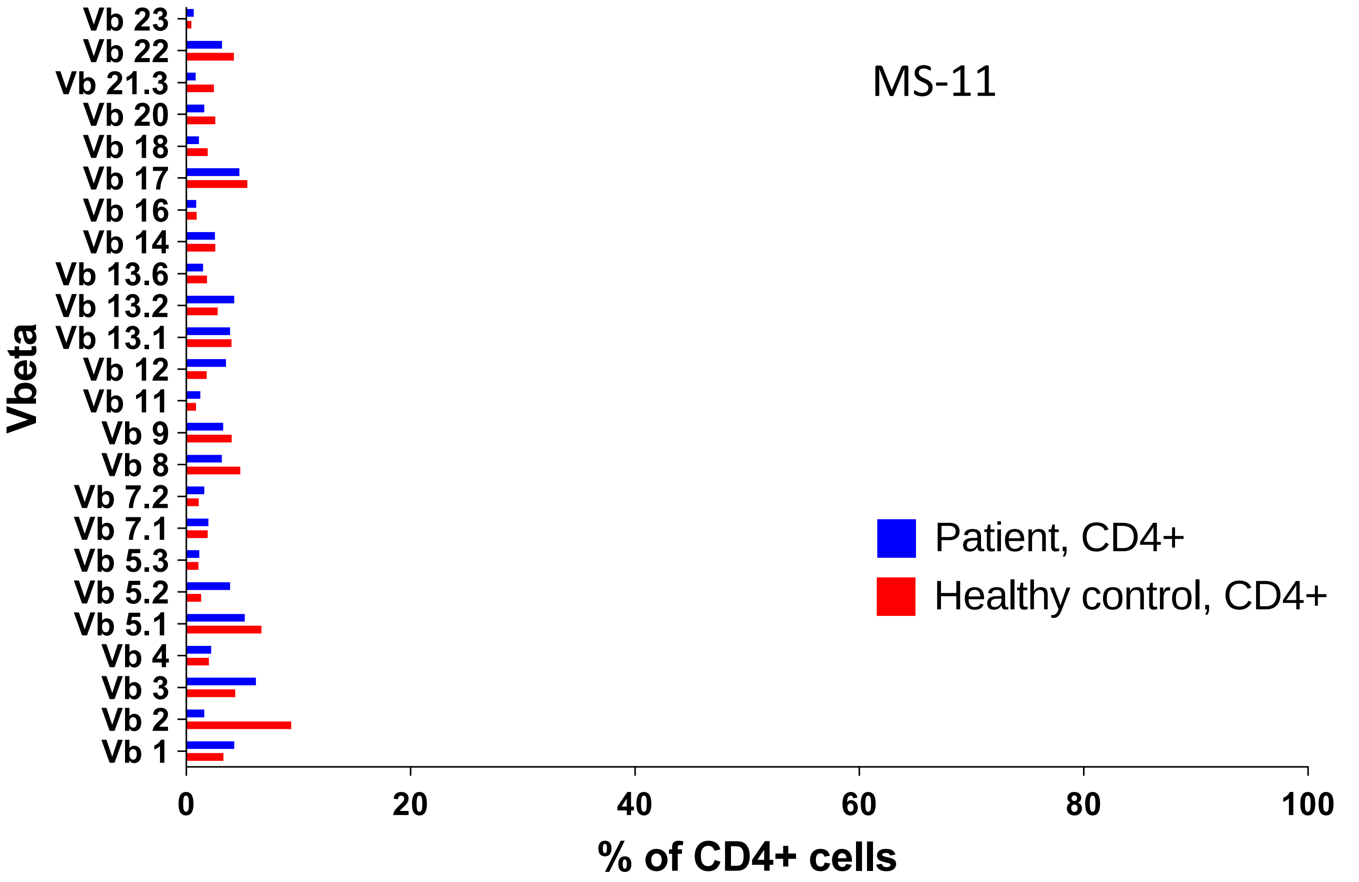
MS-11
Vbeta
Vb 23
Vb 22
Vb 21.3
Vb 20
Vb 18
Vb 17
Vb 16
Vb 14
Vb 13.6
Vb 13.2
Vb 13.1
Vb 12
Vb 11
Vb 9
Vb 8
Vb 7.2
Vb 7.1
Vb 5.3
Vb 5.2
Vb 5.1
Vb 4
Vb 3
Vb 2
Vb 1
0
20
40
60
80
100
% of CD4+ cells
Patient, CD4+
Healthy control, CD4+

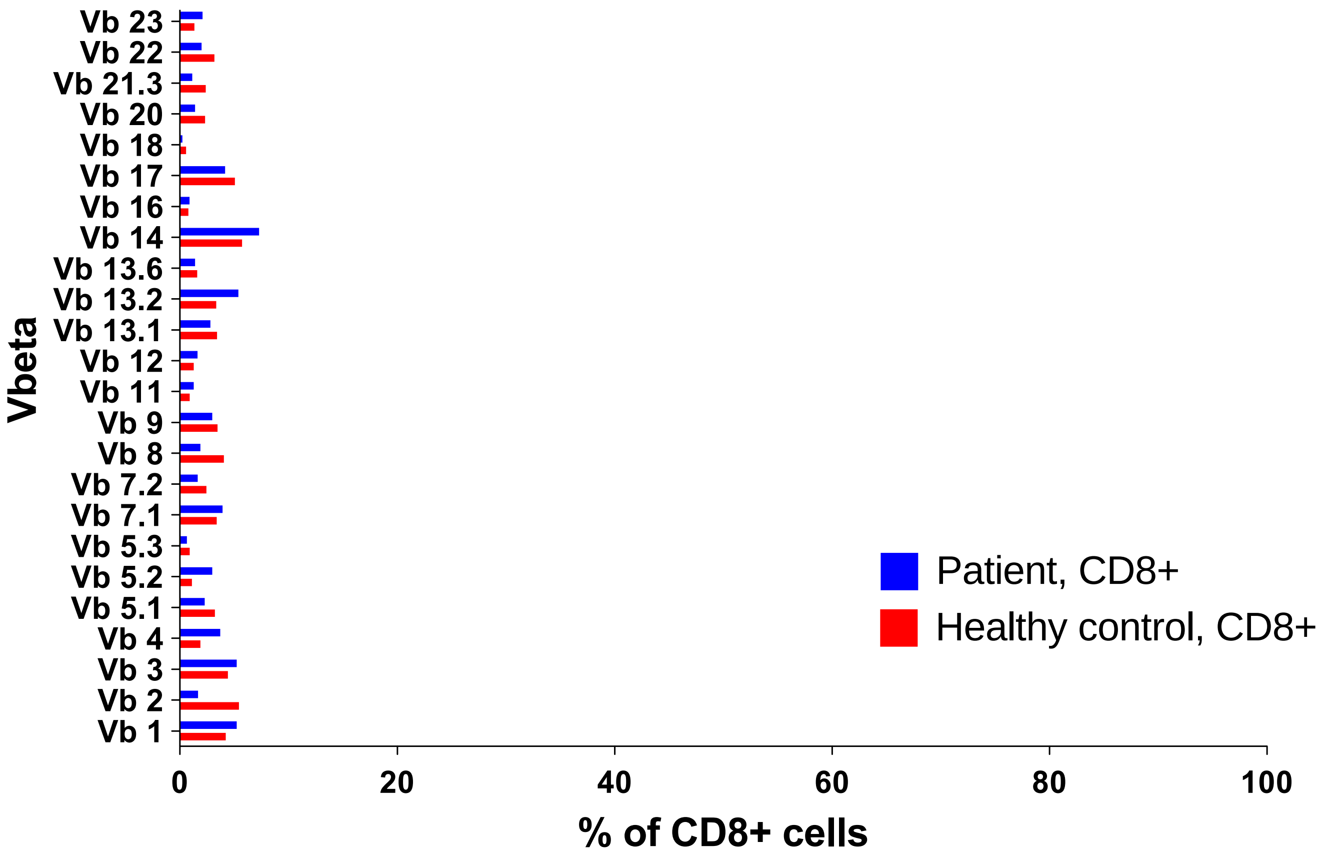
Vbeta
Vb 23
Vb 22
Vb 21.3
Vb 20
Vb 18
Vb 17
Vb 16
Vb 14
Vb 13.6
Vb 13.2
Vb 13.1
Vb 12
Vb 11
Vb 9
Vb 8
Vb 7.2
Vb 7.1
Vb 5.3
Vb 5.2
Vb 5.1
Vb 4
Vb 3
Vb 2
Vb 1
0
20
40
60
80
100
% of CD8+ cells
Patient, CD8+
Healthy control, CD8+

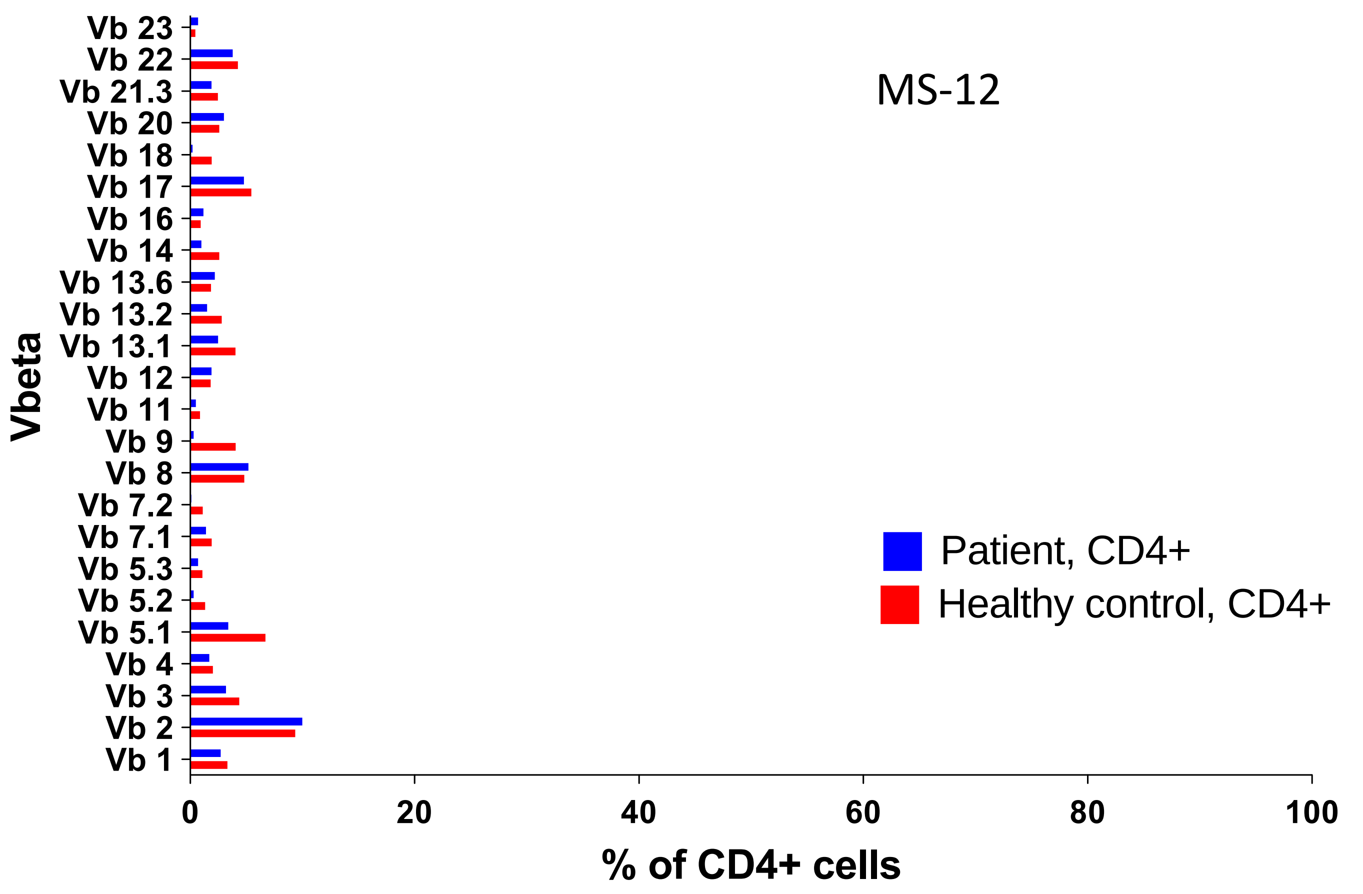
MS-12
Vbeta
Vb 23
Vb 22
Vb 21.3
Vb 20
Vb 18
Vb 17
Vb 16
Vb 14
Vb 13.6
Vb 13.2
Vb 13.1
Vb 12
Vb 11
Vb 9
Vb 8
Vb 7.2
Vb 7.1
Vb 5.3
Vb 5.2
Vb 5.1
Vb 4
Vb 3
Vb 2
Vb 1
0
20
40
60
80
100
% of CD4+ cells
Patient, CD4+
Healthy control, CD4+

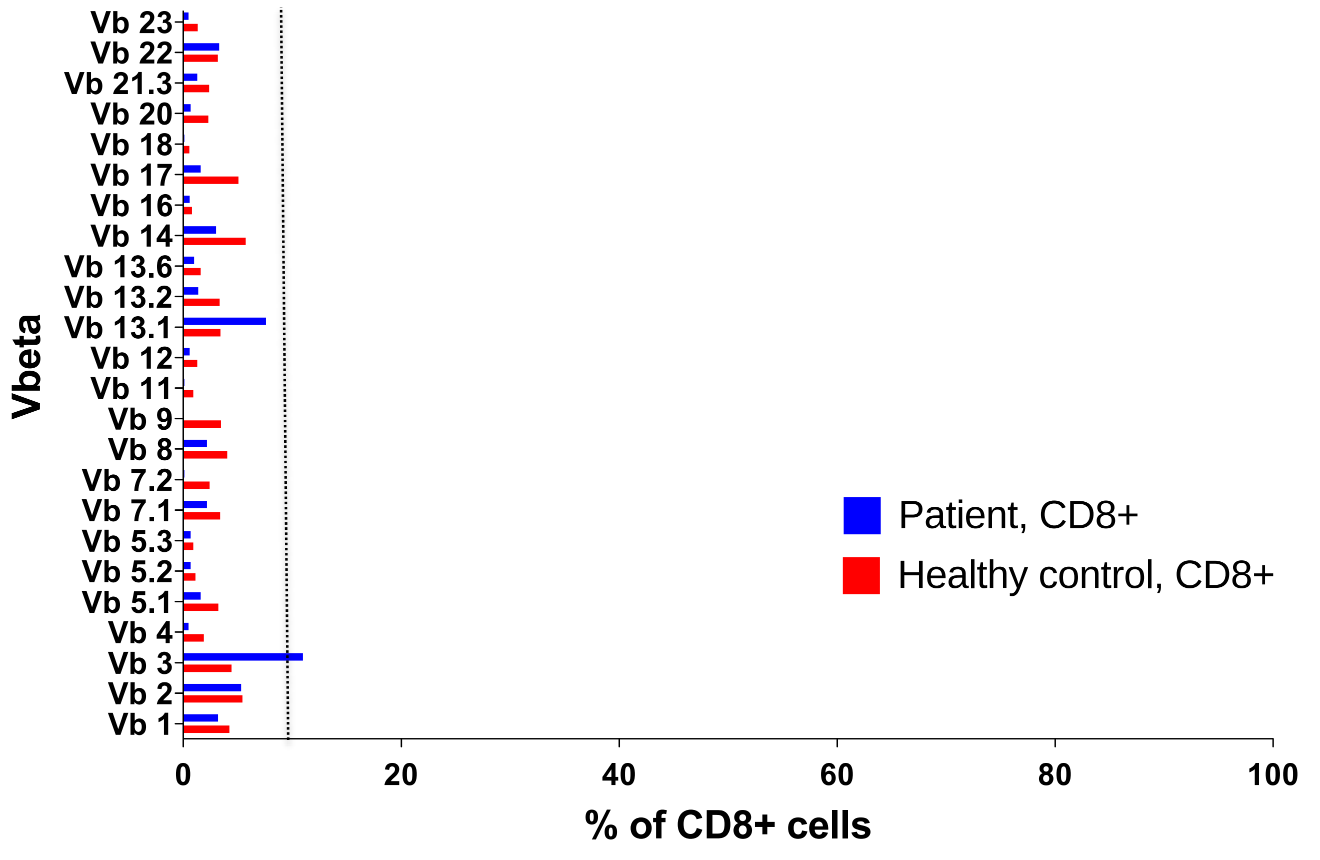
Vbeta
Vb 23
Vb 22
Vb 21.3
Vb 20
Vb 18
Vb 17
Vb 16
Vb 14
Vb 13.6
Vb 13.2
Vb 13.1
Vb 12
Vb 11
Vb 9
Vb 8
Vb 7.2
Vb 7.1
Vb 5.3
Vb 5.2
Vb 5.1
Vb 4
Vb 3
Vb 2
Vb 1
0
20
40
60
80
100
% of CD8+ cells
Patient, CD8+
Healthy control, CD8+

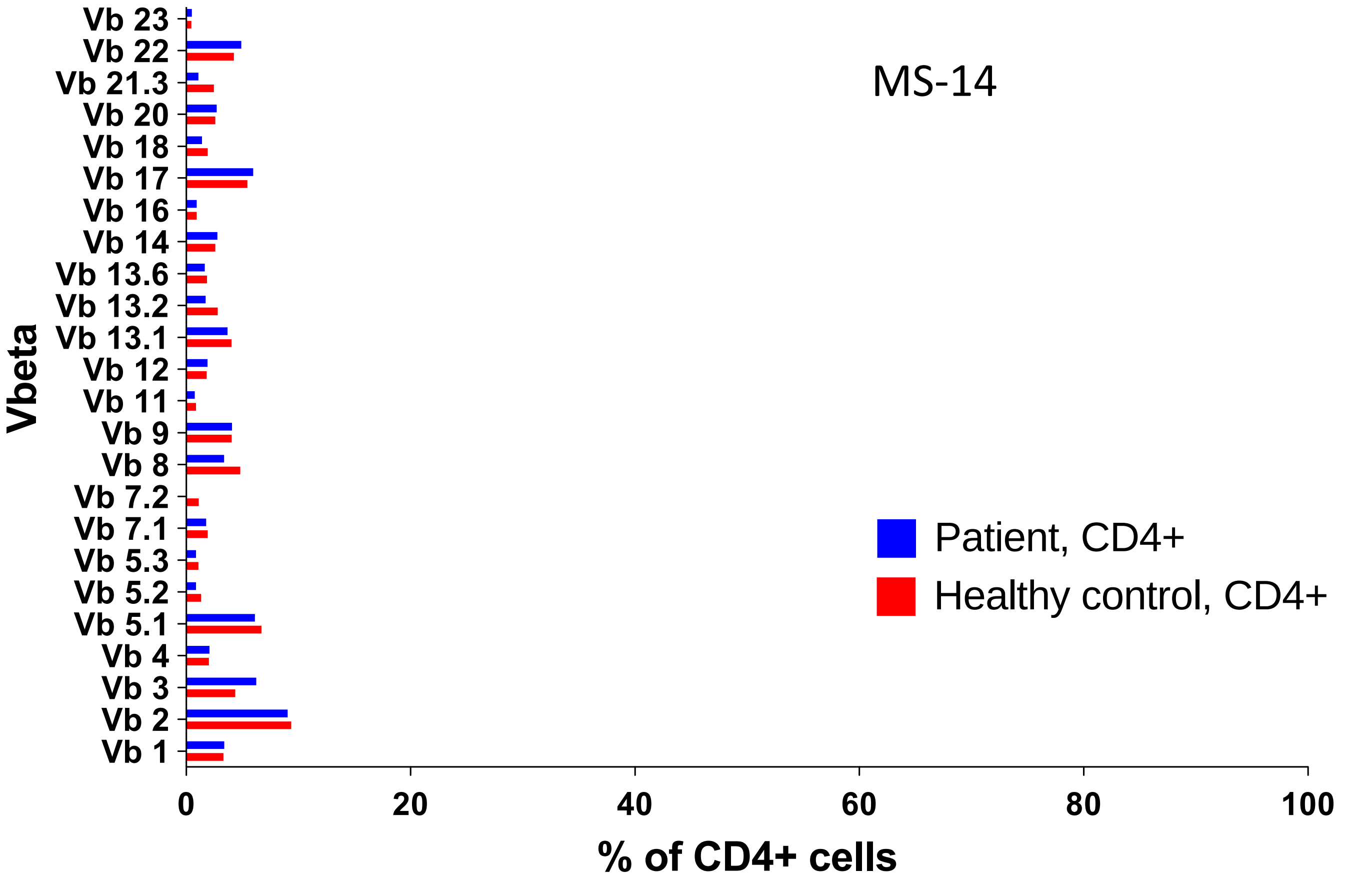
MS-14
Vbeta
Vb 23
Vb 22
Vb 21.3
Vb 20
Vb 18
Vb 17
Vb 16
Vb 14
Vb 13.6
Vb 13.2
Vb 13.1
Vb 12
Vb 11
Vb 9
Vb 8
Vb 7.2
Vb 7.1
Vb 5.3
Vb 5.2
Vb 5.1
Vb 4
Vb 3
Vb 2
Vb 1
0
20
40
60
80
100
% of CD4+ cells
Patient, CD4+
Healthy control, CD4+

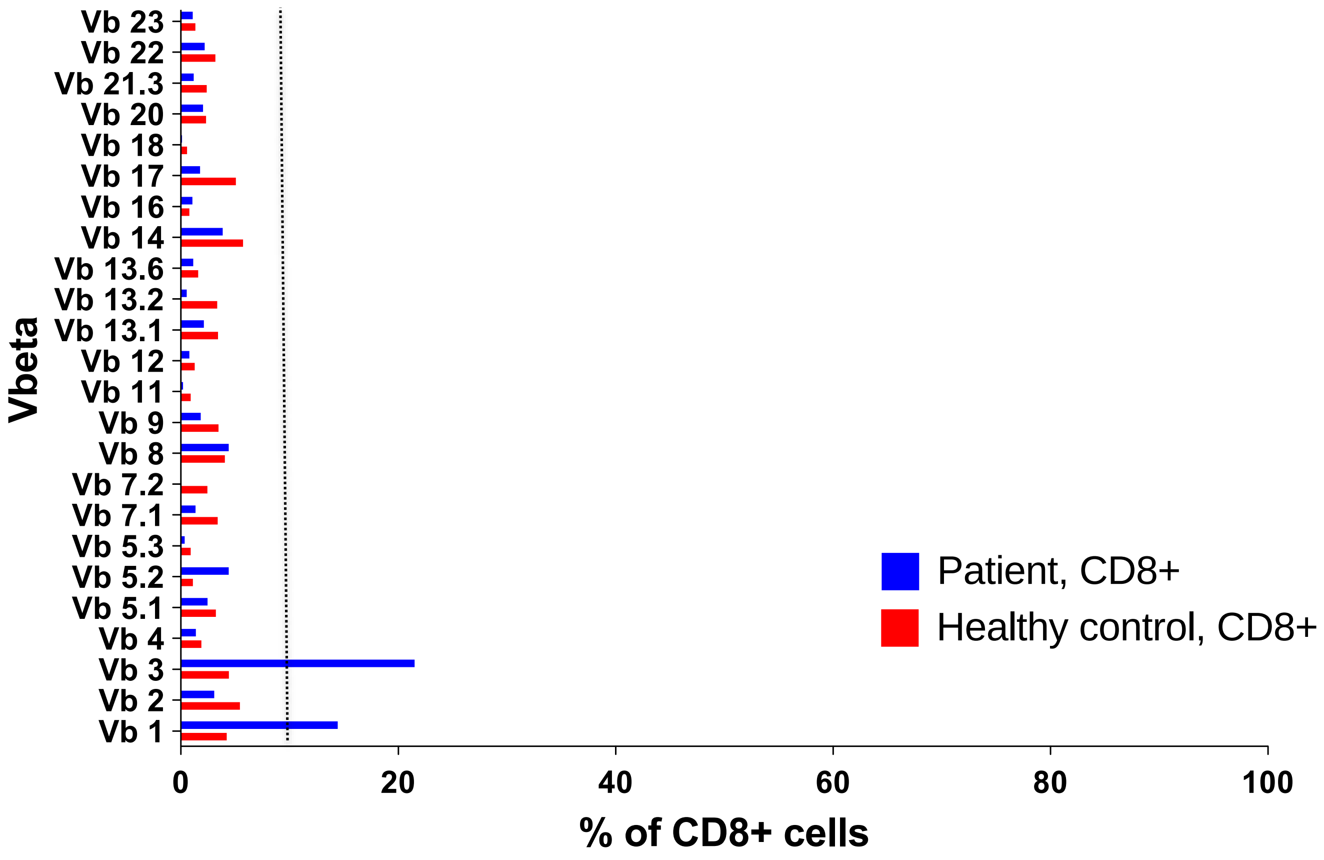
Vbeta
Vb 23
Vb 22
Vb 21.3
Vb 20
Vb 18
Vb 17
Vb 16
Vb 14
Vb 13.6
Vb 13.2
Vb 13.1
Vb 12
Vb 11
Vb 9
Vb 8
Vb 7.2
Vb 7.1
Vb 5.3
Vb 5.2
Vb 5.1
Vb 4
Vb 3
Vb 2
Vb 1
0
20
40
60
80
100
% of CD8+ cells
Patient, CD8+
Healthy control, CD8+

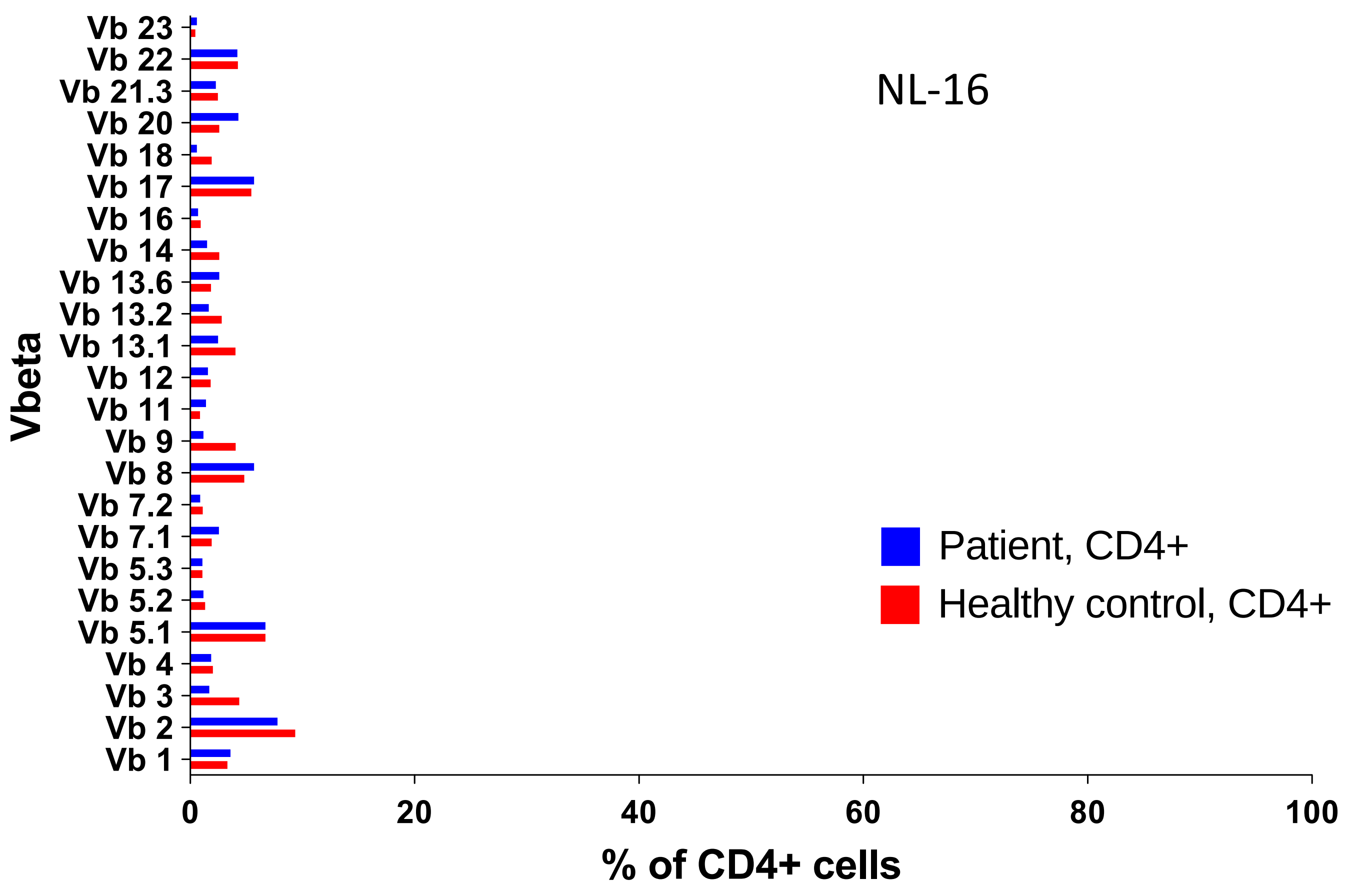
NL-16
Vbeta
Vb 23
Vb 22
Vb 21.3
Vb 20
Vb 18
Vb 17
Vb 16
Vb 14
Vb 13.6
Vb 13.2
Vb 13.1
Vb 12
Vb 11
Vb 9
Vb 8
Vb 7.2
Vb 7.1
Vb 5.3
Vb 5.2
Vb 5.1
Vb 4
Vb 3
Vb 2
Vb 1
0
20
40
60
80
100
% of CD4+ cells
Patient, CD4+
Healthy control, CD4+

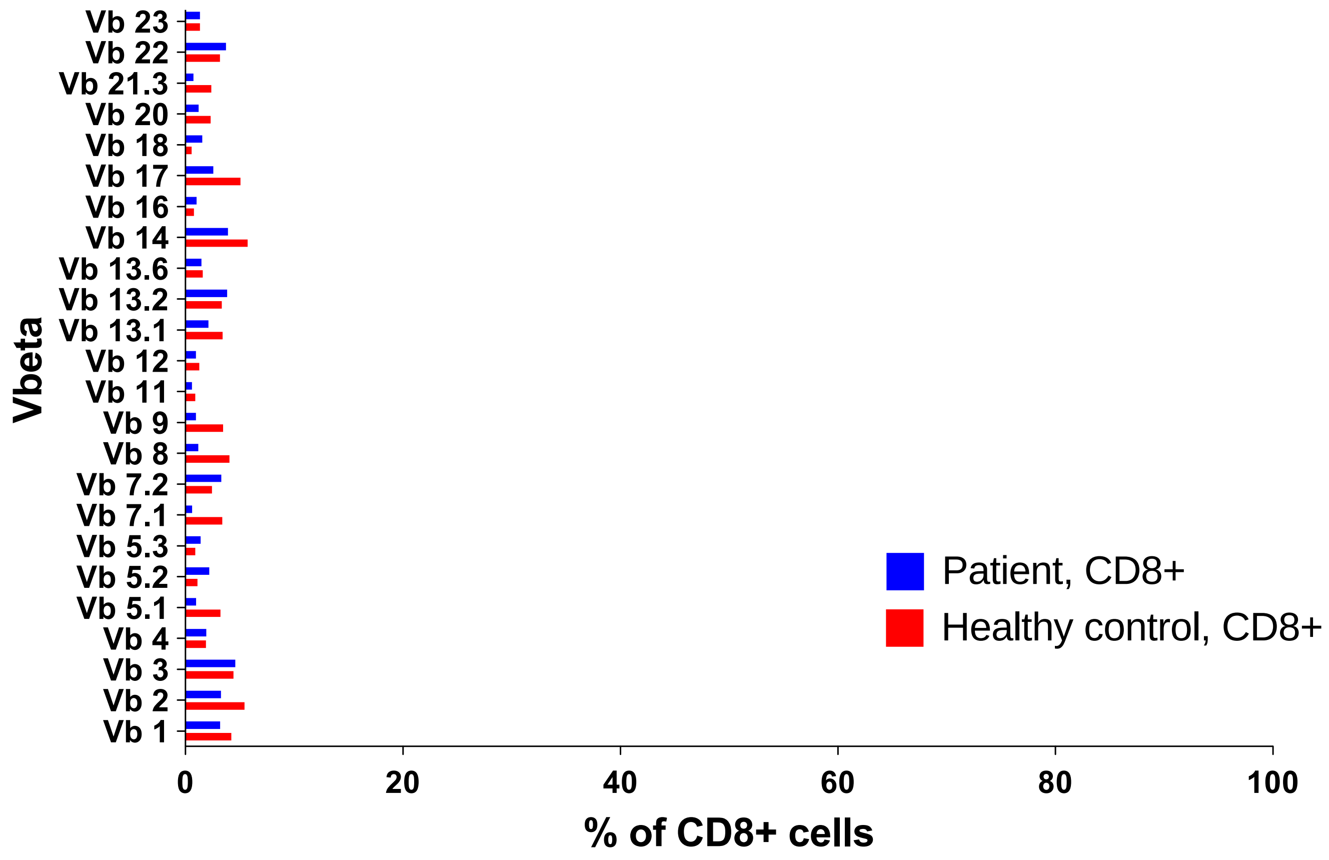
Vbeta
Vb 23
Vb 22
Vb 21.3
Vb 20
Vb 18
Vb 17
Vb 16
Vb 14
Vb 13.6
Vb 13.2
Vb 13.1
Vb 12
Vb 11
Vb 9
Vb 8
Vb 7.2
Vb 7.1
Vb 5.3
Vb 5.2
Vb 5.1
Vb 4
Vb 3
Vb 2
Vb 1
0
20
40
60
80
100
% of CD8+ cells
Patient, CD8+
Healthy control, CD8+

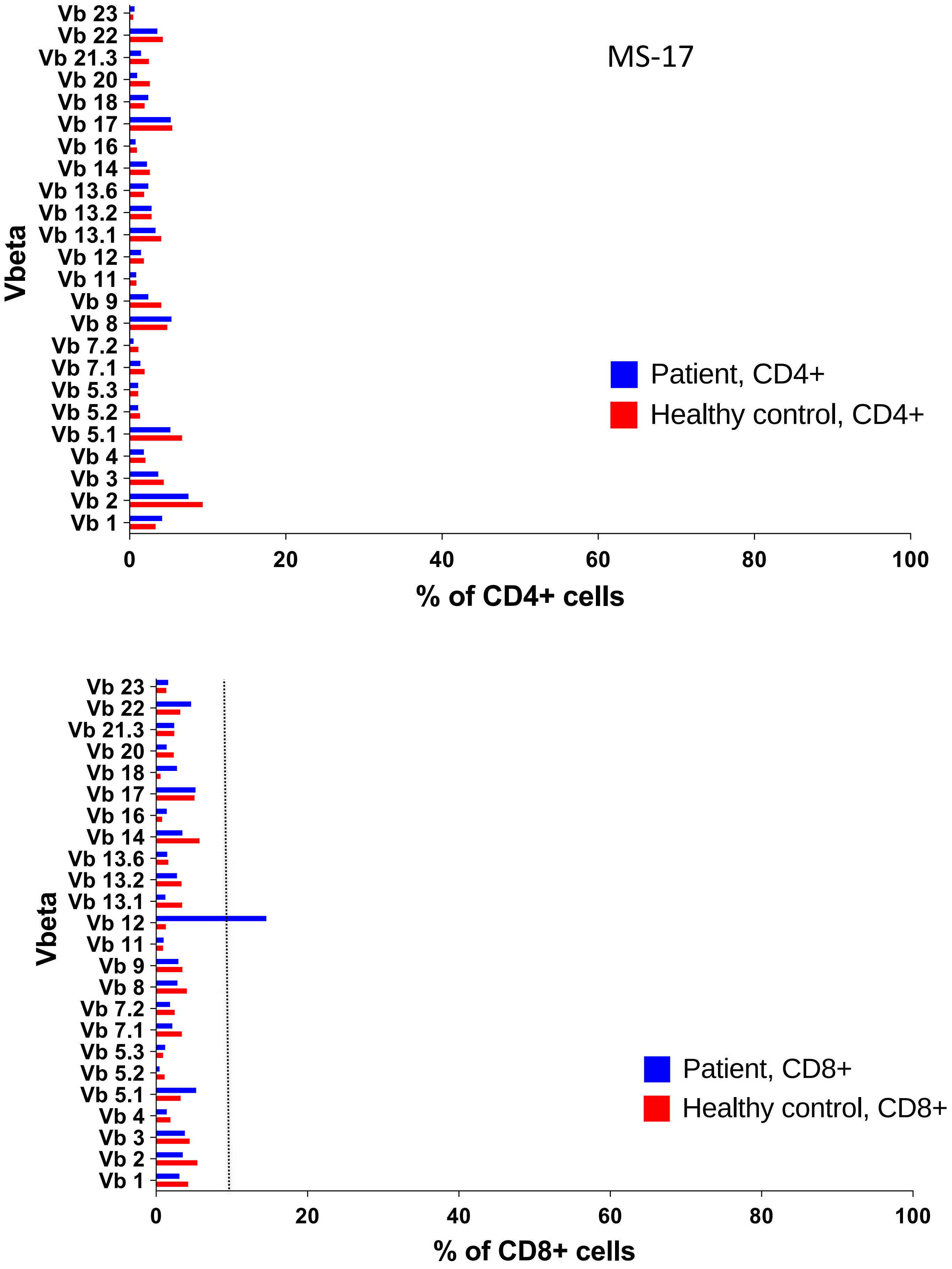
MS-17
Vbeta
Vb 23
Vb 22
Vb 21.3
Vb 20
Vb 18
Vb 17
Vb 16
Vb 14
Vb 13.6
Vb 13.2
Vb 13.1
Vb 12
Vb 11
Vb 9
Vb 8
Vb 7.2
Vb 7.1
Vb 5.3
Vb 5.2
Vb 5.1
Vb 4
Vb 3
Vb 2
Vb 1
0
20
40
60
80
100
% of CD4+ cells
Patient, CD4+
Healthy control, CD4+
% of CD8+ cells
Patient, CD8+
Healthy control, CD8+

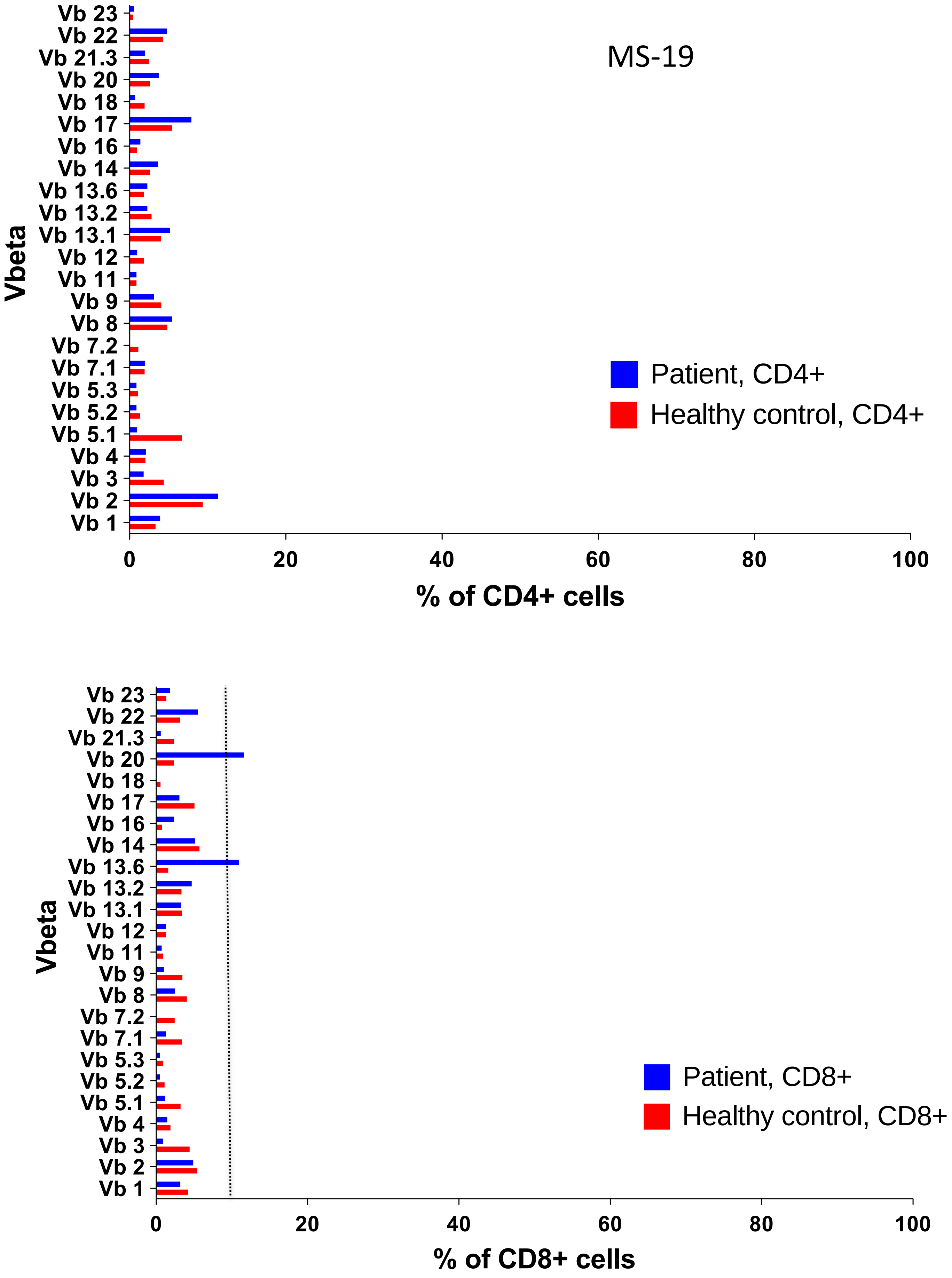

MS-19
Vbeta
Vb 23
Vb 22
Vb 21.3
Vb 20
Vb 18
Vb 17
Vb 16
Vb 14
Vb 13.6
Vb 13.2
Vb 13.1
Vb 12
Vb 11
Vb 9
Vb 8
Vb 7.2
Vb 7.1
Vb 5.3
Vb 5.2
Vb 5.1
Vb 4
Vb 3
Vb 2
Vb 1
0
20
40
60
80
100
% of CD4+ cells
Patient, CD4+
Healthy control, CD4+
Vbeta
Vb 23
Vb 22
Vb 21.3
Vb 20
Vb 18
Vb 17
Vb 16
Vb 14
Vb 13.6
Vb 13.2
Vb 13.1
Vb 12
Vb 11
Vb 9
Vb 8
Vb 7.2
Vb 7.1
Vb 5.3
Vb 5.2
Vb 5.1
Vb 4
Vb 3
Vb 2
Vb 1
0
20
40
60
80
100
% of CD8+ cells
Patient, CD8+
Healthy control, CD8+

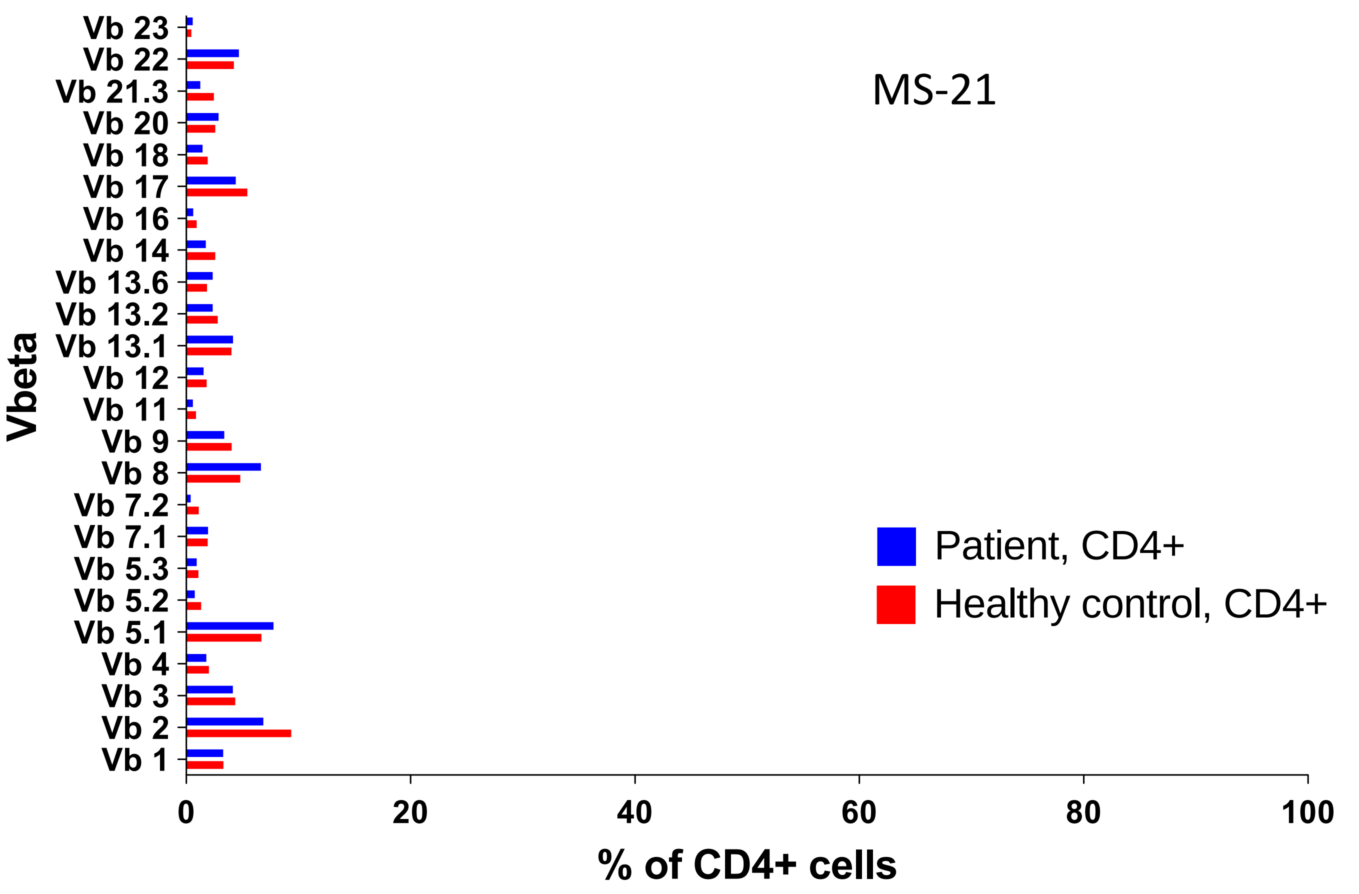
MS-21
Vbeta
Vb 23
Vb 22
Vb 21.3
Vb 20
Vb 18
Vb 17
Vb 16
Vb 14
Vb 13.6
Vb 13.2
Vb 13.1
Vb 12
Vb 11
Vb 9
Vb 8
Vb 7.2
Vb 7.1
Vb 5.3
Vb 5.2
Vb 5.1
Vb 4
Vb 3
Vb 2
Vb 1
0
20
40
60
80
100
% of CD4+ cells
Patient, CD4+
Healthy control, CD4+

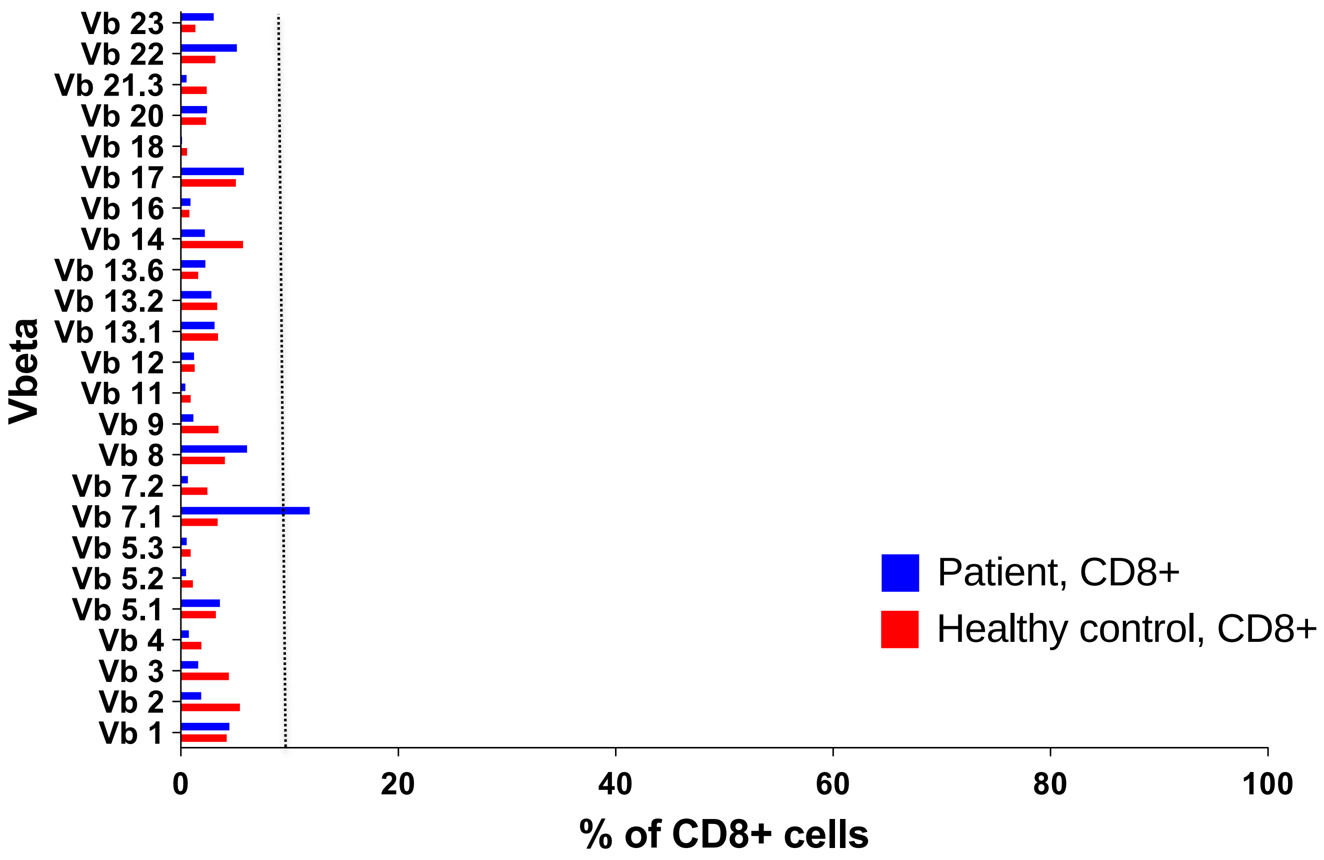
Vbeta
Vb 23
Vb 22
Vb 21.3
Vb 20
Vb 18
Vb 17
Vb 16
Vb 14
Vb 13.6
Vb 13.2
Vb 13.1
Vb 12
Vb 11
Vb 9
Vb 8
Vb 7.2
Vb 7.1
Vb 5.3
Vb 5.2
Vb 5.1
Vb 4
Vb 3
Vb 2
Vb 1
0
20
40
60
80
100
% of CD8+ cells
Patient, CD8+
Healthy control, CD8+

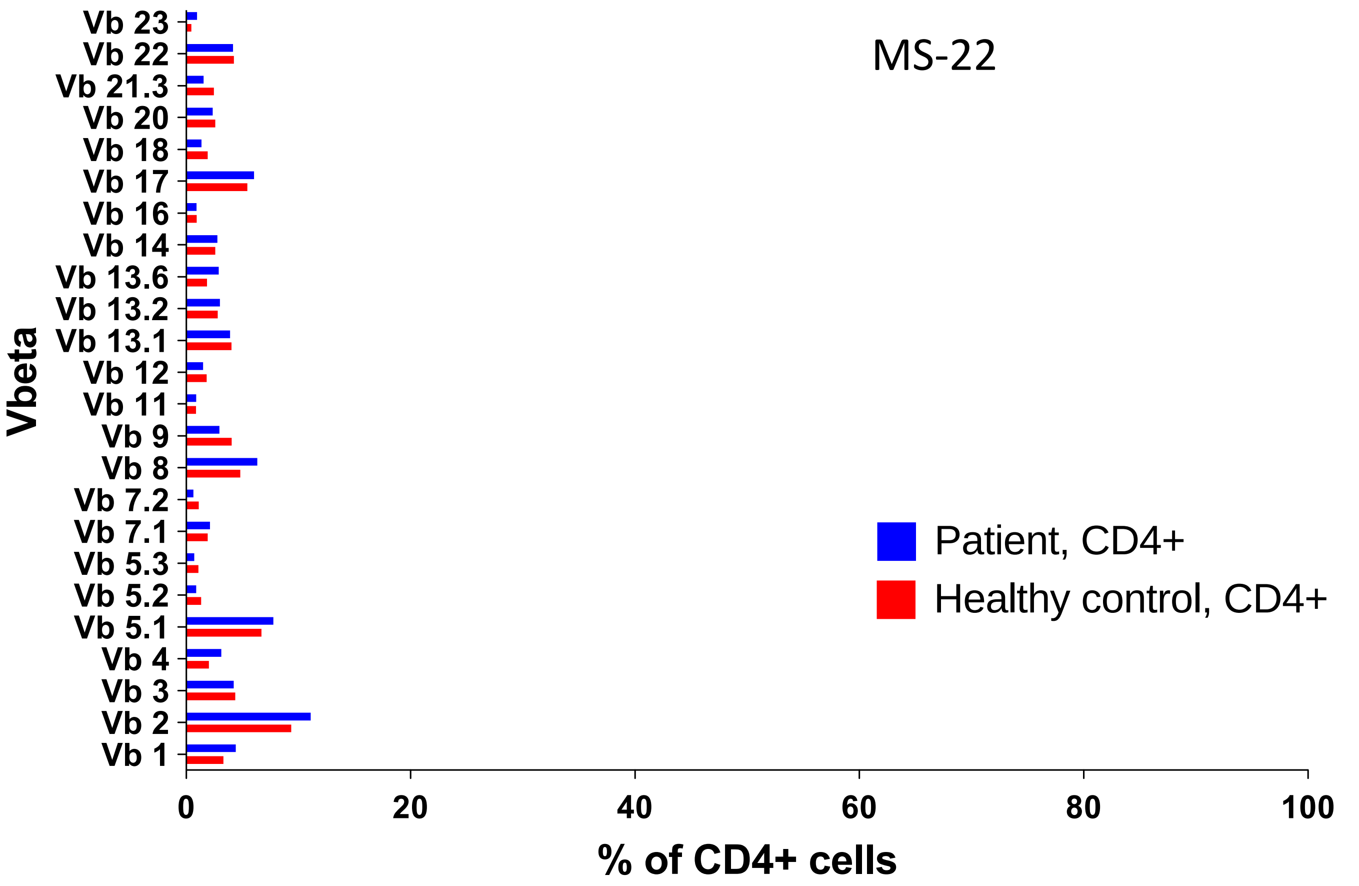
MS-22
Vb 23
Vb 22
Vb 21.3
Vb 20
Vb 18
Vb 17
Vb 16
Vb 14
Vb 13.6
Vb 13.2
Vb 13.1
Vb 12
Vb 11
Vb 9
Vb 8
Vb 7.2
Vb 7.1
Vb 5.3
Vb 5.2
Vb 5.1
Vb 4
Vb 3
Vb 2
Vb 1
Vbeta
Patient, CD4+
Healthy control, CD4+
0
20
40
60
80
100
% of CD4+ cells

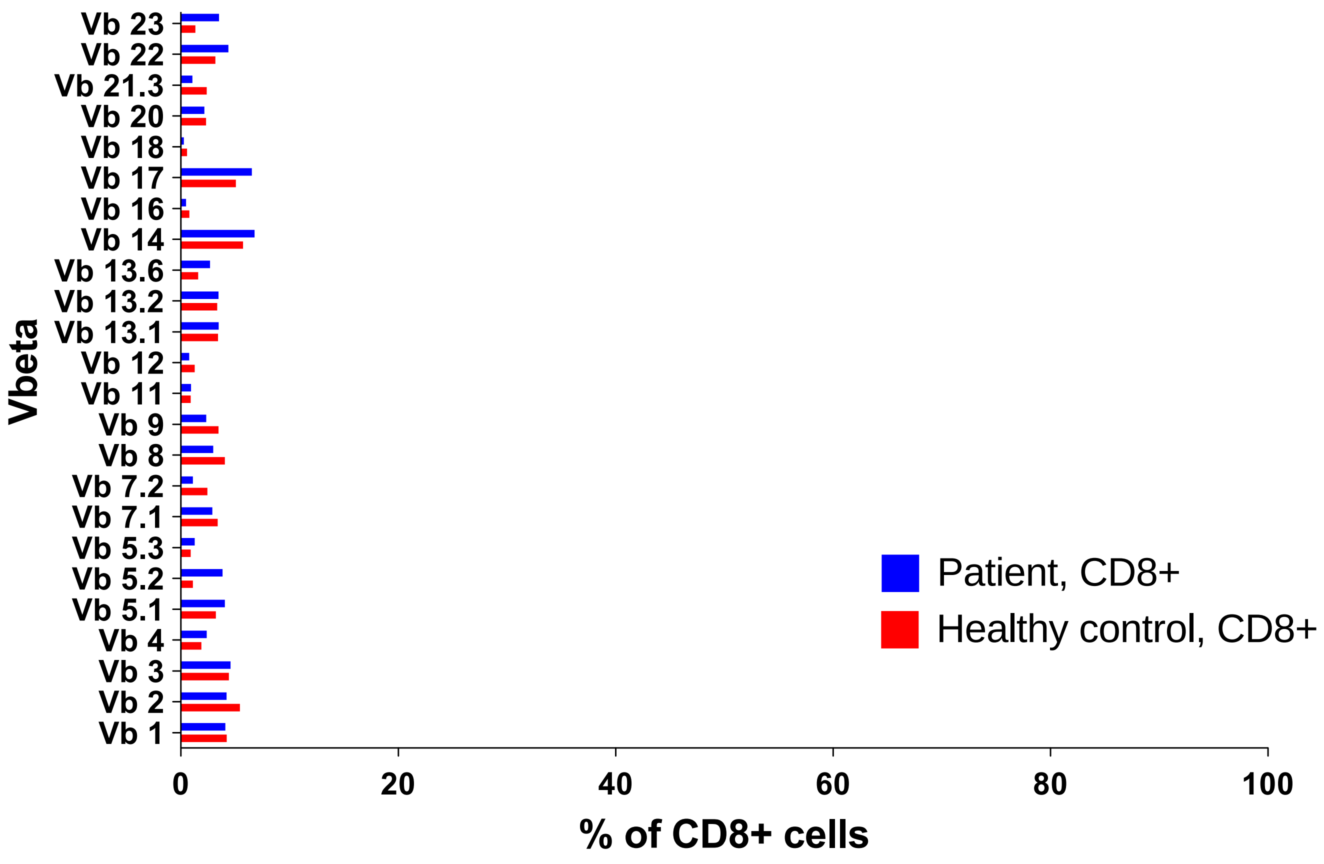
Vb 23
Vb 22
Vb 21.3
Vb 20
Vb 18
Vb 17
Vb 16
Vb 14
Vb 13.6
Vb 13.2
Vb 13.1
Vb 12
Vb 11
Vb 9
Vb 8
Vb 7.2
Vb 7.1
Vb 5.3
Vb 5.2
Vb 5.1
Vb 4
Vb 3
Vb 2
Vb 1
Vbeta
Patient, CD8+
Healthy control, CD8+
0
20
40
60
80
100
% of CD8+ cells

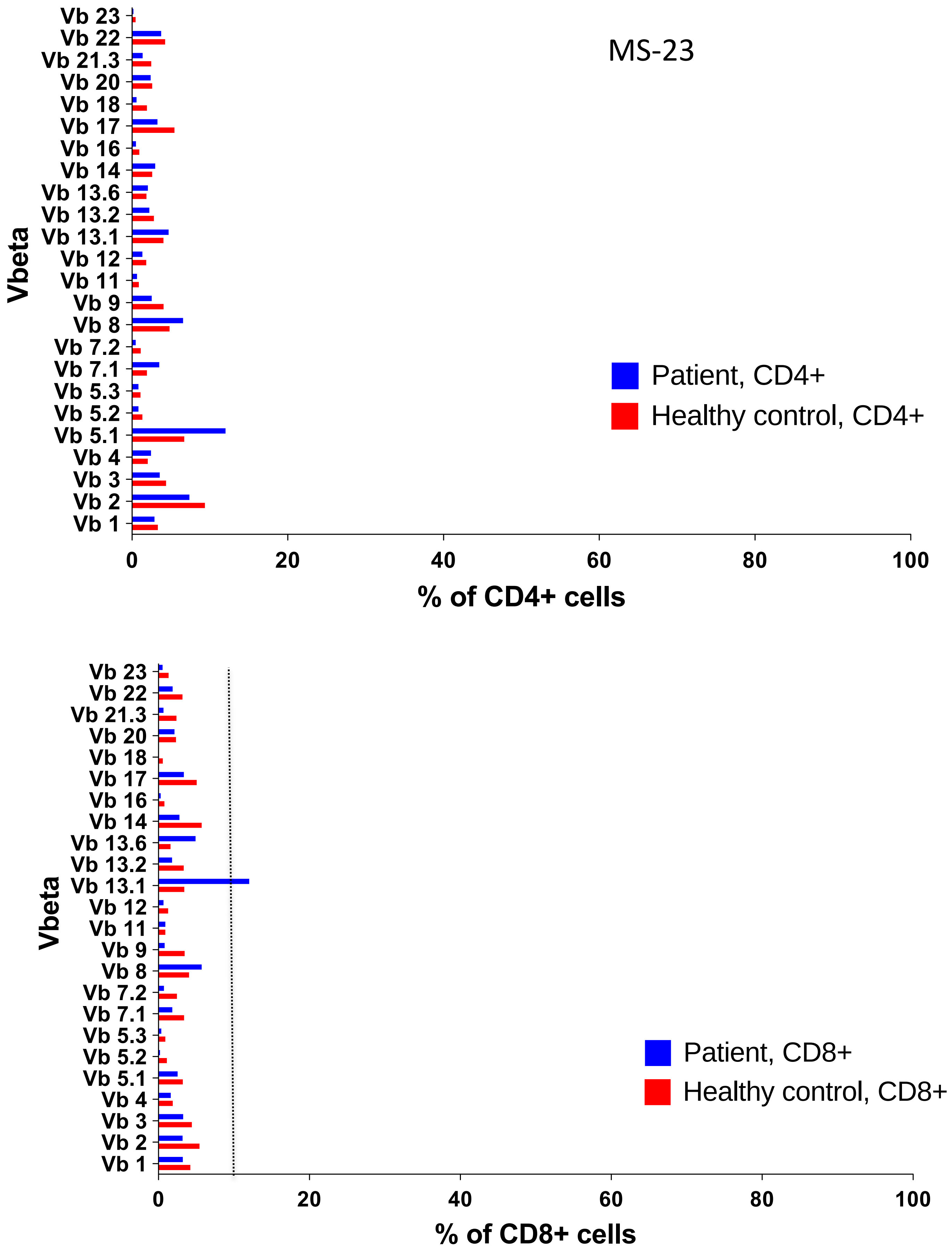
MS-23
Vbeta
Vb 23
Vb 22
Vb 21.3
Vb 20
Vb 18
Vb 17
Vb 16
Vb 14
Vb 13.6
Vb 13.2
Vb 13.1
Vb 12
Vb 11
Vb 9
Vb 8
Vb 7.2
Vb 7.1
Vb 5.3
Vb 5.2
Vb 5.1
Vb 4
Vb 3
Vb 2
Vb 1
0
20
40
60
80
100
% of CD4+ cells
Patient, CD4+
Healthy control, CD4+
Vbeta
Vb 23
Vb 22
Vb 21.3
Vb 20
Vb 18
Vb 17
Vb 16
Vb 14
Vb 13.6
Vb 13.2
Vb 13.1
Vb 12
Vb 11
Vb 9
Vb 8
Vb 7.2
Vb 7.1
Vb 5.3
Vb 5.2
Vb 5.1
Vb 4
Vb 3
Vb 2
Vb 1
0
20
40
60
80
100
% of CD8+ cells
Patient, CD8+
Healthy control, CD8+

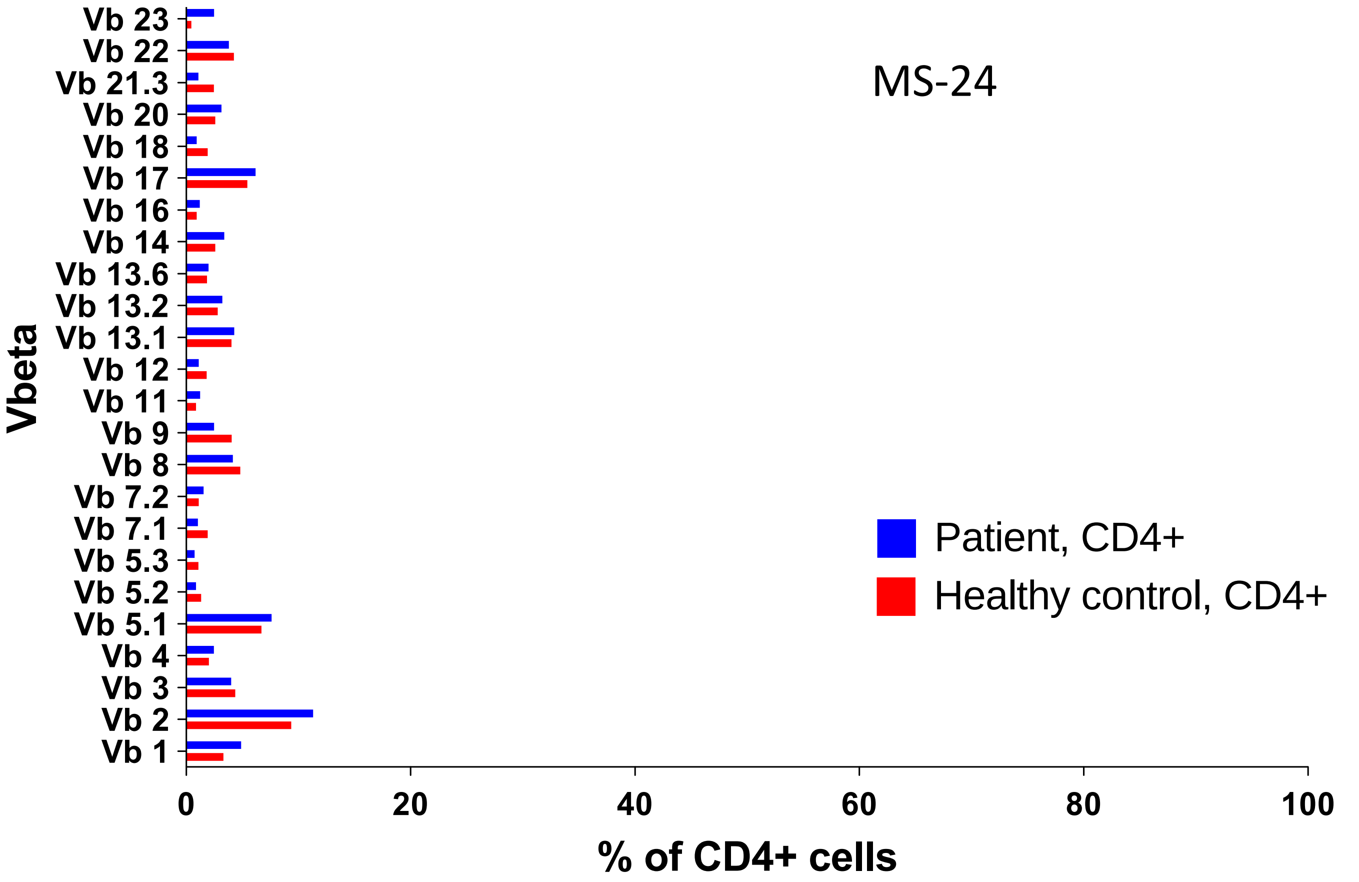
MS-24
Vbeta
Vb 23
Vb 22
Vb 21.3
Vb 20
Vb 18
Vb 17
Vb 16
Vb 14
Vb 13.6
Vb 13.2
Vb 13.1
Vb 12
Vb 11
Vb 9
Vb 8
Vb 7.2
Vb 7.1
Vb 5.3
Vb 5.2
Vb 5.1
Vb 4
Vb 3
Vb 2
Vb 1
0
20
40
60
80
100
% of CD4+ cells
Patient, CD4+
Healthy control, CD4+

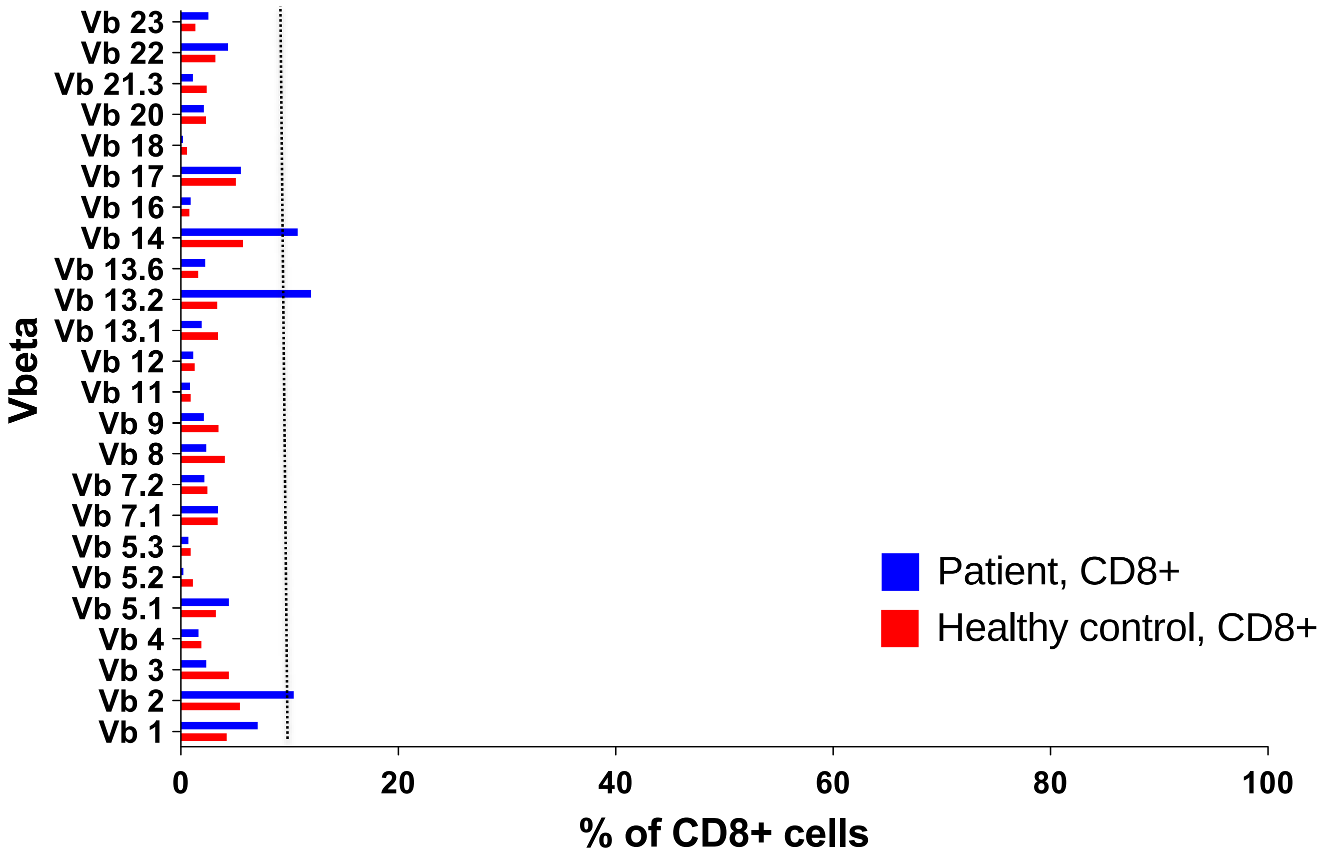
Vbeta
Vb 23
Vb 22
Vb 21.3
Vb 20
Vb 18
Vb 17
Vb 16
Vb 14
Vb 13.6
Vb 13.2
Vb 13.1
Vb 12
Vb 11
Vb 9
Vb 8
Vb 7.2
Vb 7.1
Vb 5.3
Vb 5.2
Vb 5.1
Vb 4
Vb 3
Vb 2
Vb 1
0
20
40
60
80
100
% of CD8+ cells
Patient, CD8+
Healthy control, CD8+
