# Supplementary material for: A novel class of somatic mutations in blood detected preferentially in CD8 + cells
Source: Clin Immunol. 2017 Feb;175:75–81. doi: 10.1016/j.clim.2016.11.018 (PMC5341785; doi:10.1016/j.clim.2016.11.018)
Supplement: Supplementary Table S2. — Genomic coordinates (GRCh37) for successfully validated somatic mutations and the level of supporting evidence. [file mmc4.pdf]

Supplementary table S2. Genomic coordinates (GRCh37) for successfully validated somatic mutations and the level of supporting evidence.

**Sample**	**Chrom**	**Coord**	**Ref**	**Alt**	**Gene**	**Screening P**	**Validation P**	**Ref bases (control cell populations, screening)**	**Alt bases (control cell populations, screening)**	**Ref bases (mutated cell population, screening)**	**Alt bases (mutated cell population, screening)**	**Ref bases (control cell populations, validation)**	**Alt bases (control cell populations, validation)**	**Ref bases (mutated cell population, validation)**	**Alt bases (mutated cell population, validation)**
MS-12-CD8+	chr1	158261127	C	T	CD1C	4E-11	< 1e-300	1427	1	324	16	117526	52	320337	15556
MS-8-CD19+	chr9	139818391	TCT	-	TRAF2	0.000003	< 1e-300	1264	4	719	20	70354	27	150616	6327
MS-19-CD8+	chr1	207940432	G	A	CD46	6E-16	< 1e-300	3324	1	1281	30	174468	48	196890	5843
MS-21-CD8+	chr3	50004928	C	T	RBM6	0.00000008	< 1e-300	1368	0	397	11	115382	11	162830	4710
MS-2-CD8+	chr12	9009912	C	T	A2ML1	0.000000003	< 1e-300	1235	0	720	20	242584	147	483573	13477
MS-1-CD8+	chr17	37947795	TAA	-	IKZF3	2E-10	< 1e-300	2812	1	1396	23	54753	17	272512	6347
MS-2-CD8+	chrX	100615139	C	T	BTK	0.0000009	< 1e-300	1160	0	594	13	137265	15	57027	1300
MS-19-CD8+	chr11	47593068	A	G	PTPMT1	0.000000002	< 1e-300	2395	0	933	16	148312	19	145518	2334
MG-5-others	chr11	113102448	G	A	NCAM1	0.003	< 1e-300	840	0	384	5	276168	312	137904	2208
MS-19-CD8+	chr5	52360880	C	A	ITGA2	0.00007	< 1e-300	3373	1	1273	9	168557	30	314335	4320
MS-21-CD8+	chr11	113092030	C	T	NCAM1	0.0001	< 1e-300	2824	1	926	8	148141	100	363658	4482
MS-8-CD8+	chr17	1783925	T	C	RPA1	3E-10	3E-181	1957	1	2697	46	47065	17	123775	1512
MS-8-CD8+	chr19	55377992	G	T	KIR3DL2	0.00002	< 1e-300	1042	0	1647	22	114203	17	263452	2608
MS-21-CD8+	chr15	60789727	C	T	RORA	0.00004	< 1e-300	2013	0	604	7	126265	66	175036	1685
MS-19-CD8+	chr19	43382428	A	T	PSG1	0.0002	6E-166	1347	0	561	7	58605	16	300348	2512
MS-3-CD8+	chr5	162902626	G	A	HMMR	0.0003	< 1e-300	1763	1	858	9	252568	37	197201	1659
NL-9-CD8+	chr5	41181496	C	G	C6	0.000008	3E-268	1601	0	831	11	148529	88	194722	1561
NL-9-CD8+	chr7	73811478	G	A	CLIP2	0.005	5E-45	1195	0	619	5	81557	199	109504	750
MS-1-CD8+	chr10	54531287	C	A	MBL2	0.0000005	< 1e-300	2491	0	1203	13	421676	356	277277	1643
MS-2-CD4+	chr5	66479095	C	A	CD180	0.001	1E-45	1761	0	801	6	26756	3	180517	838
MS-21-CD8+	chr17	40474420	C	A	STAT3	0.0005	1E-221	2865	1	992	7	203335	36	194678	888
MS-22-CD8+	chr19	7267652	G	A	INSR	0.0007	1E-158	2750	2	1207	9	185062	85	367086	1465
MS-14-CD8+	chr17	45363698	C	A	ITGB3	0.00004	3E-10	3998	0	1953	9	9627	4	94422	336
MS-8-CD8+	chrX	12904452	T	G	TLR7	0.0006	2E-111	1860	0	2441	13	171070	51	188771	611
MS-3-CD8+	chr4	86988964	G	A	MAPK10	0.001	1E-88	1993	0	994	6	261493	77	170927	401
MS-12-others	chr11	108202210	C	G	ATM	0.0003	6E-125	1529	0	368	5	210254	19	725857	1401
MS-23-CD8+	chr1	196659252	G	T	CFH	0.0003	6E-26	4212	2	1601	9	64224	16	140500	262

Displayed are both screening and validation step p-values (Fisher's exact test) and the number of reference and mutated bases.
For the screening step, the control samples are combined cell populations other than the mutated population from the same patient. For the validation step, the control sample is DNA from a donor (different person).
