# Supplementary material for: A novel class of somatic mutations in blood detected preferentially in CD8 + cells
Source: Clin Immunol. 2017 Feb;175:75–81. doi: 10.1016/j.clim.2016.11.018 (PMC5341785; doi:10.1016/j.clim.2016.11.018)
Supplement: Supplementary Table S3. — Read count data for whole blood somatic mutation amplicons. [file mmc5.pdf]

Supplementary table S3. Read count data for whole blood somatic mutation amplicons

Sample	Chrom	Coord	Ref	Alt	Gene	Allelic fraction	P	Ref bases (control DNA)	Alt bases (control DNA)	Ref bases (patient DNA, replicate 1)	Alt bases (patient DNA, replicate 1)	Ref bases (patient DNA, replicate 2)	Alt bases (patient DNA, replicate 2)
MS-12-CD8+	chr1	158261127	C	T	CD1C	0.36%	< 1e-300	1037261	171	944802	3090	752978	2916
MS-8-CD19+	chr9	139818391	TCT	-	TRAF2	0.39%	< 1e-300	1191470	6	801373	3068	804987	3208
MS-2-CD8+	chr12	9009912	C	T	A2ML1	0.56%	< 1e-300	958847	227	707884	4663	643388	3035
MS-2-CD8+	chrX	100615139	C	T	BTK	0.41%	< 1e-300	860625	90	821202	3676	693546	2493
MG-5-others	chr11	113102448	G	A	NCAM1	0.55%	< 1e-300	1071779	299	780453	3414	617710	4016
MS-8-CD8+	chr17	1783925	T	C	RPA1	0.06%	1.33754E-81	980190	123	920349	594	773720	412
MS-3-CD8+	chr5	162902626	G	A	HMMR	0.17%	< 1e-300	1080126	71	917129	1865	724841	1032
MS-2-CD4+	chr5	66479095	C	A	CD180	0.33%	< 1e-300	985623	86	1023834	3712	724164	2127
