# Supplementary material for: A novel class of somatic mutations in blood detected preferentially in CD8 + cells
Source: Clin Immunol. 2017 Feb;175:75–81. doi: 10.1016/j.clim.2016.11.018 (PMC5341785; doi:10.1016/j.clim.2016.11.018)
Supplement: Supplementary Table S4. — RNA-Sequencing FPKM expression values for the 27 genes in which somatic mutations were detected. Genes were considered expressed, when the FPKM value > 1 was reached, The values serve as a reference of gene expression in specific cell populations separated using our protocol. As such these values do not represent the expression status of the mutated allele. All target cell populations were analysed in two patients (MS17 and MS0). CD8 + basal expression was analysed in two additional patients (MS-7 and MS-8) and CD8 + expression after stimulation in one patient (MS-8). [file mmc6.zip › Supplementary table S4.pdf]

Supplementary table S4. Presence of large clones in CD8+ patient cell populations

Patient	Large clone present	Mutations detected
MS-1	no	yes
MS-2	yes	yes
MS-3	no	yes
MS-4	no	no
MG-5	yes	yes
MS-6	no	no
MS-7	yes	no
MS-8	no	yes
NL-9	no	yes
MS-10	no	no
MS-11	no	no
MS-12	yes	yes
MS-14	yes	yes
MS-17	yes	no
MS-19	yes	yes
MS-21	yes	yes
MS-22	no	yes
MS-23	yes	yes
MS-24	yes	no

Large clones are reported present if T cell receptor Vβ FACS analysis showed at least 10% enrichment for a particular Vβ type and the enrichment was also over a population control value supplied by the kit manufacturer.
