# Supplementary material for: A novel class of somatic mutations in blood detected preferentially in CD8 + cells
Source: Clin Immunol. 2017 Feb;175:75–81. doi: 10.1016/j.clim.2016.11.018 (PMC5341785; doi:10.1016/j.clim.2016.11.018)
Supplement: Supplementary Table S5. — Presence of large clones in CD8 + patient cell populations. [file mmc7.pdf]

Supplementary table S5. Presence of large clones in CD8+ patient cell populations

Patient	Large clone present	Mutations detected
MS-1	no	yes
MS-2	yes	yes
MS-3	no	yes
MS-4	no	no
MG-5	yes	yes
MS-6	no	no
MS-7	yes	no
MS-8	no	yes
NL-9	no	yes
MS-10	no	no
MS-11	no	no
MS-12	yes	yes
MS-14	yes	yes
NL-16	no	no
MS-17	yes	no
MS-19	yes	yes
MS-21	yes	yes
MS-22	no	yes
MS-23	yes	yes
MS-24	yes	no

Large clones are reported present if T cell receptor Vβ FACS analysis showed at least 10% enrichment for a particular Vβ type and the enrichment was also over a population control value supplied by the kit manufacturer.
The only patients with a mutation in CD4+ cells did not have a large clone in CD4
