# Supplementary material for: A novel class of somatic mutations in blood detected preferentially in CD8 + cells
Source: Clin Immunol. 2017 Feb;175:75–81. doi: 10.1016/j.clim.2016.11.018 (PMC5341785; doi:10.1016/j.clim.2016.11.018)
Supplement: Supplementary Table S6. — Somatic mutation persistence over time. [file mmc8.pdf]

Supplementary table S6. Somatic mutation persistence over time

Patient and cell population	Mutated gene	Sampling interval (months)	Allelic fraction at first sampling	Allelic fraction at second sampling	Ratio (2nd:1st sample)
MS-12-CD8+	CD1C	35	4.63%	3.71%	0.8
MS-8-CD19+	TRAF2	33	4.03%	0.00%	0
MS-19-CD8+	CD46	27	2.88%	3.53%	1.2
MS-21-CD8+	RBM6	19	2.81%	2.23%	0.8
MS-2-CD8+	A2ML1	28	2.71%	1.72%	0.6
MS-1-CD8+	IKZF3	24	2.28%	16.40%	7.2
MS-2-CD8+	BTK	28	2.23%	1.10%	0.5
MS-19-CD8+	PTPMT1	27	1.58%	0.87%	0.6
MG-5-others	NCAM1	35	1.58%	1.32%	0.8
MS-19-CD8+	ITGA2	27	1.36%	1.95%	1.4
MS-21-CD8+	NCAM1	19	1.22%	0.56%	0.5
MS-8-CD8+	RPA1	33	1.21%	1.88%	1.6
MS-8-CD8+	KIR3DL2	33	0.98%	1.77%	1.8
MS-21-CD8+	RORA	19	0.95%	0.48%	0.5
MS-19-CD8+	PSG1	27	0.83%	1.29%	1.6
MS-3-CD8+	HMMR	34	0.83%	0.48%	0.6
NL-9-CD8+	C6	33	0.80%	0.88%	1.1
NL-9-CD8+	CLIP2	33	0.68%	0.73%	1.1
MS-1-CD8+	MBL2	24	0.59%	0.20%	0.3
MS-2-CD4+	CD180	28	0.46%	0.37%	0.8
MS-21-CD8+	STAT3	19	0.45%	0.74%	1.6
MS-22-CD8+	INSR	24	0.40%	1.43%	3.6
MS-14-CD8+	ITGB3	23	0.35%	0.33%	0.9
MS-8-CD8+	TLR7	33	0.32%	0.35%	1.1
MS-3-CD8+	MAPK10	34	0.23%	0.23%	1
MS-12-others	ATM	35	0.19%	0.04%	0.2
MS-23-CD8+	CFH	17	0.19%	0.46%	2.4

Allelic fractions at both time points are determined from amplicon sequencing data. The label “others” denotes the CD4-CD8-CD19- population.
