# Supplementary material for: A novel class of somatic mutations in blood detected preferentially in CD8 + cells
Source: Clin Immunol. 2017 Feb;175:75–81. doi: 10.1016/j.clim.2016.11.018 (PMC5341785; doi:10.1016/j.clim.2016.11.018)
Supplement: Supplementary Table S7. — Somatic mutation persistence read count data (amplicon sequencing). [file mmc9.pdf]

Supplementary table S7. Somatic mutation persistence read count data (amplicon sequencing)

Sample	Chrom	Coord	Ref	Alt	Gene	P	Ref bases (control cell population)	Alt bases (control cell population)	Ref bases (mutated cell population)	Alt bases (mutated cell population)
MS-12-CD8+	chr1	158261127	C	T	CD1C	< 1e-300	142950	83	145349	5601
MS-8-CD19+	chr9	139818391	TCT	-	TRAF2	3.E-01	125228	0	182530	3
MS-19-CD8+	chr1	207940432	G	A	CD46	< 1e-300	168831	71	170072	6216
MS-21-CD8+	chr3	50004928	C	T	RBM6	< 1e-300	126993	9	170099	3881
MS-2-CD8+	chr12	9009912	C	T	A2ML1	< 1e-300	135355	84	133753	2334
MS-1-CD8+	chr17	37947795	TAA	-	IKZF3	< 1e-300	126609	6	154213	30255
MS-2-CD8+	chrX	100615139	C	T	BTK	< 1e-300	133149	24	108795	1210
MS-19-CD8+	chr11	47593068	A	G	PTPMT1	< 1e-300	126300	8	154785	1364
MG-5-others	chr11	113102448	G	A	NCAM1	5.E-253	98388	139	115600	1549
MS-19-CD8+	chr5	52360880	C	A	ITGA2	< 1e-300	145605	14	151011	3010
MS-21-CD8+	chr11	113092030	C	T	NCAM1	1.E-102	106953	83	117828	669
MS-8-CD8+	chr17	1783925	T	C	RPA1	< 1e-300	101527	35	120546	2306
MS-8-CD8+	chr19	55377992	G	T	KIR3DL2	< 1e-300	107419	17	83738	1510
MS-21-CD8+	chr15	60789727	C	T	RORA	1.E-75	105585	76	89457	434
MS-19-CD8+	chr19	43382428	A	T	PSG1	1.E-274	73839	7	73304	960
MS-3-CD8+	chr5	162902626	G	A	HMMR	1.E-123	95190	11	103002	499
NL-9-CD8+	chr5	41181496	C	G	C6	< 1e-300	186571	2	200716	1775
NL-9-CD8+	chr7	73811478	G	A	CLIP2	2.E-34	53912	134	73885	541
MS-1-CD8+	chr10	54531287	C	A	MBL2	2.E-71	170134	28	177752	360
MS-2-CD4+	chr5	66479095	C	A	CD180	2.E-92	109846	14	85202	313
MS-21-CD8+	chr17	40474420	C	A	STAT3	6.E-264	125566	19	154679	1158
MS-22-CD8+	chr19	7267652	G	A	INSR	< 1e-300	144799	71	139537	2027
MS-14-CD8+	chr17	45363698	C	A	ITGB3	1.E-62	67203	3	65830	221
MS-8-CD8+	chrX	12904452	T	G	TLR7	9.E-197	177540	0	200498	713
MS-3-CD8+	chr4	86988964	G	A	MAPK10	5.E-61	141131	37	175050	395
MS-12-others	chr11	108202210	C	G	ATM	6.E-11	124960	6	126945	55
MS-23-CD8+	chr1	196659252	G	T	CFH	3.E-190	141244	10	171522	801
