# Supplementary material for: A novel class of somatic mutations in blood detected preferentially in CD8 + cells
Source: Clin Immunol. 2017 Feb;175:75–81. doi: 10.1016/j.clim.2016.11.018 (PMC5341785; doi:10.1016/j.clim.2016.11.018)
Supplement: Supplementary Table S8. — RNA-Seq FPKM expression values for genes in which somatic mutations were detected in the study. [file mmc10.zip › Supplementary table S8.pdf]

**Supplementary table S8**
**Screening phase sequencing depths for successfully validated somatic variants**

| | Min. | 1st Quartile | Median | 3rd Quartile | Max. |
|---|---|---|---|---|---|
| **Mutated cell population** | 340 | 617 | 934 | 1296 | 2743 |
| **Control cell populations** | 840 | 1358 | 1860 | 2782 | 4214 |

The distribution of sequencing depths in the screening phase
(mutated cell population vs. control cell populations of the same patient)
